# Rosace: a robust deep mutational scanning analysis framework employing position and mean-variance shrinkage

**DOI:** 10.1186/s13059-024-03279-7

**Published:** 2024-05-24

**Authors:** Jingyou Rao, Ruiqi Xin, Christian Macdonald, Matthew K. Howard, Gabriella O. Estevam, Sook Wah Yee, Mingsen Wang, James S. Fraser, Willow Coyote-Maestas, Harold Pimentel

**Affiliations:** 1 Department of Computer Science, UCLA, Los Angeles, CA USA; 2 Computational and Systems Biology Interdepartmental Program, UCLA, Los Angeles, CA USA; 3 Department of Bioengineering and Therapeutic Sciences, UCSF, San Francisco, CA USA; 4 Tetrad Graduate Program, UCSF, San Francisco, CA USA; 5 Department of Pharmaceutical Chemistry, UCSF, San Francisco, CA USA; 6 Department of Mathematics, Baruch College, CUNY, New York, NY USA; 7 Quantitative Biosciences Institute, UCSF, San Francisco, CA USA; 8 Department of Computational Medicine, David Geffen School of Medicine, UCLA, Los Angeles, CA USA; 9 Department of Human Genetics, David Geffen School of Medicine, UCLA, Los Angeles, CA USA

## Abstract

Deep mutational scanning (DMS) measures the effects of thousands of genetic variants in a protein simultaneously. The small sample size renders classical statistical methods ineffective. For example, *p*-values cannot be correctly calibrated when treating variants independently. We propose Rosace, a Bayesian framework for analyzing growth-based DMS data. Rosace leverages amino acid position information to increase power and control the false discovery rate by sharing information across parameters via shrinkage. We also developed Rosette for simulating the distributional properties of DMS. We show that Rosace is robust to the violation of model assumptions and is more powerful than existing tools.

**Supplementary Information:**

The online version contains supplementary material available at 10.1186/s13059-024-03279-7.

## Background

Understanding how protein function is encoded at the residue level is a central challenge in modern protein science. Mutations can cause diseases and drive evolution through perturbing protein function in a myriad of ways, such as by altering its conformational ensemble and stability or its interaction with ligands and binding partners. In these contexts, mutations may result in a loss of function, gain of function, or a neutral phenotype (i.e., no discernable effects). Mutations also often exert effects across multiple phenotypes, and these perturbations can ultimately propagate to alter complex processes in cell biology and physiology. Reverse genetics approaches offer a powerful handle for researchers to investigate biology via introducing mutations and observing the resulting phenotypic changes.

**Fig. 1:**
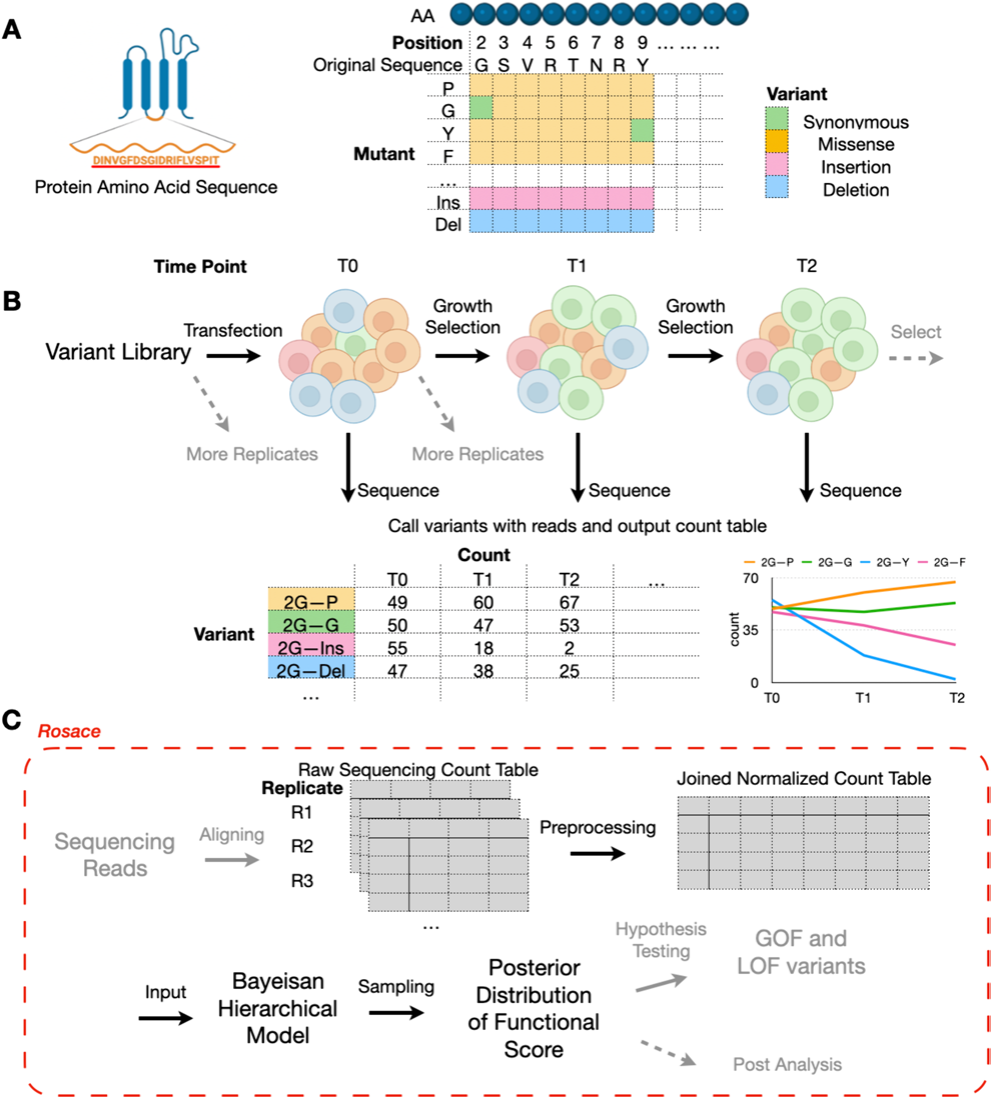
Deep mutational scanning and overview of Rosace framework. **A** Each amino acid of the selected protein sequence is mutated to another mutant in deep mutational scanning. **B** Cells carrying different variants are grown in the same pool under selection pressure. At each time point, cells are sequenced to output the count table. Replications can be produced either pre-transfection or post-transfection. **C** Rosace is an R package that accepts input from the raw sequencing count table and outputs the posterior distribution of functional score

Deep mutational scanning (DMS) is a technique for systematically determining the effect of a large library of mutations individually on a phenotype of interest by performing pooled assays and measuring the relative effects of each variant (Fig. 1A) [1–3]. It has improved clinical variant interpretation [4] and provided insights into the biophysical modeling and mechanistic models of genetic variants [5]. Taking enzymes as an example, these phenotypes could include catalytic activity [6] or stability [7, 8]. For a transcription factor, the phenotype could be DNA binding specificity or transcriptional activity [9]. The relevant phenotype for a membrane transporter might be folding and trafficking or substrate transport [10]. These phenotypes are often captured by growth-based [7, 10–16], binding-based [9, 17, 18], or fluorescence-based assays [8, 10, 19]. Those experiments are inherently differently designed and merit separate analysis frameworks. In growth-based assays, the relative growth rates of cells are of interest. In a binding-based assay, the selection probabilities are of interest. In fluorescence-based assays, changes to the distribution of reporter gene expression are measured. In this paper, we focus solely on growth-based screens.

In a growth-based DMS experiment, we grow a pool of cells carrying different variants under a selective pressure linked to gene function. At set intervals, we sequence the cells to identify each variant’s frequency in the pool. The change in the frequency over the course of the experiment, from initial frequencies to subsequent measurements, serves as a metric of the variant’s functional effects (Fig. 1B). The functional score is often computed for each variant in the DMS screen and compared against those of synonymous mutations or wild-type cells to display the relative functional change of the protein caused by the mutation. Thus, reliable inference of functional scores is crucial to understanding both individual mutations and at which residue location variants tend to have significant functional effects.

The main challenge of functional score inference is that even under the simplest model, there are at least two estimators required for each mutation (mean and variance of functional change), and in practice, it is rare to have more than three replicates. As a result, it has been posited that under naïve estimators that have been commonly employed, there are likely issues with the false discovery rate and the statistical power of detecting mutations that significantly change the function of the protein [20]. Regardless, incorporating domain-specific assumptions is required to make inference tractable with few samples and thousands of parameters.

To alleviate the small-sample-size inference problem in DMS, four commonly used methods have been developed: *dms_tools* [21], *Enrich2* [18], *DiMSum* [20], and *EMPIRIC* [22]. *dms_tools* uses Bayesian inference for reliable inference. However, rather than giving a score to each variant, *dms_tools* generates a score for each amino acid at each position, assuming linear addition of multiple mutation effects and ignoring epistasis coupling. Thus, *dms_tools* is not directly comparable to other methods and is excluded from our benchmarking analysis. *Enrich2* simplifies the variance estimator by assuming that counts are Poisson-distributed (the variance being equal to the mean) and combines the replicates using a random-effect model. *DiMSum*, however, argues that the assumption in *Enrich2* is not enough to control type-I error. As a result, *DiMSum* builds upon *Enrich2* and includes additional variance terms to model the over-dispersion of sequencing counts. However, as presented in Faure et al. 2020 [20], this ratio-based method only applies to the DMS screen with one round of selection, while many DMS screens have more than two rounds of selection (i.e., sampling at multiple time points) [10, 11, 23]. Alternatively, *EMPIRIC* fits a Bayesian model that infers each variant separately with non-informative uniform prior to all parameters and thus does not shrink the estimates to robustly correct the variance in estimates due to the small sample size. Further, the model does not accommodate multiple replicates. In addition, *mutscan* [24], a recently developed R package for DMS analysis, employed two established statistical models *edgeR* and *limma-voom*. However, these two methods were originally designed for RNA-seq data and the data generation process for DMS is very different. One of the key differences is consistency among replicates. In RNA-seq, gene expression is relatively consistent across replicates under the same condition, while in DMS, counts of variants can vary much since the a priori representation in the initial variant library can be vastly inconsistent among replicates.

While these methods provide reasonable regularization of the score’s variance, additional information can further improve the prior. One solution is incorporating residue position information. It has been noted that amino acids in particular regions have an oversized effect on the protein’s function, and other frameworks have incorporated positions for various purposes. In the form of hidden Markov models (HMMs) and position-specific scoring matrices (PSSMs), this is the basis for the sensitive detection of homology in protein sequences [25]. These results directly imply that variants at the same position likely share some similarities in their behavior and thus that incorporating local information into modeling might produce more robust inferences. However, no existing methods have incorporated residue position information into their models yet.

To overcome these limitations, we present Rosace, the first growth-based DMS method that incorporates local positional information to increase inference performance. Rosace implements a hierarchical model that parameterizes each variant’s effect as a function of the positional effect, thus providing a way to incorporate both position-specific information and shrinkage into the model. Additionally, we developed Rosette, a simulation framework that attempts to simulate several properties of DMS such as bimodality, similarities in behavior across similar substitutions, and the overdispersion of counts. Compared to previous simulation frameworks such as the one in *Enrich2*, Rosette uses parameters directly inferred from the specific input experiment and generates counts that reflect the true level of noise in the real experiment. We use Rosette to simulate several screening modalities and show that our inference method, Rosace, exhibits higher power and controls the false discovery rate (FDR) better on average than existing methods. Importantly, Rosace and Rosette are not two views of the same model—Rosette is based on a set of assumptions that are different from or even opposite to those of Rosace. Rosace’s ability to accommodate data generated under different assumptions shows its robustness. Finally, we run Rosace on real datasets and it shows a much lower FDR than existing methods while maintaining similar power on experimentally validated positive controls.

## Results

### Overview of Rosace framework

Rosace is a Bayesian framework for analyzing growth-based deep mutational scanning data, producing variant-level estimates from sequencing counts. The full (position-aware) method requires as input the raw sequencing counts and the position labels of variants. It outputs the posterior distribution of variants’ functional scores, which can be further evaluated to conduct hypothesis testing, plotting, and other downstream analyses (Fig. 1C). If the position label is hard to acquire with heuristics, for example, in the case of random multiple-mutation data, position-unaware Rosace model can be run without position label input. Rosace is available as an R package. To generate the input of Rosace from sequencing reads, we share a Snakemake workflow dubbed Dumpling for short-read-based experiments in the GitHub repository described in the “Methods” section. Additionally, Rosace supports input count data processed from *Enrich2* [18] for other protocols such as barcoded sequencing libraries.

### Rosace hierarchical model with positional information and score shrinkage

Here, we begin by motivating the use of positional information. Next, we describe the intuition of how we use the positional information. Finally, we describe the remaining dimensions of shrinkage which assist in robust estimates with few experiment replicates.

A variant is herein defined as the amino acid identity at a position in a protein, where that identity may differ from the wild-type sequence. In this context, synonymous, missense, nonsense, and indel variants are all considered and can be processed by Rosace (see the “Methods” section for details). The sequence position of a variant *p*(*v*) provides information on the functional effects to the protein from the variant. We define the position-level functional score $\phi _{p(v)}$ as the mean functional score of all variants on a given position.

**Fig. 2:**
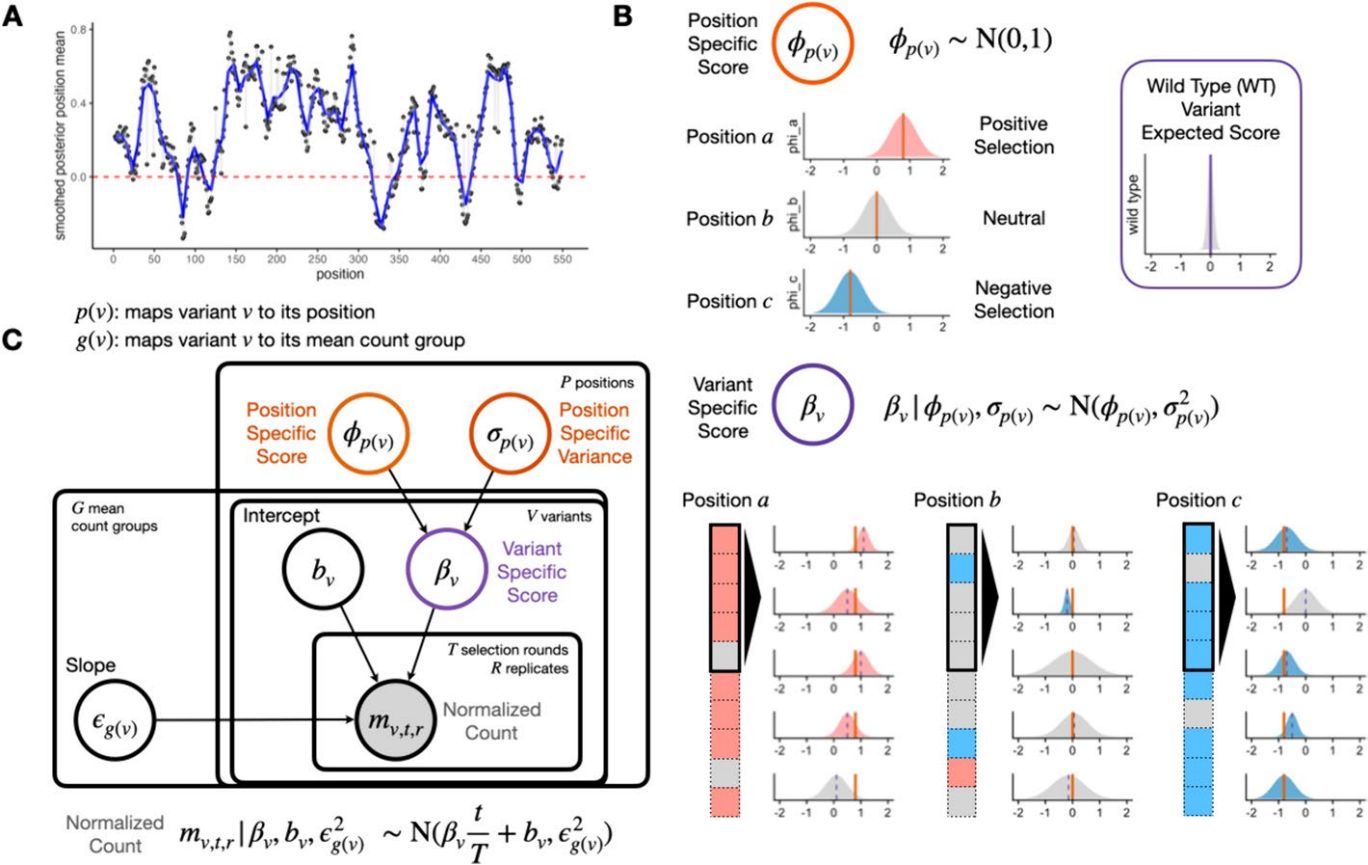
Rosace shares information at the same position to inform variant effects. **A** Smoothed position-specific score (sliding window = 5) across positions from OCT1 cytotoxicity screen. Red dotted lines at score = 0 (neutral position). **B** A conceptual view of the Rosace generative model. Each position has an overall effect, from which variant effects are conferred. Note the prior is wide enough to allow effects that do not follow the mean. Wild-type score distribution is assumed to be at 0. **C** Plate model representation of Rosace. See the “Methods” section for the description of parameters

To motivate the use of positional information, we take the posterior distribution of the position-level functional score estimated from a real DMS experiment, a cytotoxicity-based growth screen of a human transporter, OCT1 (Fig. 2A). In this experiment, variants with decreased activity are expected to increase in abundance, as they lose the ability to import a cytotoxic substrate during selection, and variants with increased activity will decrease in abundance similarly. We observe that most position-level score estimates $\widehat{\phi }_{p(v)}$ significantly deviate from the mean, implying that position has material idiosyncratic variation and thus carries information about the protein’s functional architecture.

To incorporate the positional information into our model, we introduce a position-specific score $\phi _{p(v)}$ where *p*(*v*) maps variant *v* to its amino acid position. The variant-specific score $\beta _v$ is regularized and controlled by the value of $\phi _{p(v)}$. To illustrate the point, we conceptually categorize position into three types: positively selected ($\phi _{p(v)} \gg 0$), (nearly) neutral ($\phi _{p(v)} \approx 0$), and negatively selected ($\phi _{p(v)} \ll 0$) (Fig. 2B). Variants in a positively selected position tend to have scores centered around the positive mean estimate of $\phi _{p(v)}$, and vice versa for the negatively selected position. Variants in a neutral position tend to be statistically non-significant as the region might not be important to the measured phenotype.

Regularization of the score’s variance is achieved mainly by sharing information across variants within the position and asserting weakly informative priors on the parameters (Fig. 2C). Functional scores of the variants within the position are drawn from the same set of parameters $\phi _{p(v)}$ and $\sigma _{p(v)}$. The error term $\epsilon _{g(v)}$ in the linear regression on normalized counts is also shared in the mean count group (see the “Methods” section) to prevent biased estimation of the error and incorporate mean-variance relationship commonly modeled in RNA-seq [26, 27]. Importantly, while we use the position information to center the prior, the prior is weak enough to allow variants at a position to deviate from the mean. For example, we show that the nonsense variants indeed deviate from the positional mean (Additional file 1: Fig. S3). The variant-level intercept $b_v$ is given a strong prior with a tight distribution centered at 0 to prevent over-fitting.

### Rosace performance on various datasets

To test the performance of Rosace, we ran Rosace along with *Enrich2*, *mutscan* (both *limma-voom* and *edgeR*), *DiMSum*, and simple linear regression (the naïve method) on the OCT1 cytotoxicity screen. *DiMSum* cannot analyze data with three selection rounds, so we ran *DiMSum* with only the first two time points. The data is pre-processed with wild-type normalization for all three methods. The analysis is done on all subsets of three replicates ($\{1\}, \{2\}, \{3\}, \{1,2\}, \{1,3\}, \{2,3\}, \{1,2,3\}$).

**Fig. 3:**
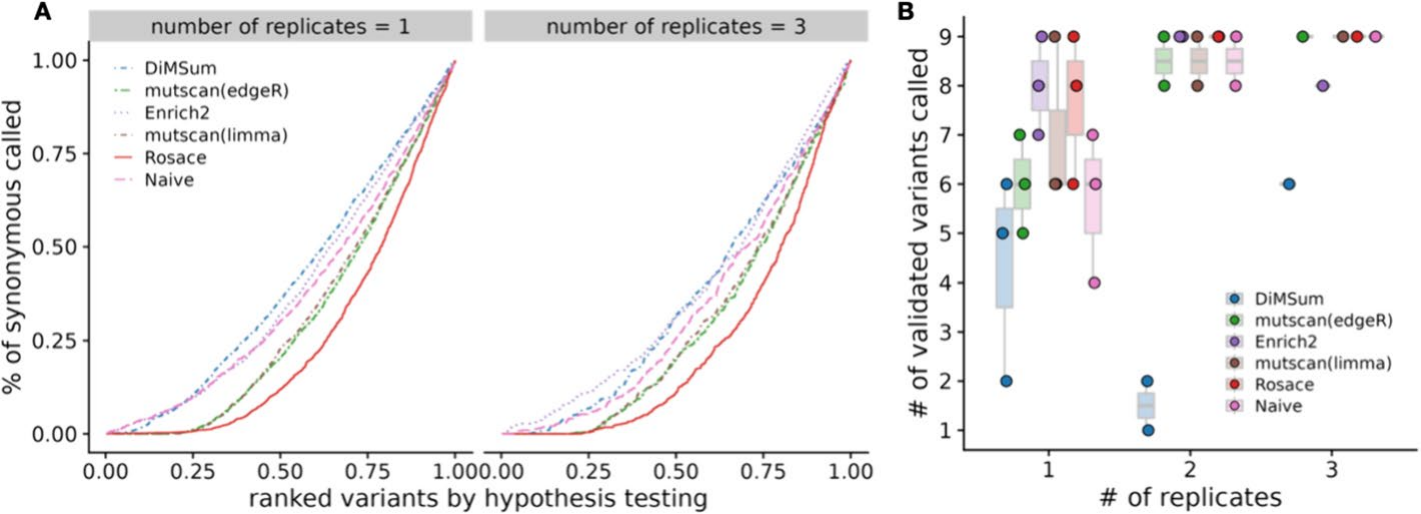
False discovery rate and sensitivity on OCT1 cytotoxicity data. **A** Percent of synonymous mutations called (false discovery rate) versus ranked variants by hypothesis testing. The left panel is from taking the mean of analysis of the three individual replicates. Ideally, the line would be flat at 0 until all the variants with true effects are discovered. **B** Number of validated variants called (in total 10) versus number of replicates. If only 1 or 2 replicates are used, we iterate through all possible combinations. For example, the three points for Rosace on 2 replicates use Replicate $\{1, 2\}$, $\{1, 3\}$, and $\{2, 3\}$ respectively. (DiMSum can only process two time points, and thus is disadvantaged in experiments such as OCT1)

While we do not have a set of true negative control variants, we assume most synonymous mutations would not change the phenotype, and thus, we use synonymous mutation as a proxy for negative controls. We compute the percentage of significant synonymous mutations called by the hypothesis testing as one representation of the false discovery rate (FDR). The variants are ranked based on the hypothesis testing statistics from the method (*p*-value for frequentist methods and local false sign rate [28], or *lfsr*) for Bayesian methods). In an ideal scenario with no noise, the line of ranked variants by FDR is flat at 0 and slowly rises after all true variants with effect are called. Rosace has a very flat segment among the top 25% of the ranked variants compared to *DiMSum*, *Enrich2*, and the naïve method and keeps the FDR lower than *mutscan(limma)* and *mutscan(edgeR)* until the end (Fig. 3A). Importantly, we note that the Rosace curve moves only slightly from 1 replicate to 3 replicates, while the other methods shift more, implying that the change in the number of synonymous mutations called is minor for Rosace, despite having fewer replicates (Fig. 3A).

While lower FDR may result in lower power in the method, we show that Rosace is consistently powerful in detecting the OCT1-positive control variants. Yee et al. [10] conducted lower-throughput radioligand uptake experiments in HEK293T cells and validated 10 variants that have a loss-of-function or gain-of-function phenotype. We use the number of validated variants to approximate the power of the method. As shown in Fig. 3B, Rosace has comparable power to *Enrich2*, *mutscan(limma)*, and *mutscan(edgeR)* regardless of the number of replicates, while the naïve method is unable to detect anything in the case of one replicate. Rosace calls significantly fewer synonymous mutations than every other method while maintaining high power, showing that Rosace is robust in real data.

In OCT1, loss of function leads to enrichment rather than depletion, which is relatively uncommon. To complement findings on OCT1, we conducted a similar analysis on the kinase MET data [11] (3 replicates, 3 selection rounds), whose loss of function leads to depletion. Applied to this dataset, Rosace and its position-unaware version have comparable power to *Enrich2*, *mutscan(limma)*, and *mutscan(edgeR)* with any number of replicates used, and the naïve method remains less powerful than other methods, especially with one replicate only. Consistent with OCT1, Rosace again calls fewer synonymous mutations and better controls the false discovery rate. The results are visualized in the Supplementary Figures (Additional file 1: Figs. S12-15).

To test Rosace performance on diverse datasets, we also ran all methods on the CARD11 data [14] (5 replicates, 1 selection round), the MSH2 data [12] (3 replicates, 1 selection round), the BRCA1 data [13] (2 replicates, 2 selection rounds), and the BRCA1-RING data [23] (6 replicates, 5 selection rounds) (Table S1). In addition to those human protein datasets, we also applied Rosace to a bacterial protein, Cohesin [29] (1 replicate, 1 selection round) (Table S1). We use the pathogenic and benign variants in ClinVar [30], EVE [31], and AlphaMissense [32] to provide a proxy of positive and negative control variants. Rosace consistently shows high sensitivity in detecting the positive control variants in all three datasets while controlling the false discovery rate (Additional file 1: Figs. S5-S11). Noting that the number of clinically verified variants is limited and those identified in the prediction models usually have extreme effects, we do not observe a large difference between the methods’ performance.

To alleviate a potential concern that the position-level shrinkage given by Rosace is too large, we plot the functional scores calculated by Rosace against those by *Enrich2* across several DMS datasets (Additional file 1: Figs. S2-4). We find that the synonymous variants’ functional scores are similar in magnitude to those of other variants, so synonymous variants are not shrunken too strongly to zero. We also find that stop codon and indel variants have consistently significant effect scores, implying that position-level shrinkage is not so strong that those variants’ effects are neutralized. This result implies that the position prior benefits the model mainly through a more stable standard error estimate enabling improved prioritization as a function of local false sign rate or other posterior ranking criteria that are a function of the variance.

### Rosette: DMS data simulation which matches marginal distributions from real DMS data

**Fig. 4:**
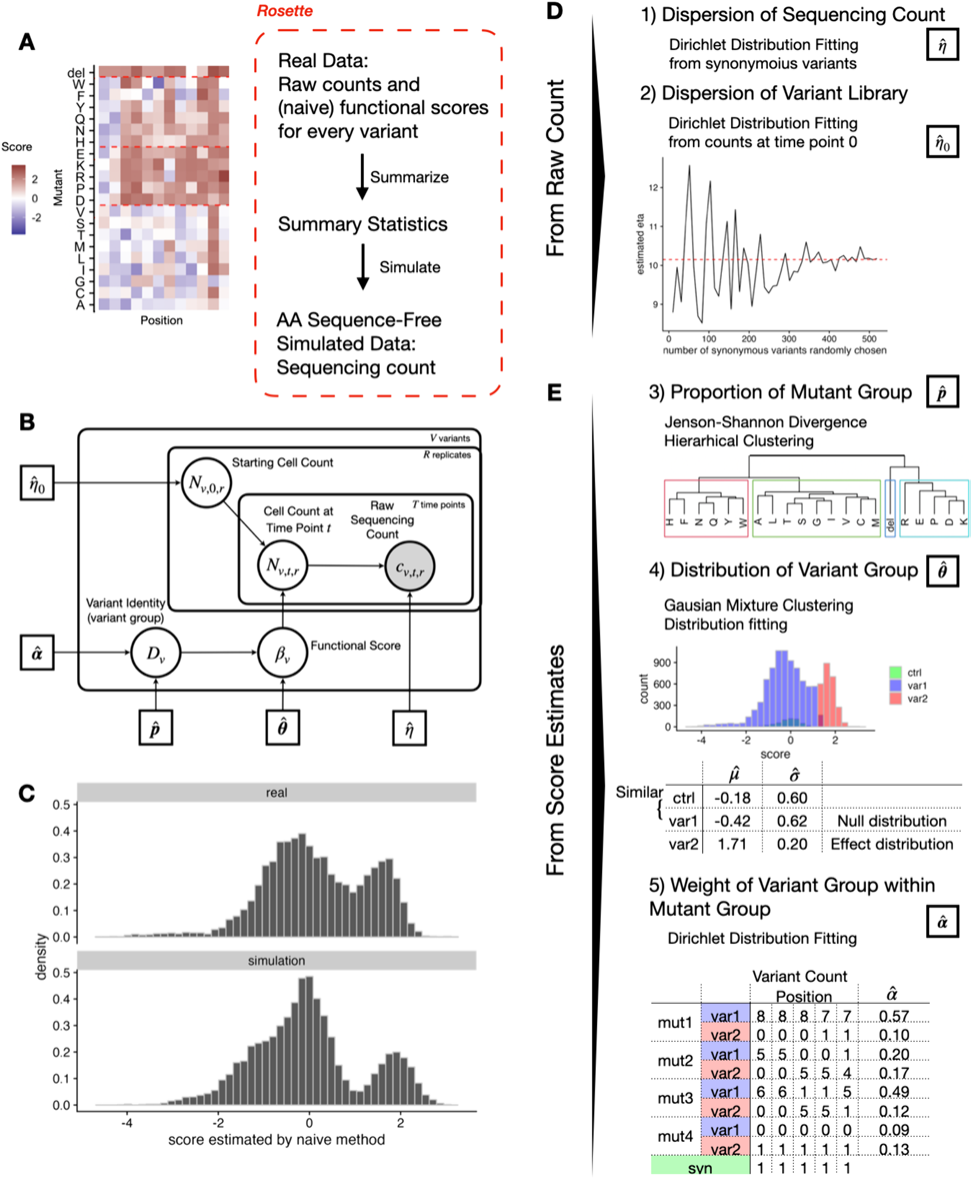
Rosette simulation framework preserves the overall structure of growth-based DMS screens. The plots show the result of using OCT1 data as input. **A** Rosette generates summary statistics from real data and simulates the sequencing count. **B** Generative model for Rosette simulation. **C** The distribution of real and predicted functional scores is similar. **D**, **E** Five summary statistics are needed for Rosette

To further benchmark the performance of Rosace and other related methods, we propose a new simulation framework called Rosette, which generates DMS data using parameters directly inferred from the real experiment to gain the flexibility of mimicking the overall structure of most growth-based DMS screen data (Fig. 4A).

Intuitively, if we construct a simulation that closely follows the assumptions of our model, our model should have outstanding performance. To facilitate a fair comparison with other methods, the simulation presented here is not aligned with the assumptions made in Rosace. In fact, the central assumption that variant position carries information is violated by construction to showcase the robustness of Rosace.

To re-clarify the terminology used throughout this paper, “mutant” refers to the substitution, insertion, or deletion of amino acids. A position-mutant pair is considered a variant. Mutants are categorized into mutant groups with hierarchical clustering schemes or predefined criteria (our model uses the former that are expected to align with the biophysical properties of amino acids). Variants are grouped in two ways: (1) by their functional change to the protein, namely neutral, loss-of-function (LOF), or gain-of-function (GOF), referred to as “variant groups,” and (2) by the mean of the raw sequencing counts across replicates, referred to as “variant mean groups.”

Rosette calculates two summary statistics from the raw sequencing counts (dispersion of the sequencing count $\eta$ and dispersion of the variant library $\eta _0$) (Fig. 4D) and three others from the score estimates (the proportion of each mutant group $\varvec{p}$, the functional score’s distribution of each variant group $\varvec{\theta }$, and the weight of each variant group $\varvec{\alpha }$) (Fig. 4E). Since we are only learning the distribution of the scores instead of the functional characteristics of individual variants, the score estimates can be naïve (e.g., simple linear regression) or more complicated (e.g., Rosace).

The dispersion of the sequencing counts $\eta$ measures how much variability in variant representation there is in the entire experimental procedure, during both cell culture and sequencing. When $\eta$ goes to infinity, it means that the sequencing count is almost the same as the expected true cell count (no over-dispersion). When $\eta$ is small, it shows an over-dispersion of the sequencing count. In an ideal experiment with no over-dispersion, the proportion of synonymous mutations should be invariant to time due to the absence of functional changes. However, from the real data, we have observed a large variability of proportion changes within the synonymous mutations at different selection rounds, which is attributed to over-dispersion and cannot be explained by a simple multinomial distribution in existing simulation frameworks (Additional file 1: Fig. S1). Indeed, all methods, including the naïve method, achieve near-perfect performance in the *Enrich2* simulations with a correlation score greater than 0.99 (Additional file 1: Fig. S27). Therefore, we choose to model the sequencing step with a Dirichlet-Multinomial distribution that includes $\eta$ as the dispersion parameter.

The dispersion of variant library $\eta _0$ measures how much variability already exists in variant representation before the cell selection. Theoretically, each variant would have around the same number of cells at the initial time point. However, due to the imbalance during the variant library generation process and the cell culture of the initial population that might already be under selection, we sometimes see a wide dispersion of counts across variants. To estimate this dispersion, we fit a Dirichlet-Multinomial distribution under the assumption that the variants in the cell pool at the initial time point should have equal proportions.

The distribution and the structure of the underlying true functional score across variants are controlled by the rest of the summary statistics. We make a few assumptions here. First, the functional score distribution of mutants across positions (or a row in the heatmap (Fig. 4A)) is different, but within the mutant group, the mutants are independent and identically distributed (or exchangeable). We estimate the mutant group by hierarchical clustering with distance defined by empirical Jenson-Shannon Divergence and record its proportion $\hat{\varvec{p}}$. Second, each variant belongs to the neutral hypothesis (score close to 0, similar to synonymous mutations) or the alternative hypothesis (away from 0, different from synonymous mutations). The number of the variant group can be 1–3 (neutral, GOF, and LOF) based on the number of modes in the marginal functional score distribution, and the variants within a variant group are exchangeable. We estimate the borderline of the variant group by Gaussian mixture clustering and fit the distribution parameter $\hat{\varvec{\theta }}$. Finally, we assume that the positions are independent. While this is a simplifying assumption, to consider the relationship between positions, we would need to incorporate additional assumptions about the functional region of the protein. As a result, we treat the positions as exchangeable and model the proportion of variant group identity (neutral, GOF, LOF) in each mutant group by a Dirichlet distribution with parameter $\hat{\varvec{\alpha }}$.

To simulate the sequencing count from the summary statistics, we use a generative model that mimics the experiment process and is completely different from the Rosace inference model for fair benchmarking. We first draw the functional score of each variant $\beta _v$ from the structure described in the summary statistics and the ones in the neutral group are set to be 0. Then, we map the functional score to its latent functional parameters: the cell growth rate in the growth screen. Next, we generate the cell count at a particular time point $N_{v,t,r}$ by the cell count at the previous time point $N_{v,t-1,r}$ and the latent functional parameters. Finally, the sequencing count is generated from a Dirichlet-Multinomial distribution with the summarized dispersion parameter and the cell count.

The simulation result shows that the simulated functional score distribution is comparable to the real experimental data (Fig. 4C). We also demonstrate that the simulation is not particularly favorable to models containing positional information such as Rosace. From Fig. 4E, we observe that in the simulation, the positional-level score is not as widespread as the real data. In addition, the positions with extreme scores (very positive scores in the OCT1 dataset) have reduced standard deviation in the real data, but not in the simulation (Additional file 1: Figs. S18d, S19d, S20d). As a result, we would expect the performance of Rosace to be better in real data than in the simulation.

### Testing Rosace false discovery control with Rosette simulation

To test the performance of Rosace, we generate simulated data using Rosette from two distinctive growth-based assays: the transporter OCT1 data where LOF variants are positively selected [10] and the kinase MET data where LOF variants are negatively selected [11]. We further included the result of a saturation genome editing dataset CARD11 [14] in Additional file 1: Figs. S17-23. The OCT1 DMS screen measures the impact of variants on cytotoxic drug SM73 uptake mediated by the transporter OCT1. If a mutation causes the transporter protein to have decreased activity, the cells in the pool will import less substrate and thus die more slowly than wide-type or those with synonymous mutations, so the LOF variants would be positively selected. In the MET DMS screen, the kinase drives proliferation and cell growth in the BA/F3 mammalian cell line in the absence of IL-3 (interleukin-3) withdrawal. If the variant protein fails to function, the cells will die faster than the wild-type cells, so the LOF variants will be negatively selected. Both data sets have a clear separation of two modes in the functional score distribution (neutral and LOF) (Additional file 1: Figs. S18a, S19a). We benchmark Rosace with *Enrich2*, *mutscan(edgeR)*, *mutscan(limma)*, and the naïve method in scenarios where we use 1 or all 3 of replicates and 1 or all 3 of selection rounds. *DiMSum* is benchmarked when there is only one round of selection because it is not designed to handle multiple rounds. Each scenario is repeated 10 times. The results of all methods show similar correlations with the latent growth rates (Additional file 1: Fig. S21), and thus, for benchmarking purposes, we focus on hypothesis testing.

**Fig. 5:**
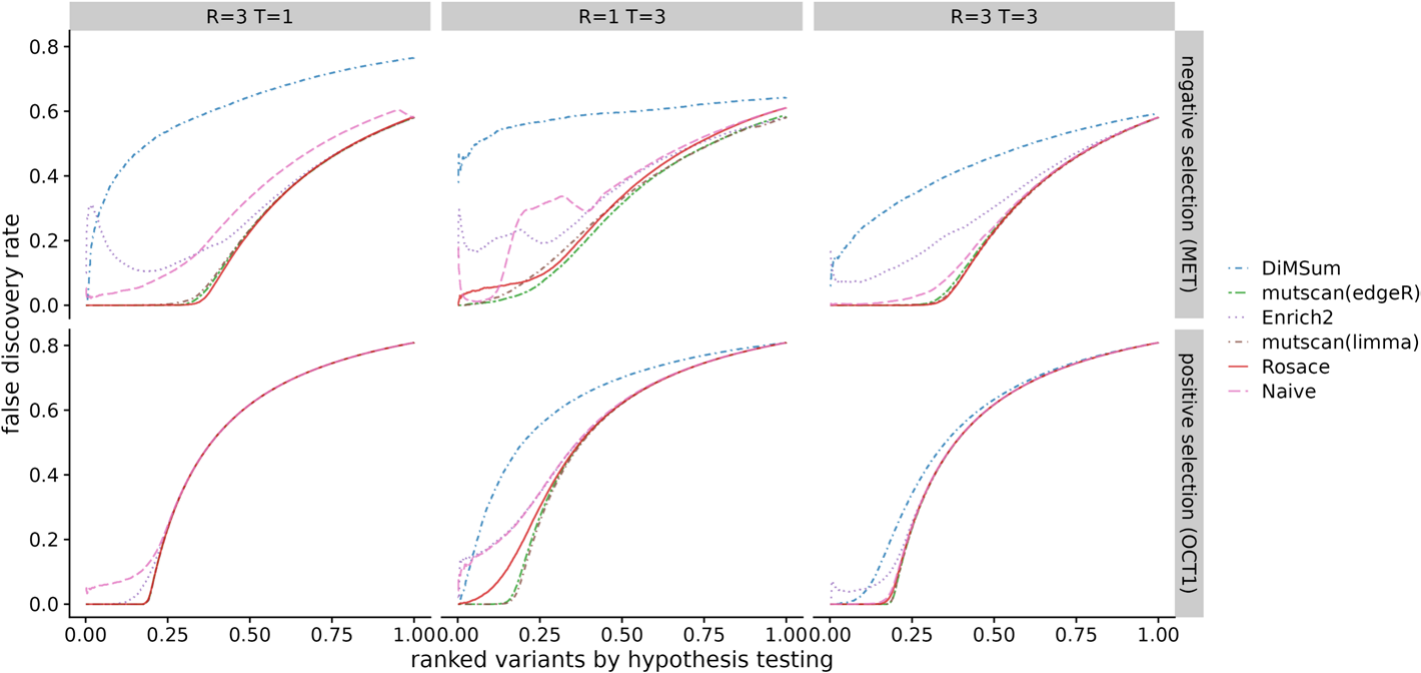
Benchmark of false discovery control on Rosette simulation. Variants are ranked by hypothesis testing (adjusted p-values or *lfsr*). The false discovery rate at each rank is computed as the proportion of neutral variants assuming all the variants till the rank cutoff are called significant. *R* is the number of replicates and *T* is the number of selection rounds. MET data is used for negative selection and OCT1 data for positive selection. Ideally, the line would be flat at 0 until the rank where all variants with true effects are discovered. (DiMSum can only process two time points and thus is disadvantaged in experiments with more than two time points, or one selection round)

We compare methods from a variant ranking point of view, comparing methods in terms of the number of false discoveries for any given number of variants selected to be LOF. This is because Rosace is a Bayesian framework that uses *lfsr* instead of *p*-values as the metric for variant selection and it is hard to translate *lfsr* to FDR for a hard threshold. Variants are ranked by adjusted *p*-values or *lfsr* (ascending). Methods that perform well will rank the truly LOF variants in the simulation ahead of non-LOF variants. In an ideal scenario with no noise, we would expect the line of ranked variants by FDR to be flat at 0 and slowly rise after all LOF variants are called. The results in Fig. 5 show that even though the position assumption is violated in the Rosette simulation, Rosace is robust enough to maintain a relatively low FDR in all simulation conditions.

### Testing Rosace power with Rosette simulation

Next, we investigate the sensitivity of benchmarking methods at different FDR or *lfsr* cutoff. It is important to keep in mind that Rosace uses raw *lfsr* from the sampling result while all other methods use the Benjamini-Hochberg Procedure to control the false discovery rate. As a result, the cutoff for Rosace is on a different scale.

**Fig. 6:**
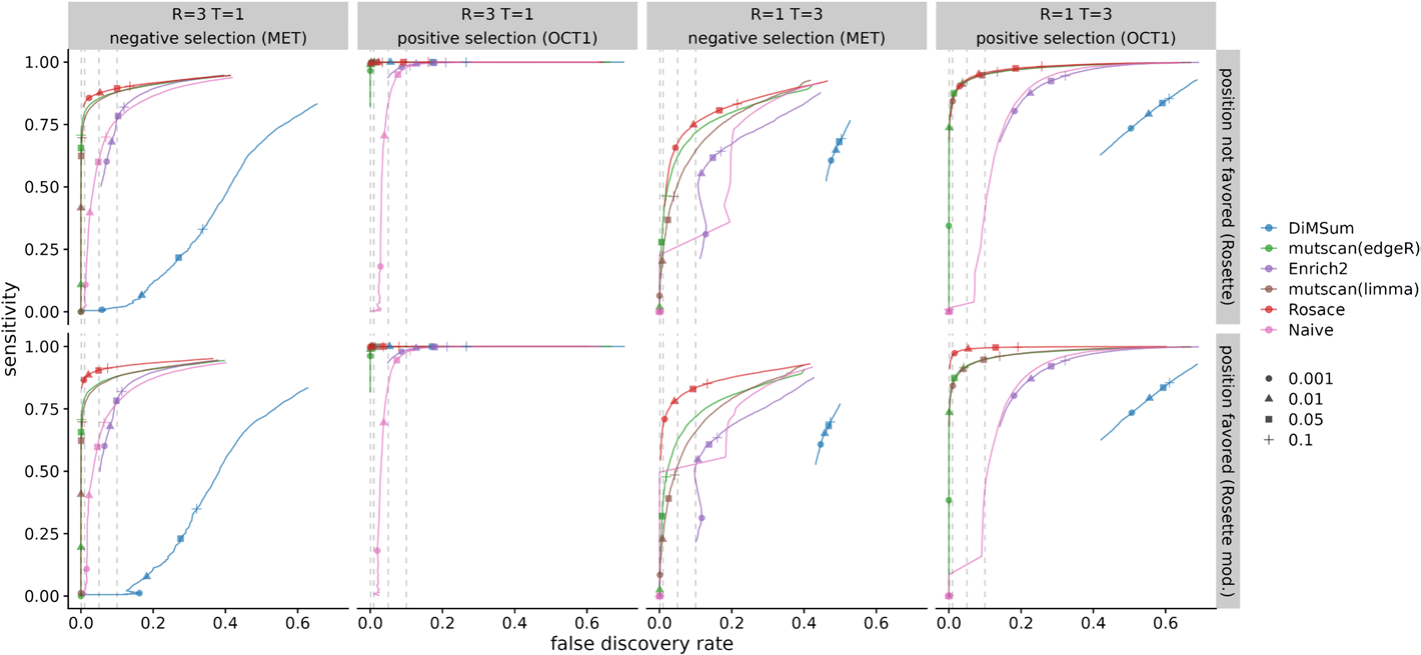
Benchmark of sensitivity versus FDR. The upper row is simulated from a modified version of Rosette simulation to favor position-informed models. The bottom row is the results from standard Rosette. Circles, triangles, squares, and crosses represent LOF variant selection at adjusted p-values or *lfsr* of 0.001, 0.01, 0.05, and 0.10, respectively. Variants with the opposite sign of selection are then excluded. Ideally, for all methods besides Rosace, each symbol would lie directly above the corresponding symbol on the x-axis indicating true FDR. For Rosace, *lfsr* has no direct translation to FDR so the cutoff represented by the shape is theoretically on a different scale. (DiMSum can only process two time points, and thus is disadvantaged in experiments with more than two time points, or one selection round)

Rosace is the only method that displays high sensitivity in all conditions with a low false discovery rate. In the case of one selection round and three replicates ($T = 1$ and $R = 3$), *mutscan(edgeR)* and *mutscan(limma)* do not have the power to detect any significant variants with the FDR threshold at 0.1. The same scenario occurs with *DiMSum* at negative selection and the naïve method at $T = 3$ and $R = 1$ (Fig. 6). The naïve method in general has very low power, while *Enrich2* has a very inflated FDR.

We benchmark Rosace on both Rosette simulations, which inherently violate the position assumption, and a modified version of Rosette that favors the position-informed model. We show that model misspecification does increase the false discovery rate of Rosace, but Rosace is robust enough to outperform all other methods (except for *DiMSum* with $T = 1$ and $R = 3$ and positive selection) even when the position assumption is strongly violated (Fig. 6).

## Discussion

One of Rosace’s contributions is accounting for positional information in DMS analysis. The model assumes the prior information that variants on the same position have similar functional effects, resulting in higher sensitivity and better FDR. Furthermore, Rosace is also capable of incorporating other types of prior information on the similarity of variants.

Despite the value of positional information in statistical inference as demonstrated in this paper, it is unclear how multiple random mutations should be position-labeled. In this case, simple position heuristics are often unsatisfying, and one might argue that a position scalar should not cluster the variants in random mutagenesis experiments with large-scale in-frame insertion and deletion, such as those on viruses. These types of experiments are not the focus of this paper, but are still very important and require careful future research.

Another critique of Rosace is the extent of bias we introduce into the score inference through position-prior information. While it is certainly possible to introduce a large bias, Rosace was developed to be a robust model ensuring near-unbiased inference or prediction even when assumptions are not precisely complied with or even violated. We demonstrate the robustness of Rosace through our data simulation framework, Rosette. The generative procedures of Rosette explicitly violate the prior assumptions made by Rosace, but even with Rosette’s data, Rosace can learn important information. We also show that the position-level shrinkage is not strong using real data, further manifesting the robustness of Rosace.

The development of DMS simulation frameworks such as Rosette can also drive experimental design. For example, to select the best number of time points and replicates with regard to the trade-off between statistical robustness and costs of the experiment, an experimentalist can conduct a pilot experiment and use its data to infer summary statistics through Rosette. Rosette will then generate simulations close to a real experiment. Experimentalists can find the optimal tool for data analysis given an experimental design by applying candidate tools to the simulation data. Similarly, given a data analysis framework, experimentalists can choose from multiple experiment designs by using Rosace to simulate all those experiments and observe if any designs have enough power to detect most of the LOF or GOF variants with a low false discovery rate.

This paper only applies our tool to growth screens, one of several functional phenotyping methods possible by DMS techniques. Another possibility is the binding experiment, where a portion of cells are selected at each time point. In this case, the expectation of functional scores computed by Rosace is a log transformation of the variant’s selection proportion [18], and one could potentially use Rosace for DMS analysis as in *Enrich2*. The third method is fluorescently activated cell sorting (FACS-seq)—a branch of literature uses binned FACS-seq screens to sort the variant libraries based on protein phenotypes. Since the experiment has multiple bins, one can potentially capture the distributional change of molecular properties beyond mean shifting [8, 10, 19, 33]. Although of different design, FACS-seq-based screens can also be analyzed using a framework similar to Rosace. Building such frameworks incorporating prior information for experiments beyond growth screens enables the community to exploit a wider range of experimental data.

As the function of a protein is rarely one-dimensional, one can measure multiple phenotypes of a variant in a set of experiments [10, 16, 34]. For example, the OCT1 data mentioned earlier [10] measures both the transporter surface expression from a FACS-seq screen and drug cytotoxicity with a growth screen. Multi-phenotype DMS experiments also call for analysis frameworks to accommodate multidimensional outcomes by modeling the interaction or the correlation of phenotypes of each variant. One successful attempt models the causal biophysical mechanism of protein folding and binding [35], and there are many more protein properties other than those two. A unifying framework for the multi-phenotype analysis remains unsolved and challenging. One needs to account for different experimental designs to directly compare scores between phenotypes, and carefully select inferred features most relevant to the scientific questions, requiring both efforts from the experimental and computational side. Nevertheless, we believe that the multi-phenotype analysis will eventually guide us to develop better mechanistic or probabilistic models for how mutations drive proteins in evolution, how they lead to malfunction and diseases, and how to better engineer new proteins.

## Conclusions

We present Rosace, a Bayesian framework for analyzing growth-based deep mutational scanning data. In addition, we develop Rosette, a simulation framework that recapitulates the properties of actual DMS experiments, but relies on an orthogonal data generation process from Rosace. From both simulation and real data analysis, we show that Rosace has better FDR control and higher sensitivity compared to existing methods and that it provides reliable estimates for downstream analyses.

## Methods

### Pipeline: raw read to sequencing count

To facilitate the broader adoption of the Rosace framework for DMS experiments, we have developed a sequencing pipeline for short-read-based experiments using Snakemake which we dub Dumpling [36]. This pipeline handles directly sequenced single-variant libraries containing synonymous, missense, nonsense, and multi-length indel mutations, going from raw reads to final scores and quality control metrics. Raw sequencing data in the form of fastq files is first obtained as demultiplexed paired-end files. The user then defines the experimental architecture using a csv file defining the conditions, replicates, and time points corresponding to each file, which is parsed along with a configuration file. The reads are processed for quality and contaminants using BBDuk, and then the paired reads are error-corrected using BBMerge. The cleaned reads are then mapped onto the reference sequence using BBMap [37]. Variants in the resulting SAM file are called and counted using the AnalyzeSaturationMutagenesis tool in GATK v4 [38]. This tool provides a direct count of the number of times each distinct genotype is detected in an experiment. We generate various QC metrics throughout the process and combine them using MultiQC for an easy-to-read final overview [39].

Due to the degeneracy of indel alignments, the genotyping of codon-level deletions sometimes does not hew to the reading frame due to leftwise alignment. Additionally, due to errors in oligo synthesis, assembly, during in vivo passaging or during sequencing, some genotypes that were not designed as part of the library may be introduced. A fundamental assumption of DMS is the independence of individual variants, and so to reduce noise and eliminate error, our pipeline removes those that were not part of our planned design before analysis, as well as renames variants to be consistent at the amino acid level, before exporting the variant counts in a format for Rosace.

### Pre-processing of sequencing count

In a growth DMS screen with *V* variants, we define *v* to be the variant index. A function *p*(*v*) maps the variant *v* to its position label. *T* indicates the number of selection rounds and index *t* is an integer ranging from 0 to *T*. A total of *R* replicates are measured, with *r* as the replicate index. We denote $c_{v,t,r}$ the raw sequencing count of cells with variant *v* at time point *t* in replicate *r*.

In addition, “mutant” refers to substitution with one of the 20 amino acids, insertion of an amino acid, or deletion. Thus, a variant is uniquely identified by its mutant and the position where the mutant occurs (*p*(*v*)).

The default pre-processing pipeline of Rosace includes four steps: variant filtering, count imputation, count normalization, and replicate integration. First, variants with more than 50% of missing count data are filtered out in each replicate. Then, variants with a few missing data (less than 50%) are imputed using either the K-nearest neighbor averaging (*K* = 10) or filled with 0. Next, imputed raw counts are log-transformed with added pseudo-count 1/2 and normalized by the wild-type cells or the sum of sequencing counts for synonymous mutations. This step, which is proposed by *Enrich2*, allows for the computed functional score of wild-type cells to be approximately 0. Additionally, the counts for each variant before selection are aligned to be 0 for simple prior specification of the intercept.

1 $$\begin{aligned} m_{v,t,r} {:=} \log \left( \frac{c_{v,t,r}+\frac{1}{2}}{c_{wt,t,r}+\frac{1}{2}} \right) - \log \left( \frac{c_{v,0,r}+\frac{1}{2}}{c_{wt,0,r}+\frac{1}{2}}\right) \end{aligned}$$

Previous papers suggest the usage of other methods such as total-count normalization when the wild-type is incorrectly estimated or subject to high levels of error [18, 20]. We include this in Rosace as an option. Finally, replicates in the same experiment are joined together for the input of the hierarchical model. If a variant is dropped out in some but not all replicates, Rosace imputes the missing replicate data with the mean of the other replicates.

### Rosace: hierarchical model and functional score inference

Rosace assumes that the aligned counts are generated by the following time-dependent linear function. Let $\beta _v$ be the defined functional score or slope, $b_v$ be the intercept, and $\epsilon _{g(v)}$ be the error term. The core of Rosace is a linear regression:

2 $$\begin{aligned} m_{v,t,r}|\beta _{v},b_v,\epsilon _{g(v)}^2 \sim \text {Normal} \left( \beta _{v} \frac{t}{T}+b_{v}, \epsilon _{g(v)}^2 \right) \end{aligned}$$

where *g*(*v*) maps the variant *v* to its mean group—the grouping method will be explained below.

*p*(*v*) is the function that maps a variant *v* to its amino acid position. If the information of variants’ mutation types is given, Rosace will assign synonymous variants to many artificial “control” positions. The number of synonymous variants per control position is determined by the maximum number of non-synonymous variants per position. Assigning synonymous variants to control positions incorporates the extra information while not giving too strong a shrinkage to synonymous variants (Additional file 1: Figs. S2-S4). In addition, we regroup positions with fewer than 10 variants together to avoid having too few variants in a position. For example, if the DMS screen has fewer than 10 mutants per position, adjacent positions will be grouped to form one position label. Also, the position of a continuous indel variant is labeled as a mutation of the leftmost amino acid residue (e.g., an insertion between positions 99 and 100 is labeled as position 99 and a deletion of positions 100 through 110 is labeled as position 100).

We assume that the variants at the same position are more likely to share similar functional effects. Thus, we build the layer above $\beta _v$ using position-level parameters $\phi _{p(v)}$ and $\sigma _{p(v)}$.

3 $$\begin{aligned} \beta _v|\phi _{p(v)},\sigma _{p(v)}^2 \sim \text {Normal}\left( \phi _{p(v)},\sigma _{p(v)}^2\right) \end{aligned}$$

The mean and precision parameters are given a weakly informative normal prior and variance parameters are given weakly informative inverse-gamma distribution.

4 $$\begin{aligned} \phi _{p(v)}\sim & {} \text {Normal}(0, 1) \nonumber \\ \sigma ^2_{p(v)}\sim & {} \textrm{InvGamma}(1,1) \end{aligned}$$

We further cluster the variant into mean groups of 25 based on its value of mean count across time points and replicates. The mapping between the variant and its mean group is denoted as *g*(*v*). Thus, we model the mean-variance relationship by assuming variants with a lower mean are expected to have higher error terms in the linear regression and vice versa.

5 $$\begin{aligned} \epsilon ^2_{g(v)} \sim \textrm{InvGamma}(1,1) \end{aligned}$$

Stan [40] is used in Rosace for Bayesian inference over our model. We use the default inference method, the No-U-Turn sampler (NUTS), a variant of the Hamiltonian Monte Carlo (HMC) algorithm. Compared to other widely used Monte Carlo samplers, for example, the Metropolis-Hastings algorithm, HMC has reduced correlation between successive samples, resulting in fewer samples reaching a similar level of accuracy [41]. NUTS further improves HMC by automatically determining the number of steps in each iteration of HMC sampling to more efficiently sample from the posterior [42].

The lower bound of the number of mutants per position index $|\{v|p(v)=i\}|$ (10) and the size of the variant’s mean group $g_p$ (25) can be changed.

### Rosette: the OCT1 and MET datasets

We use the following datasets as input of the Rosette simulation: the OCT1 dataset by Yee et al. [10] as an example of positive selection and the MET dataset by Estevam et al*.* [11] as an example of negative selection. Specifically, we use replicate 2 of the cytotoxicity selection screen in the OCT1 dataset for both score distribution and raw count dispersion. For the MET dataset, we select the experiment with IL-3 withdrawal under wild-type genetic background (without exon 14 skipping). Raw counts are extracted from replicate 1 but the scores are calculated from all three replicates because of the frequent dropouts at the initial time point.

The sequencing reads and the resulting sequencing counts are processed in the default pipeline described in the previous method sections. Scores are then computed using simple linear regression (the naïve method). The naïve method is used as the Rosette input because we are trying to learn the global distribution of the scores instead of identifying individual variants and, while uncalibrated, naïve estimates are unbiased.

### Rosette: summary statistics from real data

Summary statistics inferred by Rosette can be categorized into two types: one for the dispersion of sequencing counts and the other for the dispersion of score distribution.

First, we estimate dispersion $\eta$ in the sequencing count. We assume the sequencing count at time point 0 reflects the true variant library before selection. Since the functional scores of synonymous variants are approximately 0, the proportion of synonymous mutations in the population should approximately be the same after selection. Let the set of indices of synonymous mutations be $\textbf{v}_s = \{v_{s1}, v_{s2}, \dots \}$. The count of each synonymous mutation at time point *t* is $\textbf{c}_{\textbf{v}_s, t} = (c_{v_{s1}, t}, c_{v_{s2}, t}, \dots )$. The model we use to fit $\eta$ is thus

6 $$\begin{aligned} \textbf{c}_{\textbf{v}_s,t} \sim \text {DirMultinomial} \left( \sum \limits _{v_s \in \textbf{v}_s} c_{v_s,t},\; (\eta \Vert \textbf{v}_s\Vert ) \frac{\textbf{c}_{\textbf{v}_s,0}}{\sum \limits _{v_s \in \textbf{v}_s}\textbf{c}_{v_s,0}} \right) \end{aligned}$$

from which we find the maximum likelihood estimation $\hat{\eta }$.

Dispersion of the initial variant library $\eta _0$ is estimated similarly by fitting a Dirichlet-Multinomial distribution on the sequencing counts of the initial time point assuming that in an ideal experiment, the proportion of each variant in the library should be the same. Similar to above, the indices of all mutations are $\textbf{v} = \{1, 2, \dots , V\}$, and the count of each mutation at time point 0 is $\textbf{c}_{\textbf{v}, 0} = (c_{1, 0}, c_{2, 0}, \dots , c_{V, 0})$. From the following model

7 $$\begin{aligned} \textbf{c}_{\textbf{v}, 0} \sim \text {DirMultinomial} \left( \sum _{v \in \textbf{v}} c_{v,0}, (\eta _0 V) \frac{1}{V} \right) \end{aligned}$$

we can again find the maximum likelihood of the variant library dispersion $\hat{\eta _0}$. Notice that $\hat{\eta }_0$ is usually much smaller than $\hat{\eta }$ (i.e. more overdispersed) because $\hat{\eta }_0$ contains both the dispersion of the variant library as well as the sequencing step.

To characterize the distribution of functional scores, we first cluster mutants into groups, as mutants often have different properties and exert different influences on protein function. We calculate the empirical Jensen-Shannon divergence (JSD) to measure the distance between two mutants, using bins of 0.1 to find the empirical probability density function. Ideally, a clustering scheme should produce a grouping that reflects the inherent properties of an amino acid that are independent of position. Thus, we are more concerned with the general shape of the distribution than the similarity between paired observations. It leads to our preference for JSD over Euclidean distance as the clustering metric. To cluster mutants into four mutant groups $g_{m} = \{1, 2, 3, 4\}$, we use hierarchical clustering (“hclust” function with *complete linkage* method in R), and we record the proportions $\widehat{\varvec{p}}$ to simulate any number of mutants in the simulation (the number of mutant groups can also be changed). The underlying assumption is that mutants in each mutant group are very similar and can be treated as interchangeable. We define $f_1(v)$ as the function that maps a variant to its corresponding mutant group $g_{m}$.

Then, we cluster the variants into different variant groups. In the case of our examples, the shape is not unimodal but bimodal. The OCT1 screen has a LOF mode on the right (positive selection) and the MET screen has a LOF mode on the left (negative selection). While it is possible to observe both GOF and LOF variants, we observed in our datasets that GOF variants are so rare that they do not constitute a mode on the mixed distribution, resulting in a bimodal distribution. To cluster the non-synonymous variants into groups $g_{v}$, we use the Gaussian Mixture model with two mixtures for our examples to decide the cutoff of the groups, and we fit the Gaussian distribution for each variant group again to learn the parameters of the distribution. The synonymous variants have their own group labeled as control. Let $f_2(v)$ denote the function that maps a variant to its corresponding variant group $g_{v}$. The result of the simulation shows that even the synonymous mutations with scores close to 0 can have large negative effects due to random dropout. Thus, we later set the effect of the control and the neutral group to be constant 0 and still observe a similar distribution as seen in the real data. For each variant, we have one of the models below, depending on whether the variant results in LOF or has no effects:

8 $$\begin{aligned}{} & {} \beta _{v}|\mu _{f_2(v)},\sigma ^2_{f_2(v)} \sim \text {Normal}\left( \mu _{f_2(v)},\sigma ^2_{f_2(v)}\right) \qquad \text {(LOF)}\end{aligned}$$

9 $$\begin{aligned}{} & {} \beta _{v}|\mu _{f_2(v)},\sigma ^2_{f_2(v)} = 0 \qquad \qquad \qquad \qquad \qquad \qquad \qquad \text {(neutral)} \end{aligned}$$

We use $\widehat{\varvec{\theta }}$ to denote the collection of estimated distributional parameters for all variant groups.

Finally, we define the number of variants in each variant group at each position

10 $$\begin{aligned} o_{p,g_m,g_v} {:=} \sum _{v|\{p(v)=p\}} \mathbbm {1}_{\{f_1(v) = g_m\}} \mathbbm {1}_{\{f_2(v) = g_v\}} \end{aligned}$$

For each position *p*, we can thus find the count of variants belonging to any mutant-variant group $\varvec{o}_{p} \in \textbf{N}^{\Vert g_m \Vert \Vert g_v \Vert }$. Treating each position as an observation, we fit a Dirichlet distribution to characterize the distribution of variant group identities among mutants at any position:

11 $$\begin{aligned} \frac{\varvec{o}_{p}}{\sum \nolimits _{g_m, g_v}{o_{p,g_m,g_v}}} \sim \text {Dirichlet} (\varvec{\alpha }). \end{aligned}$$

The final summary statistics are $\hat{\eta }$, $\hat{\eta _0}$, $\hat{\varvec{p}}$, $\hat{\varvec{\theta }}$, and $\hat{\varvec{\alpha }}$. We also need *T*, the number of selection rounds, to map $\beta _v$ into the latent functional parameter $\mu _v$ in growth screens.

### Rosette: data generative model

We simulate as the real experiment the same number of mutants *M*, the number of positions *P*, and the number of variants *V* ($M \times P$). The important hyperparameters that need to be specified are the average number of reads per variant *D* (100, also referred to as the sequencing depth), initial cell population count $P_0$ (200*V*), and wild-type doubling rate $\delta$ between time points ($-2$ or 2). One also needs to specify the number of replicates *R* and selection rounds *T*.

The simulation largely consists of two major steps: (1) generating latent growth rates $\mu _v$ and (2) generating cell counts $N_{v,t,r}$ and sequencing counts $c_{v,t,r}$.

In step 1, the mutant group and variant group labeling of each variant is first generated. Specifically, we assign a mutant to the mutant group $g_m$ by the proportion $\hat{\varvec{p}}$ and then assign a variant to the variant group $g_v$ by drawing $\varvec{o}_p$ from Dirichlet distribution with parameter $\hat{\varvec{\alpha }}$ (Eq. 10). Using $\hat{\varvec{\theta }}$, we randomly generate $\beta _v$ for each variant based on its $g_v$ (Eq. 8). The mapping between $\beta _v$ and $\mu _v$ requires an understanding of the generative model, so it will be defined after we present the cell growth model.

In step 2, the starting cell population $N_{v,r,0}$ is drawn from a Dirichlet-Multinomial distribution using $\hat{\eta }_0$ and we assume that replicates are biological replicates:

12 $$\begin{aligned} N_{v,0,r} \sim \text {DirMultinomial}\left( P_0, \hat{\eta }_0\right) \end{aligned}$$

where $P_0$ is the total cell population. The cells are growing exponentially and we determine the cell count by a Poisson distribution

13 $$\begin{aligned} N_{v,t,r}|N_{v,t-1,r},\mu _v \sim \textrm{Poisson}\left( N_{v,t-1,r} \cdot e^{\mu _v \Delta t}\right) \end{aligned}$$

where $\Delta t$ is the pseudo-passing time. It differs from index *t* and will be defined in the next paragraph. Similar to how we define $\textbf{c}_{\textbf{v}, t, r}$, we define the true cell count of each variant at time point *t* and replicate *r* to be $\textbf{N}_{\textbf{v}, t, r} = (N_{1, t, r}, \dots , N_{V, t, r})$. The sequencing count for each variant is

14 $$\begin{aligned} \textbf{c}_{\textbf{v},t,r}|\textbf{N}_{\textbf{v},t,r} \sim \text {DirMultinomial} \left( DV,\; \hat{\eta }\frac{\textbf{N}_{\textbf{v},t,r}}{\sum \limits _{1 \le v \le V}N_{v,t,r}} \right) \end{aligned}$$

where *D* is the sequencing depth per variant. Empirically, we can set input $\hat{\eta }$ and $\hat{\eta }_0$ slightly higher than the estimated summary statistics. This is because the estimated values encompass all the noises in the experiment, while the true values only represent the noise from the sequencing step.

To find the mapping between $\beta _v$ and $\mu _v$, we define $\delta$ to be the wild-type doubling rate and naturally compute $\Delta t:= \frac{\delta \log 2}{\mu _{wt}}$, the pseudo-passing time in each round. Then we can compute the expectation of $\beta _v$ with the linear regression model. For simplicity, we omit the replicate index *r* and assume *r* is fixed in the next set of equations.

15 $$\begin{aligned} \mathbb {E}(N_{v,t}|\mu _v, N_{v,0})&= \mathbb {E}(N_{v, t-1}|\mu _v, N_{v,0})\exp \left( \mu _v\Delta t\right) \\&= \mathbb {E}(N_{v, t-1}|\mu _v, N_{v,0})2^{\delta \frac{\mu _v}{\mu _{wt}}} \\&= N_{v,0} 2^{t\delta \frac{\mu _v}{\mu _{wt}}}\\ \log \left( \frac{\mathbb {E}(N_{v,t}|\mu _v, N_{v,0})}{\mathbb {E}(N_{wt,t}|\mu _{wt}, N_{wt,0})}\right)&- \log \left( \frac{N_{v,0}}{N_{wt,0}}\right) = (\log 2)\frac{t\delta }{\mu _{wt}}(\mu _v - \mu _{wt}) \\ \mathbb {E}(m_{v,t})&= t\left( (\log 2)\frac{\delta }{\mu _{wt}}(\mu _v - \mu _{wt})\right) \\ \mathbb {E}(\beta _v)&= (\log 2)\frac{\delta T}{\mu _{wt}}(\mu _v - \mu _{wt}) \end{aligned}$$

The final mapping between simulated $\beta _v$ and $\mu _v$ is then described in the following

16 $$\begin{aligned} \mu _v {:=} \mu _{wt}\left( \frac{\beta _v}{\delta T\log 2 }+1\right) \end{aligned}$$

with $\mu _{wt}$ set to be $\text {sgn}(\delta )$.

### Modified Rosette that favors position-informed models

In the original, position-agnostic version of Rosette, a $\Vert g_m \Vert \Vert g_v \Vert$-dimensional vector is drawn from the same Dirichlet distribution for each position. The vector can be regarded as a quota for each mutant-variant group. Variants at each position are assigned their mutant-variant group according to the quota. As a result, at one position, variants from all variant groups (neutral, LOF, and GOF) would exist, and this violates the assumption in Rosace that variants at one position would have similar functional effects (strong LOF and GOF variants are very unlikely to be at the same position). To show that Rosace could indeed take advantage of the position information when it exists in the data, we create a modified version of Rosette where variants at one position could only belong to one variant group. Specifically, a position can have either neutral, LOF, or GOF variants, but not a mixture among any variant groups.

### Benchmarking

The naïve method (simple linear regression) is conducted by the “lm” function in R on processed data. For each variant, normalized counts are regressed against time. Raw two-sided *p*-values are computed from *t*-statistics given by the “lm” function. It is then corrected using the Benjamini-Hochberg Procedure to adjust the *p*-values.

For *Enrich2*, we use the built-in variant filtering and wild-type (“wt”) normalization. All analyses use a random-effect model as presented in the paper. When there is more than one selection round, we use weighted linear regression. Otherwise, a simple ratio test is performed. The resulting *p*-values are adjusted using the Benjamini-Hochberg Procedure.

*DiMSum* requires the variant labeling to be DNA sequences. As a result, we have to generate dummy sequences. It is applied to all simulations with one selection round with the default settings. The *z*-statistics are computed using the variant’s mean estimate over the estimated standard deviation and the adjusted *p*-value is computed from the *z*-score with Benjamini-Hochberg procedure. *DiMSum* only processes data with one selection round (two time points) and thus may be disadvantaged when analyzing datasets with multiple selection rounds.

*mutscan* is an end-to-end pipeline that requires the input to be sequencing reads. Conversely, Rosette only generates sequencing counts, which can be calculated from sequencing reads but cannot be used to recover sequencing reads. To facilitate benchmarking, we use a *SummarizedExperiment* object to feed the Rosette output to their function “calculateRelativeFC,” which does take sequencing counts as input. We benchmark both *mutscan(edgeR)* and *mutscan(limma)* with default normalization and hyperparameters as provided in the function. We use the “logFC_shrunk” and “FDR” columns in *mutscan(edgeR)* output and the “logFC” and “adj.P.Val” columns in *mutscan(limma)* output.

We run Rosace with position information of variants and labeling of synonymous mutations. However, Rosace is a Bayesian framework so it does not compute FDR like the frequentist methods above. All Rosace power/FDR calculations are done under the Bayesian local false sign rate (*lfsr*) setting [28]. As a result, in the simulation, we present the rank-FDR curve and the FDR-Sensitivity curve as the metrics instead of setting an identical or different hard threshold on FDR and *lfsr*. In the real data benchmarking, both the FDR and *lfsr* thresholds are set to be 0.05.

Rosace without position label is denoted as Rosace (nopos) in the Additional file 1: Figs. S5–S15, S19–S23, and S25. It removes the position layer in Fig. 2C and keeps only the variant and replicate layer. The test statistics and model evaluation are presented identically as the full Rosace model.

### Supplementary information

Additional file 1: Supplementary figures and tables.

Additional file 2: Review history.

## Data Availability

Rosace is implemented as an R package and is distributed on GitHub (https://github.com/pimentellab/rosace), under the MIT open-source license. The package also includes functions for Rosette simulation. An archived version of Rosace is available on Zenodo [43]. The integrated sequencing pipeline for short-read-based experiments is available on GitHub (https://github.com/odcambc/dumpling). Scripts and pre-processed public datasets used to perform data analysis and generate figures for the paper are uploaded on GitHub as well (https://github.com/roserao/rosace-paper-script). The protein datasets we used are as follows: OCT1 [10], MET [11], CARD11 [14], MSH2 [12], BRCA1 [13], BRCA1-RING [23], and Cohesin [29]. OCT1 and MET are available on NIH NCBI BioProject with accession codes PRJNA980726 and PRJNA993160. CARD11, BRCA1, and Cohesin are available as supplementary files to their respective publications. MSH2 is available on Gene Expression Omnibus with accession code GSE162130. BRCA1-RING is available on MaveDB with accession code mavedb:00000003-a-1. The benchmarking datasets are EVE [31] (evemodel.org), ClinVar [30] (gnomad.broadinstitute.org), and AlphaMissense [32] (alphamissense.hegelab.org).
