# Supplementary material for: Rosace: a robust deep mutational scanning analysis framework employing position and mean-variance shrinkage
Source: Genome Biol. 2024 May 24;25:138. doi: 10.1186/s13059-024-03279-7 (PMC11127319; doi:10.1186/s13059-024-03279-7)
Supplement: Supplementary file 1 — Additional file 1: Supplementary figures and tables. [file 13059_2024_3279_MOESM1_ESM.pdf]

**Target**	**Assay**	**Media**	**LOF variants effect sign**	**Replicates**	**Time Points**	**Variants**	**Reference**
OCT1	SM73 drug cytoxicity	Human HEK293T cell line	Positive	3	4	11305	Yee et al. [2023]
MET	Kinase driven proliferation	Murine Ba/F3 cell line	Negative	3	4	5562	Estevam et al. [2023]
CARD11	Immune cell signaling	Human TMD8 cell line	Negative	5	2	2699	Meitlis et al. [2020]
MSH2	6-TG sensitivity	Human HAP1 cell line	Positive	3	2	19222	Jia et al. [2021]
BRCA1	gRNA abundance	Human HAP1 cell line	Negative	2	3	2230	Findlay et al. [2018]
BRCA1-RING	E3 ubiquitin ligase activity	Phage	Negative	6	6	3714	Starita et al. [2015]
Cohesin	Cohesin binding affinity	*Clostridium cellulolyticum*	Negative	1	2	3144	Kowalsky and Whitehead [2016]

Table S1: Overview of proteins used as analysis targets.

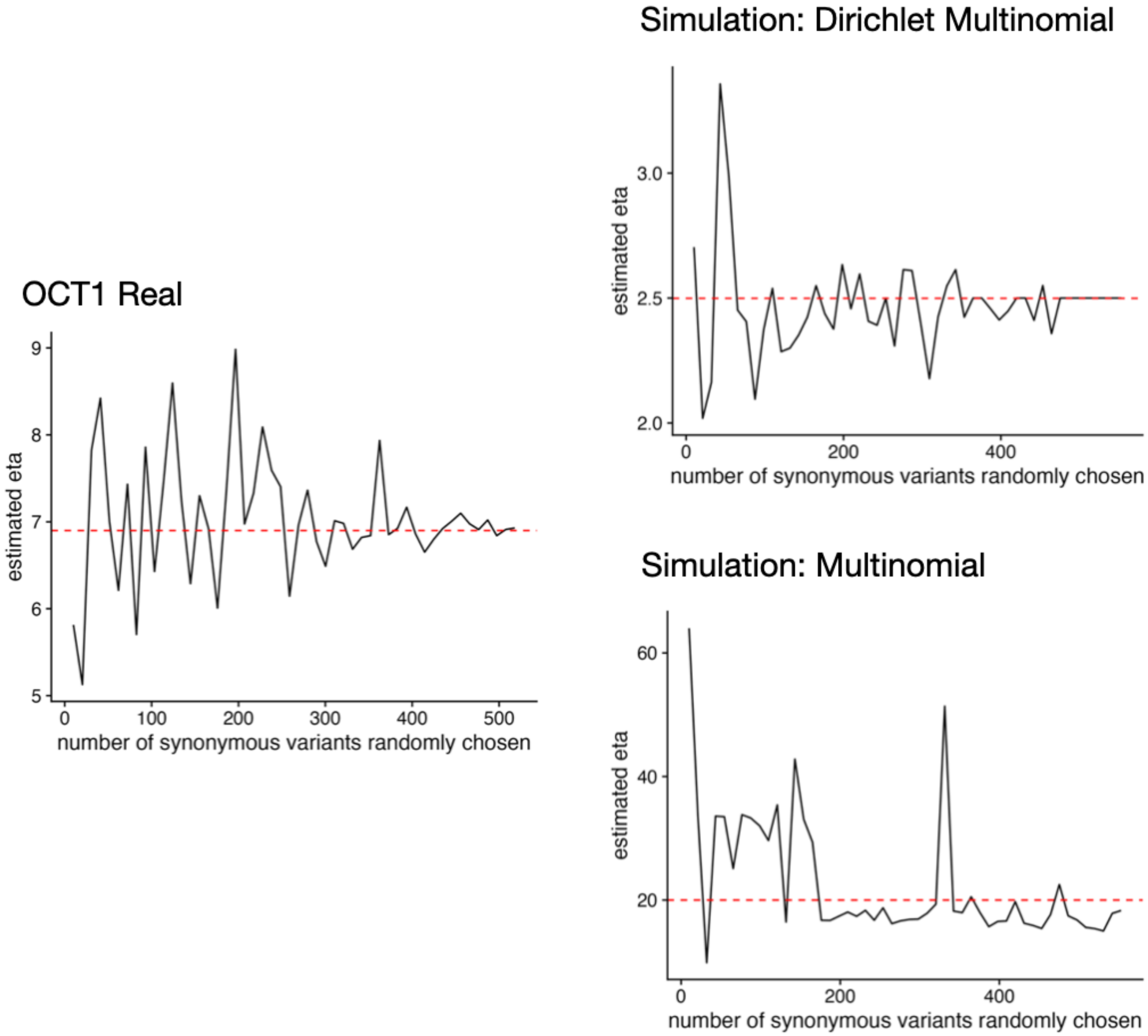

Fig. S1: **Dispersion of the sequencing counts.** The multinomial distribution is not enough to characterize the large variability of proportion changes within the synonymous mutations, so instead we use the Dirichlet-Multinomial distribution to adjust for the overdispersion in the simulation. The y-axis is the estimated dispersion value and the x-axis is the number of synonymous randomly chosen as input to complete the estimation.

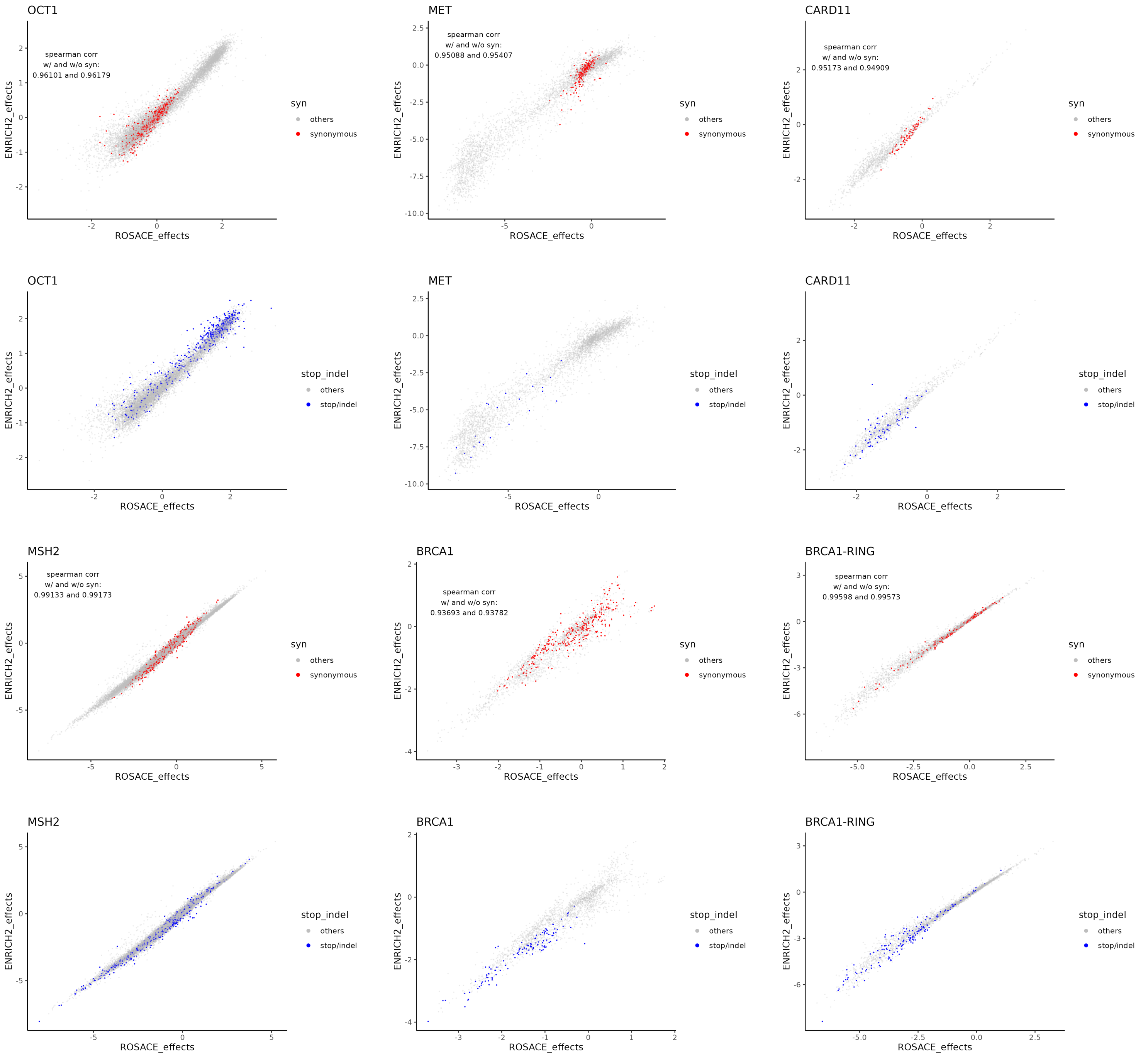

Fig. S2: **Correlation between the estimated effects of `Rosace` and *Enrich2*.** `Rosace` assigns synonymous variants to a number of artificial control positions but does not excessively shrink their effects. Incorporating positional information does not excessively shrink nonsense and indel variants to their positional mean either. We also calculated the Spearman correlation between scores and found that the inclusion of synonymous does not significantly change the correlation.

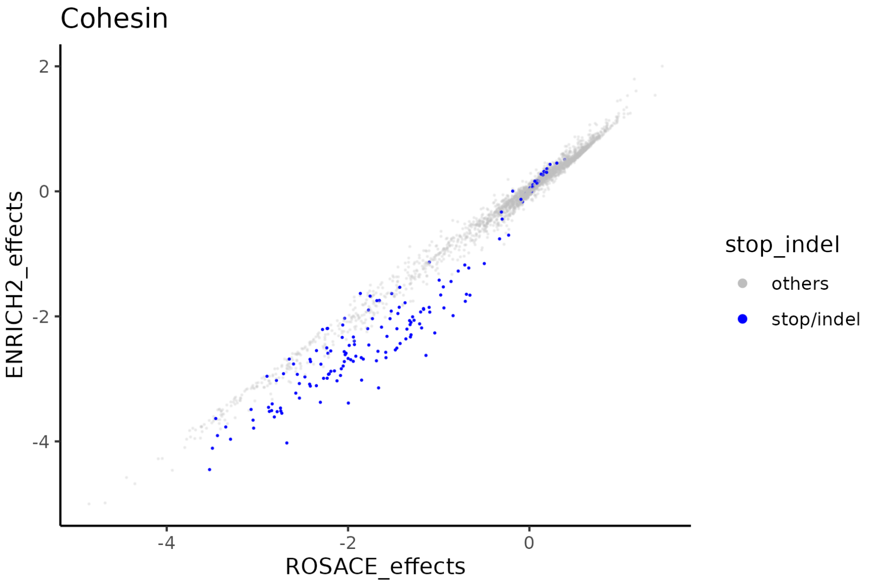

Fig. S3: **Correlation between the estimated effects of `Rosace` and *Enrich2*.** Cohesin is a bacterial protein and we replicate the analyses in Fig. S2. Note that the Cohesin dataset does not contain synonymous variants. Incorporating positional information does not excessively shrink nonsense variants to their positional mean either.

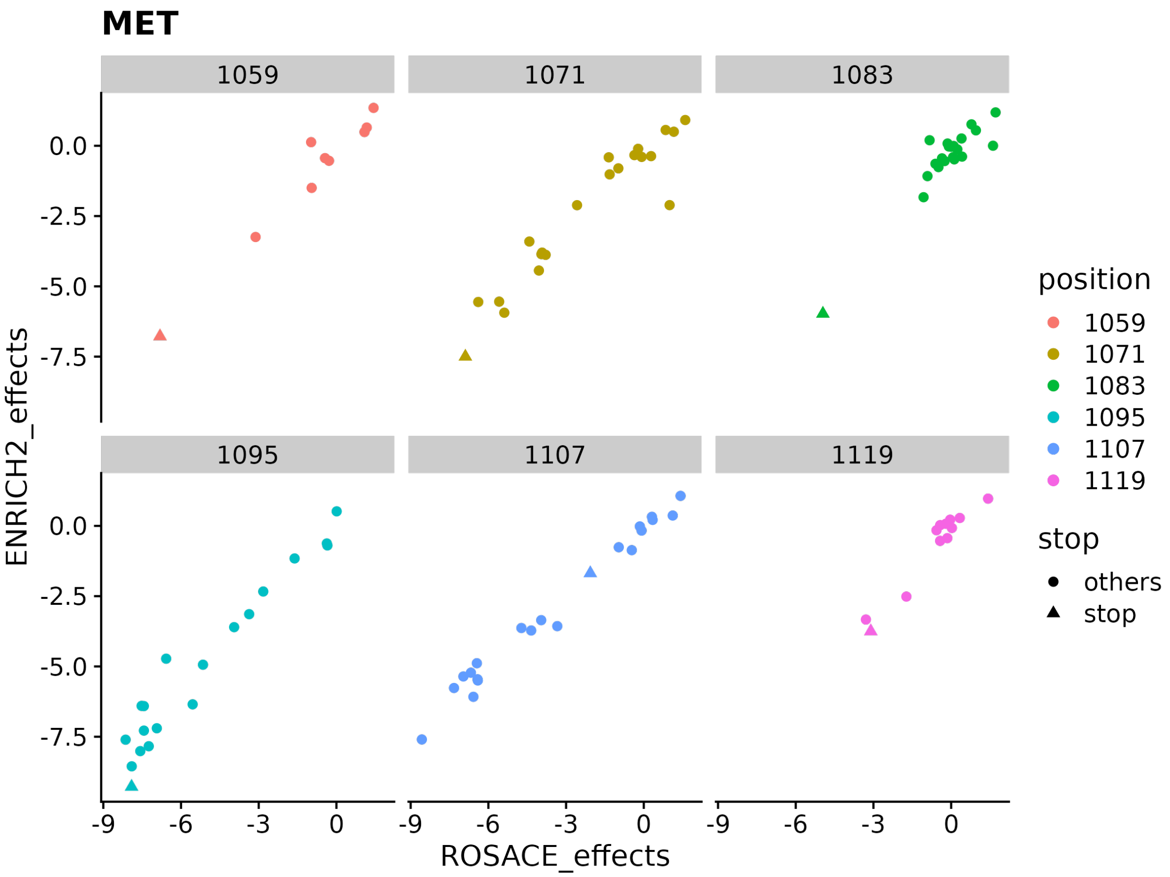

Fig. S4: **Correlation between the estimated effects of `Rosace` and Enrich2, example positions.** Nonsense variants (triangles) can escape the positional mean.

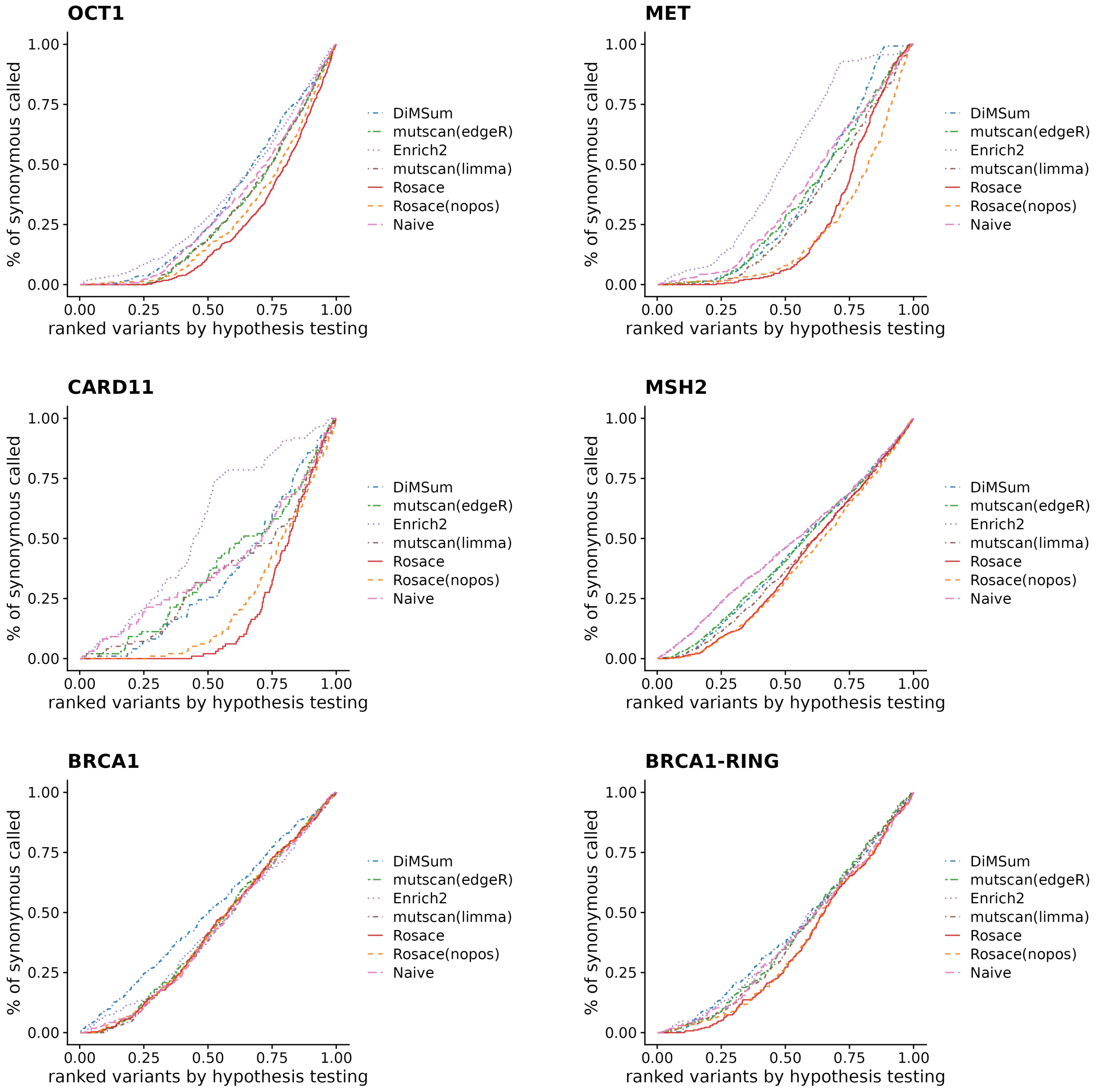

Fig. S5: **Variant rank cutoff plotted against false positive rate.** Ranks are calculated using test statistics. The false positive rate is proxied by the ratio of synonymous variants called. Cohesin does not have synonymous variants.
(DiMSum can only process two time points, and thus is disadvantaged in experiments with more than two time points, i.e., OCT1, MET, BRCA1, and BRCA1-RING.)

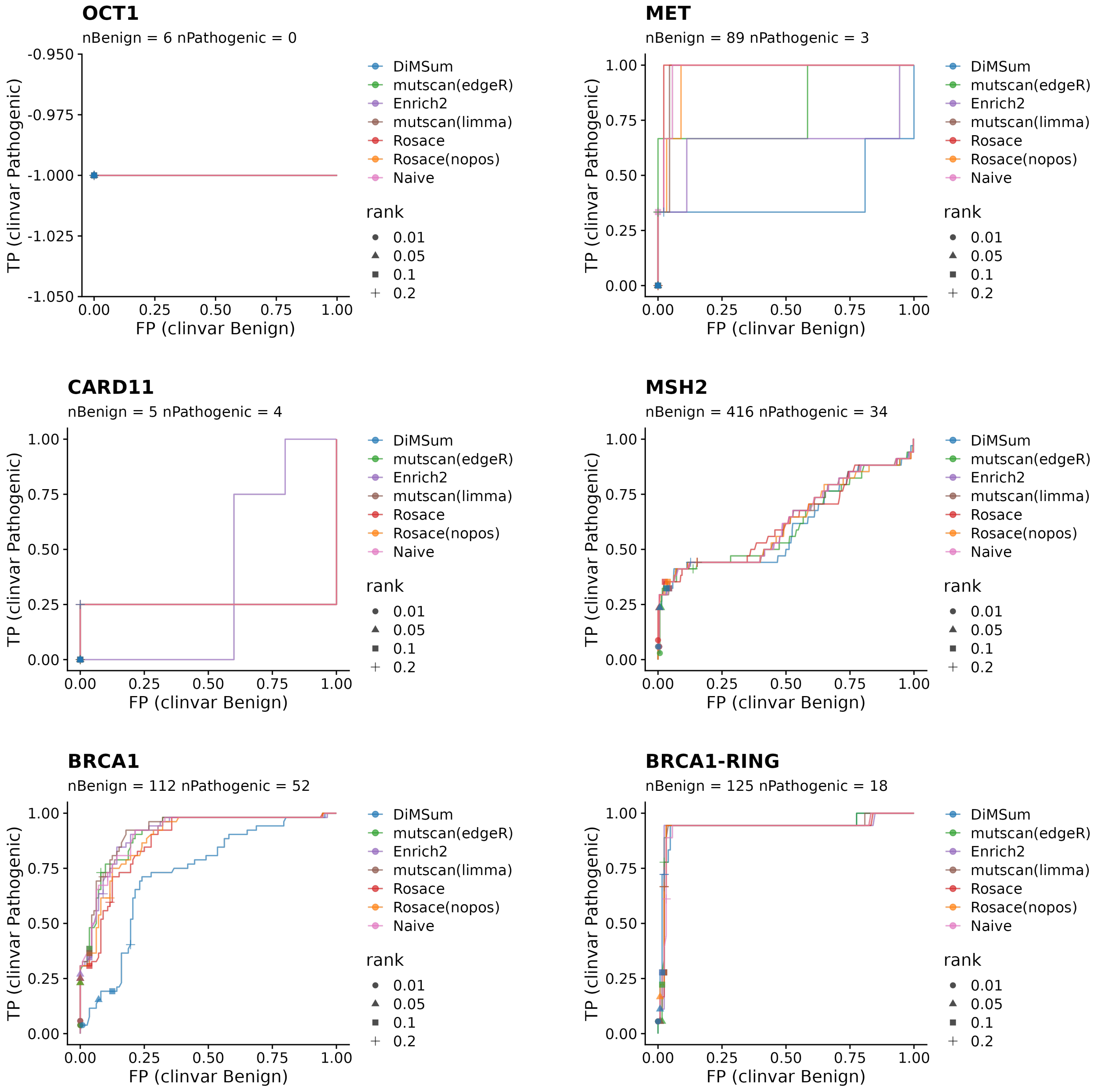

Fig. S6: **Receiver operating characteristic (ROC) curve using ClinVar and effect sizes.** Ranks are calculated using effect sizes. The true and false positive rates are calculated using the pathogenic (Pathogenic, Likely Pathogenic) and benign variants (Benign, Likely Benign) identified by ClinVar respectively Landrum et al. [2018]. Cohesin is not labelled by ClinVar. (DiMSum can only process two time points, and thus is disadvantaged in experiments with more than two time points, i.e., OCT1, MET, BRCA1, and BRCA1-RING.)

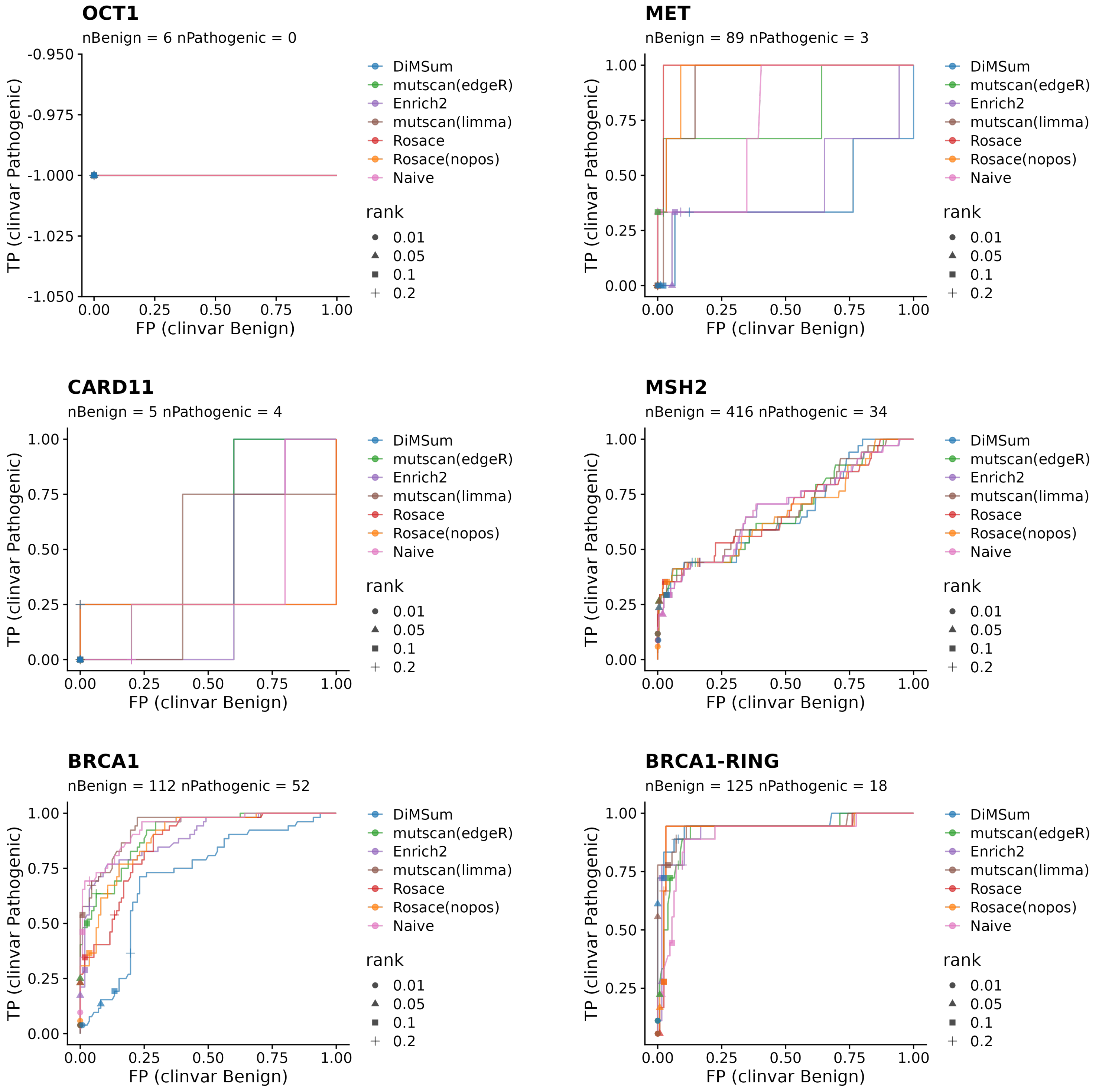

Fig. S7: **Receiver operating characteristic (ROC) curve using ClinVar and test statistics.** Ranks are calculated using test statistics. The true and false positive rates are calculated using the pathogenic (Pathogenic, Likely Pathogenic) and benign (Benign, Likely Benign) variants identified by ClinVar respectively Landrum et al. [2018]. Cohesin is not labelled by ClinVar. (DiMSum can only process two time points, and thus is disadvantaged in experiments with more than two time points, i.e., OCT1, MET, BRCA1, and BRCA1-RING.)

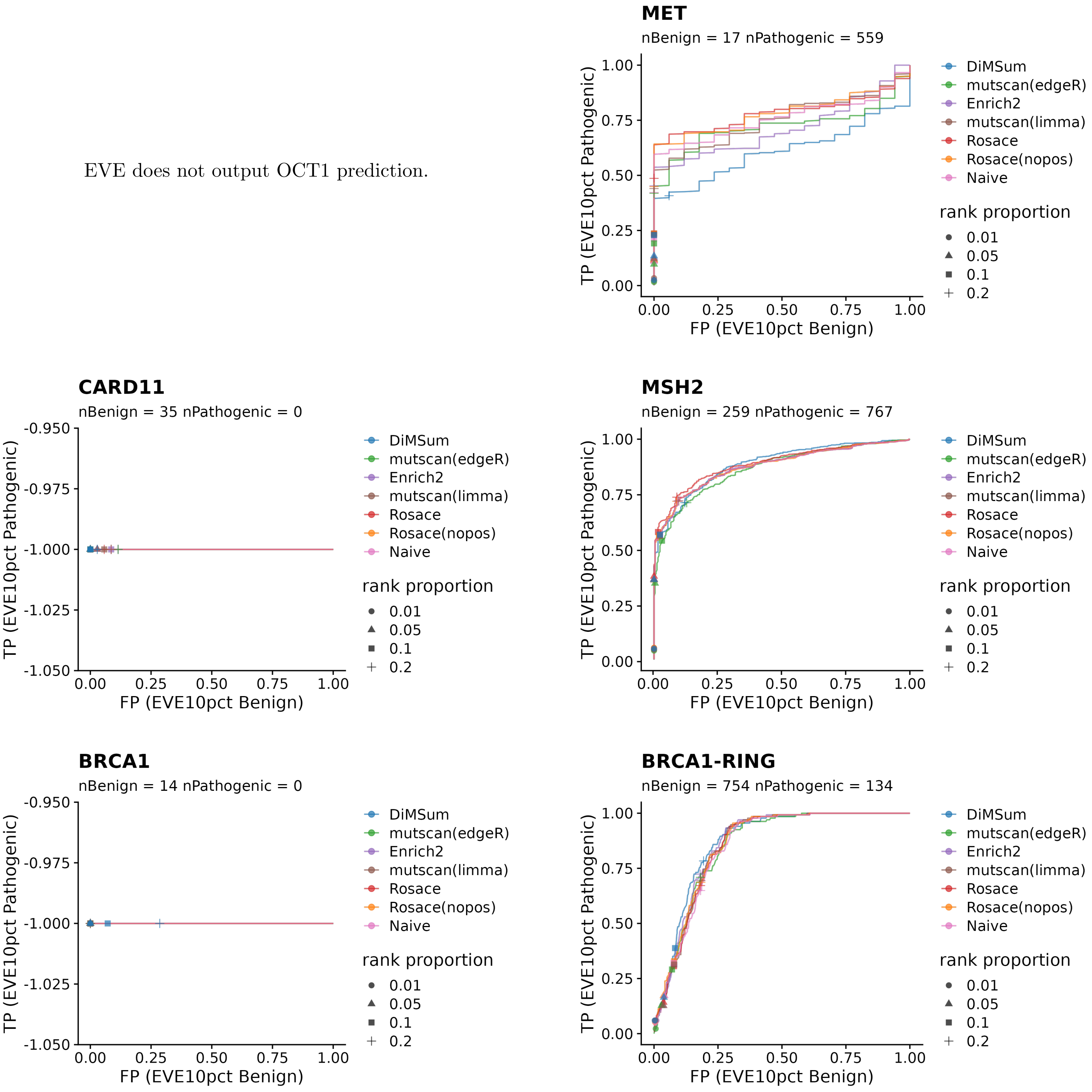

Fig. S8: **Receiver operating characteristic (ROC) curve using EVE 10% and effect sizes.** Ranks are calculated using effect sizes. The true and false positive rates are calculated using the pathogenic and benign variants identified by EVE 10% respectively Frazer et al. [2021]. EVE does not provide predictions for OCT1 or Cohesin.
(DiMSum can only process two time points, and thus is disadvantaged in experiments with more than two time points, i.e., OCT1, MET, BRCA1, and BRCA1-RING.)

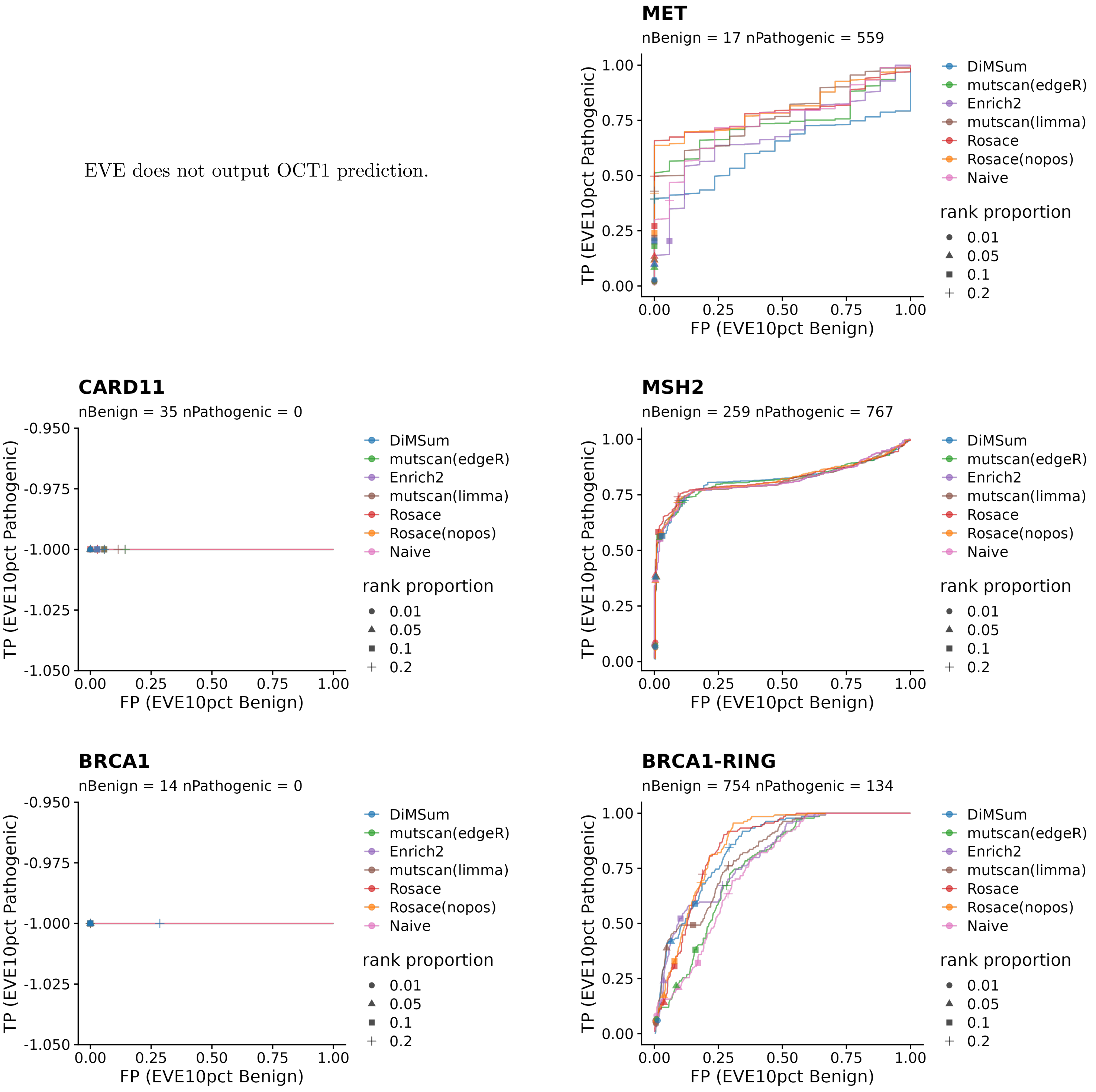

Fig. S9: **Receiver operating characteristic (ROC) curve using EVE 10% and test statistics.** Ranks are calculated using test statistics. The true and false positive rates are calculated using the pathogenic and benign variants identified by EVE 10% respectively Frazer et al. [2021]. EVE does not provide predictions for OCT1 or Cohesin.
(DiMSum can only process two time points, and thus is disadvantaged in experiments with more than two time points, i.e., OCT1, MET, BRCA1, and BRCA1-RING.)

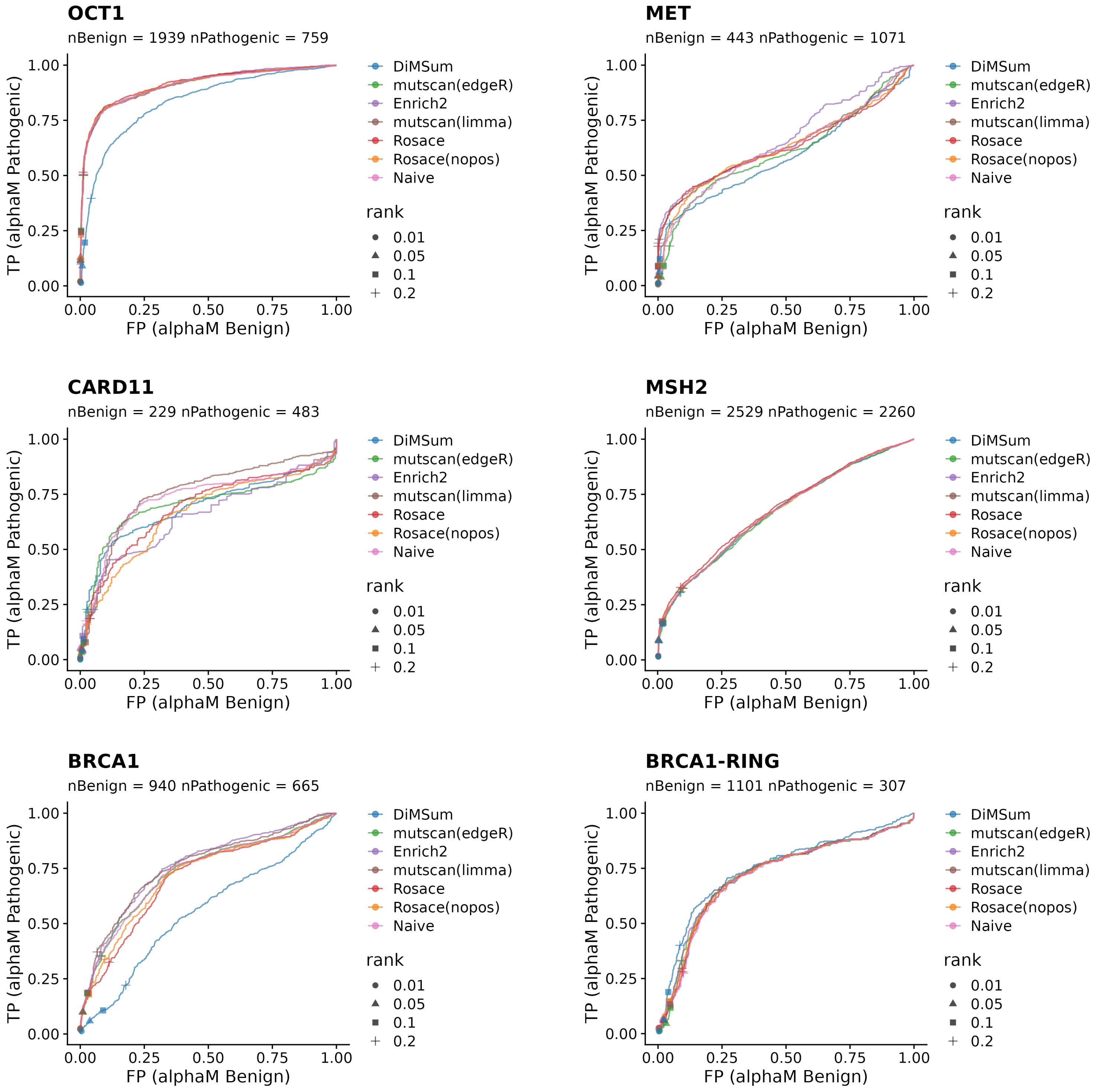

Fig. S10: **Receiver operating characteristic (ROC) curve using AlphaMissense and effect sizes.** Ranks are calculated using effect sizes. The true and false positive rates are calculated using the likely pathogenic and likely benign variants identified by AlphaMissense respectively Cheng et al. [2023]. Cohesin is not labelled by AlphaMissense.
(DiMSum can only process two time points, and thus is disadvantaged in experiments with more than two time points, i.e., OCT1, MET, BRCA1, and BRCA1-RING.)

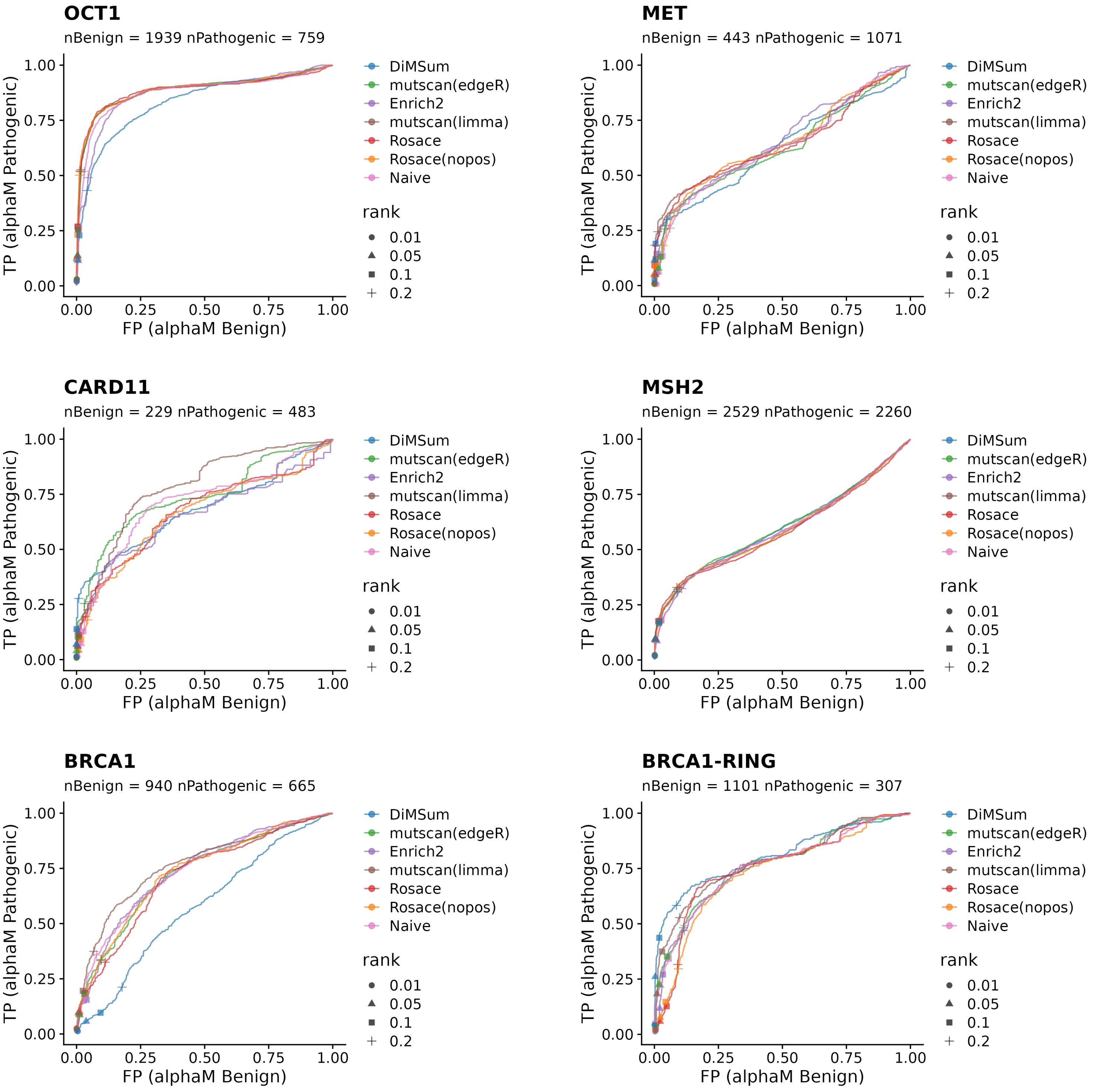

Fig. S11: **Receiver operating characteristic (ROC) curve using AlphaMissense and test statistics.** Ranks are calculated using test statistics. The true and false positive rates are calculated using the likely pathogenic and likely benign variants identified by AlphaMissense respectively Cheng et al. [2023]. Cohesin is not labelled by AlphaMissense.
(DiMSum can only process two time points, and thus is disadvantaged in experiments with more than two time points, i.e., OCT1, MET, BRCA1, and BRCA1-RING.)

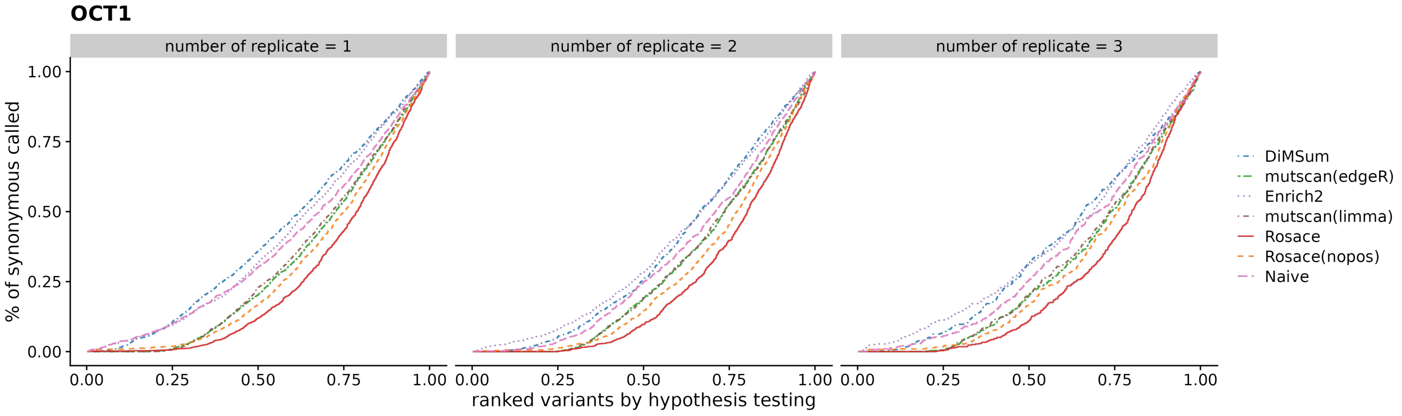

(a)

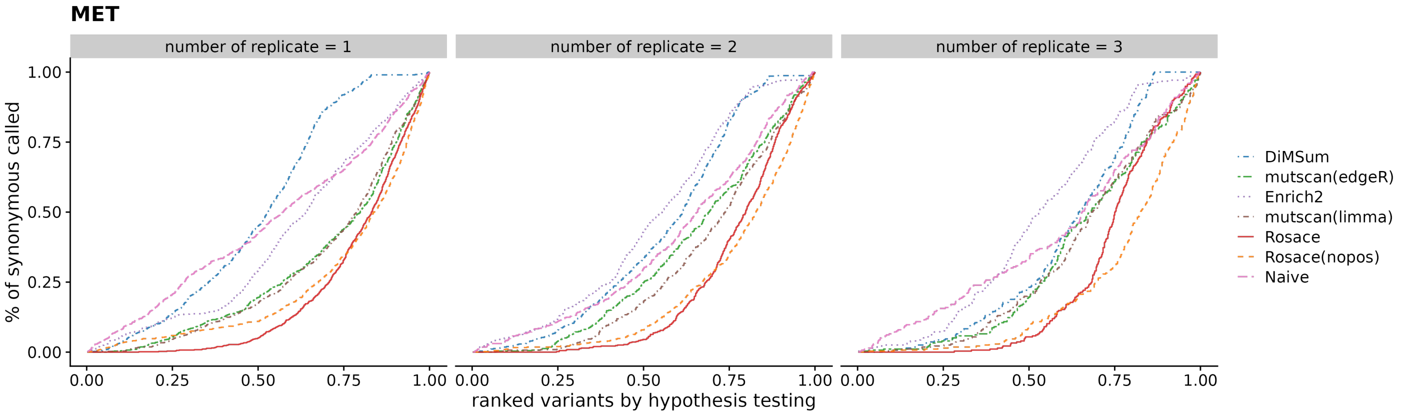

(b)

Fig. S12: **Variant rank cutoff plotted against false positive rate.** Analyses are performed using 1, 2, and 3 replicates respectively to showcase the performance of analysis tools using different numbers of replicates. Cohesin does not have synonymous variants.

(DiMSum can only process two time points, and thus is disadvantaged in experiments with more than two time points, i.e., OCT1, MET, BRCA1, and BRCA1-RING.)

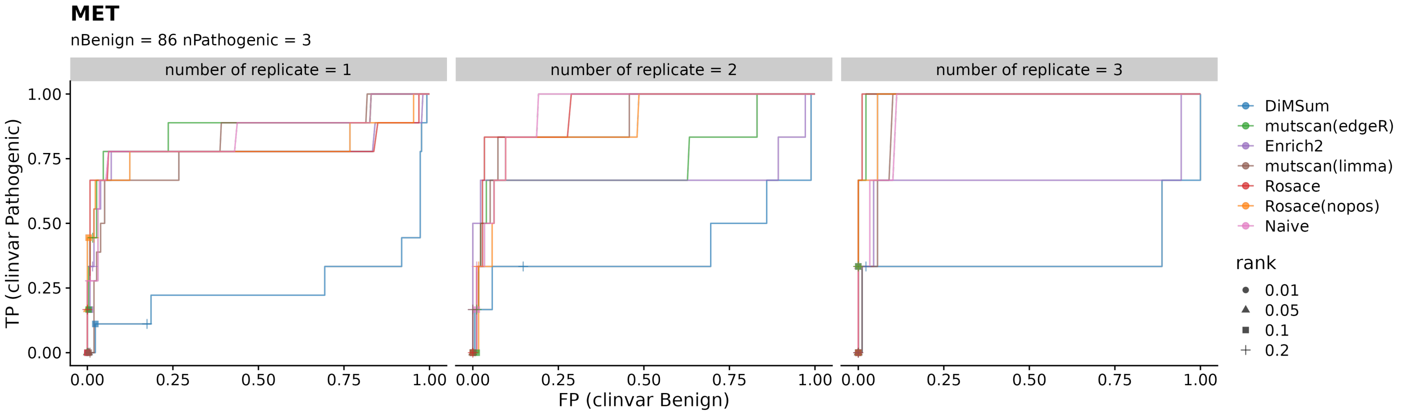

(a)

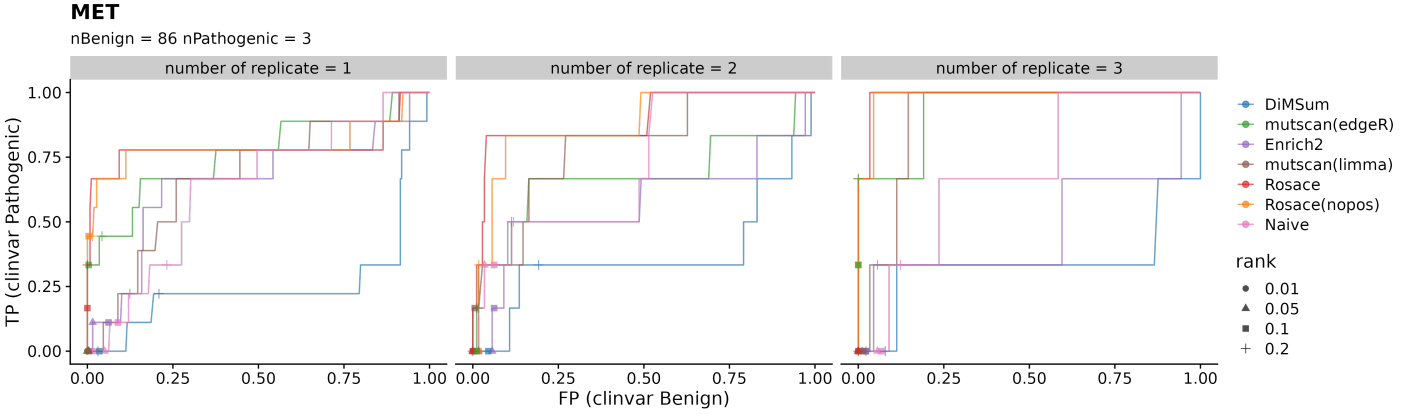

(b)

Fig. S13: **Receiver operating characteristic (ROC) curve using ClinVar.** The true and false positive rates are calculated using the pathogenic and benign variants identified by ClinVar respectively Landrum et al. [2018]. ClinVar does not provide pathogenic variants of OCT1. Analyses are performed using 1, 2, and 3 replicates respectively to showcase the performance of analysis tools using different numbers of replicates. Fig. S13a calculates rank using effect sizes. Fig. S13b calculates rank using test statistics.
(DiMSum can only process two time points, and thus is disadvantaged in experiments with more than two time points, i.e., OCT1, MET, BRCA1, and BRCA1-RING.)

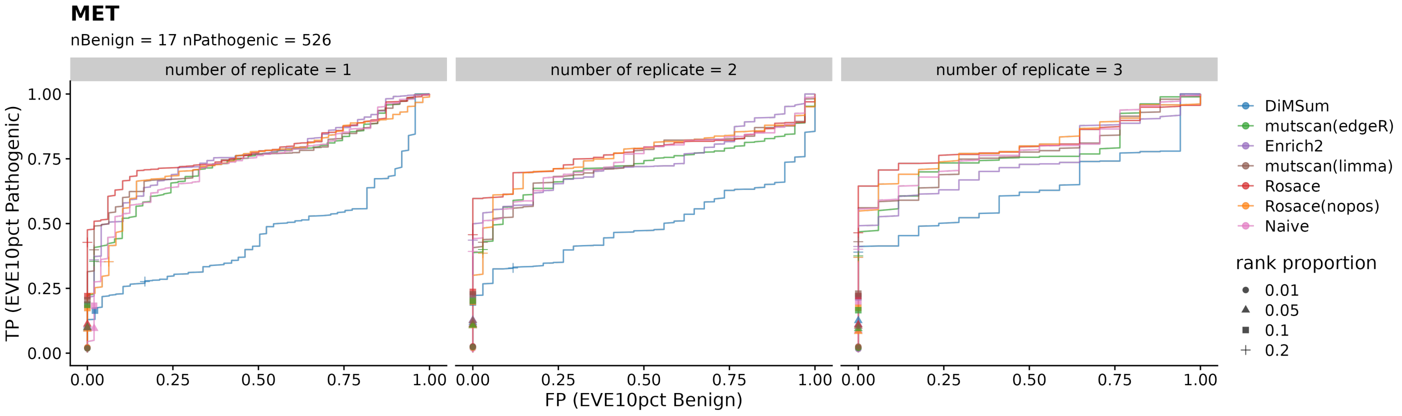

(a)

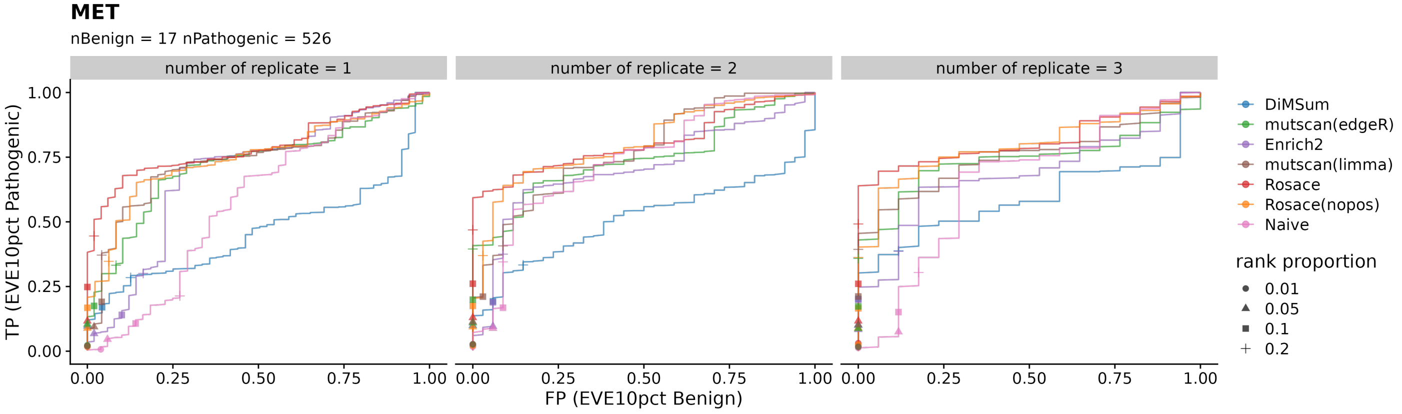

(b)

Fig. S14: **Receiver operating characteristic (ROC) curve using EVE10%.** The true and false positive rates are calculated using the pathogenic and benign variants identified by EVE 10% respectively Frazer et al. [2021]. EVE does not provide predictions for OCT1. Analyses are performed using 1, 2, and 3 replicates respectively to showcase the performance of analysis tools using different numbers of replicates. Fig. S14a calculates rank using effect sizes. Fig. S14b calculates rank using test statistics.
(DiMSum can only process two time points, and thus is disadvantaged in experiments with more than two time points, i.e., OCT1, MET, BRCA1, and BRCA1-RING.)

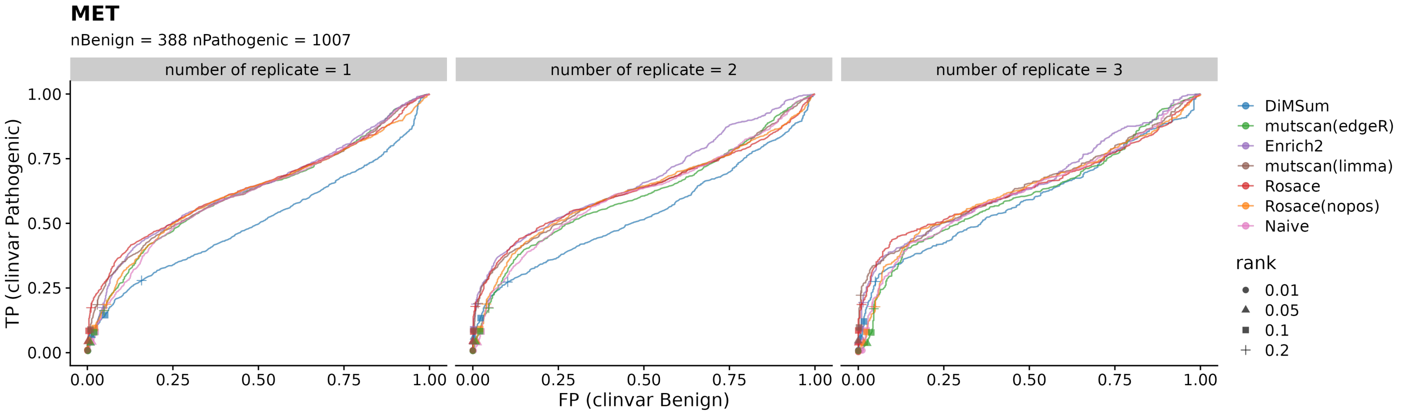

(a)

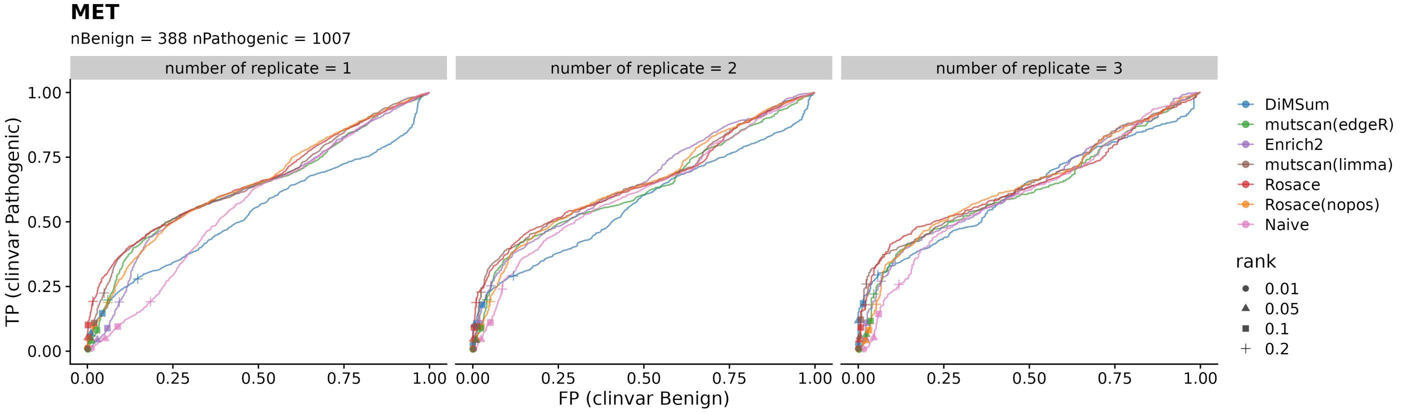

(b)

Fig. S15: **Receiver operating characteristic (ROC) curve using AlphaMissense.** The true and false positive rates are calculated using the likely pathogenic and likely benign variants identified by AlphaMissense respectively Cheng et al. [2023]. Analyses are performed using 1, 2, and 3 replicates respectively to showcase the performance of analysis tools using different numbers of replicates. Fig. S15a calculates rank using effect sizes. Fig. S15b calculates rank using test statistics.
(DiMSum can only process two time points, and thus is disadvantaged in experiments with more than two time points, i.e., OCT1, MET, BRCA1, and BRCA1-RING.)

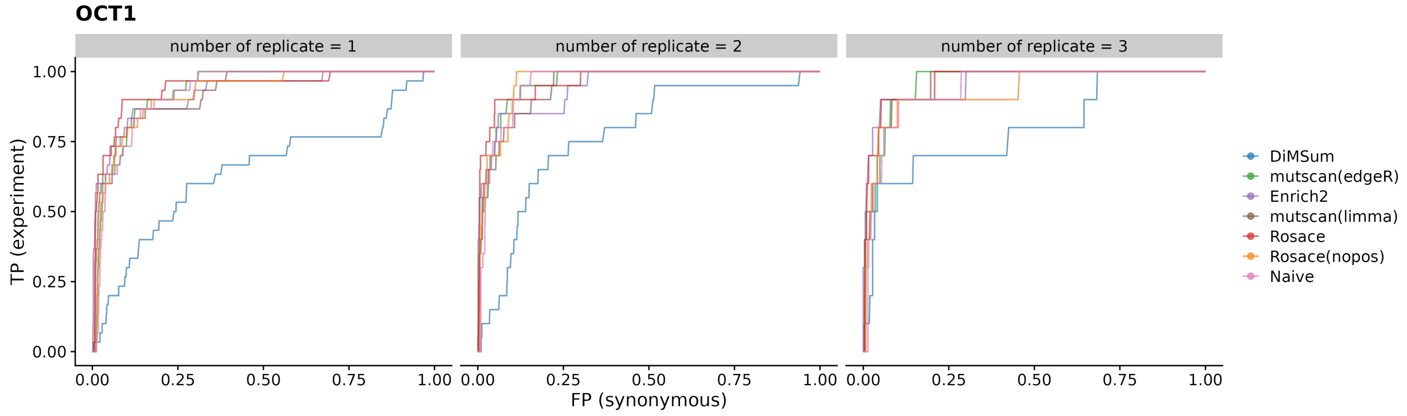

(a)

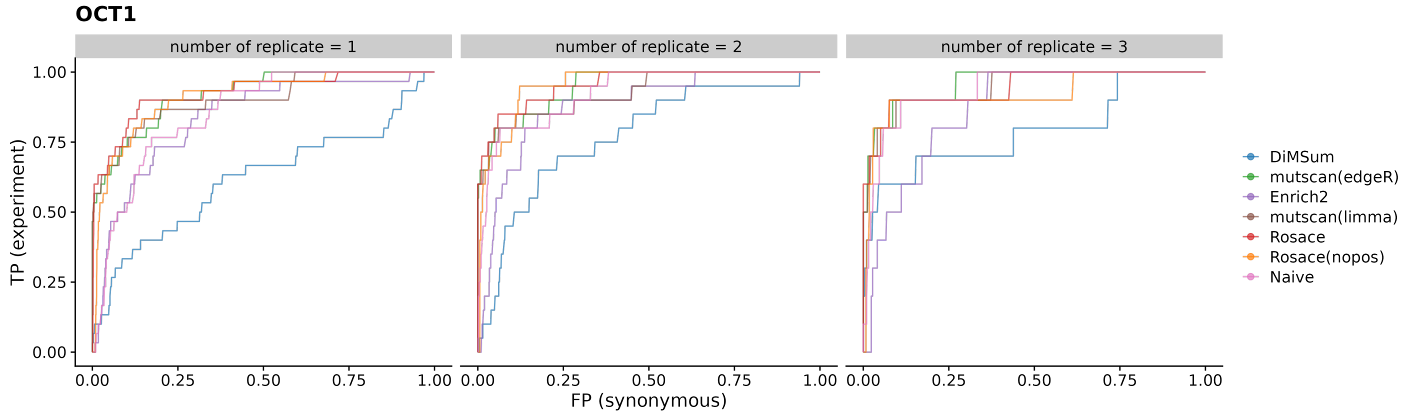

(b)

Fig. S16: **Receiver operating characteristic (ROC) curve using the OCT1 paper and the synonymous mutations.** The true and false positive rates are calculated using the original OCT1 paper. Analyses are performed using 1, 2, and 3 replicates respectively to showcase the performance of analysis tools using different numbers of replicates. Fig. S16a calculates rank using effect sizes. Fig. S16b calculates rank using test statistics.

(DiMSum can only process two time points, and thus is disadvantaged in experiments with more than two time points, i.e., OCT1, MET, BRCA1, and BRCA1-RING.)

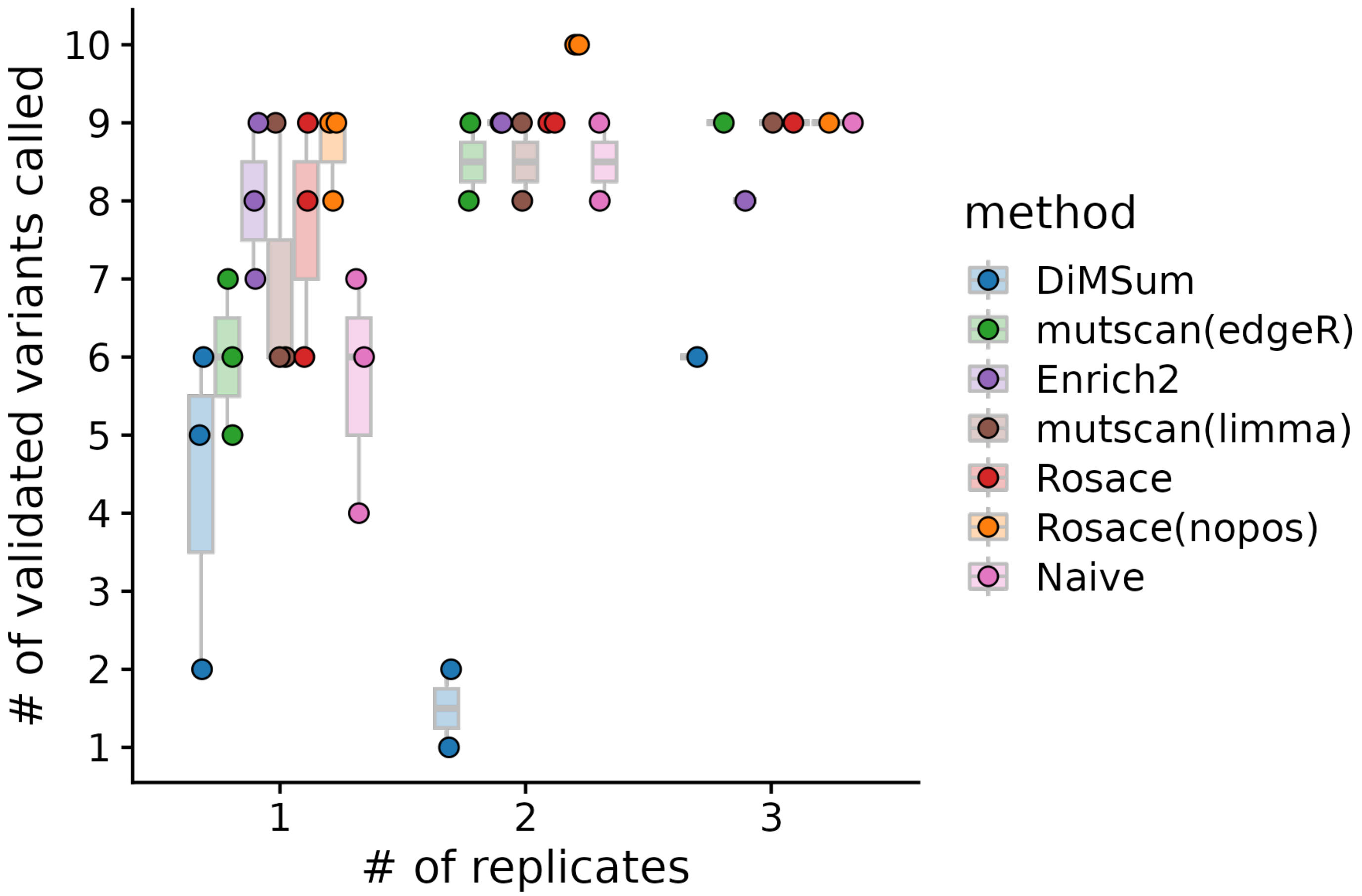

Fig. S17: **Power to detect experimentally validated variants - Number of replicates used.** Uses data from the OCT1 paper.
(DiMSum can only process two time points, and thus is disadvantaged in experiments with more than two time points, i.e., OCT1, MET, BRCA1, and BRCA1-RING.)

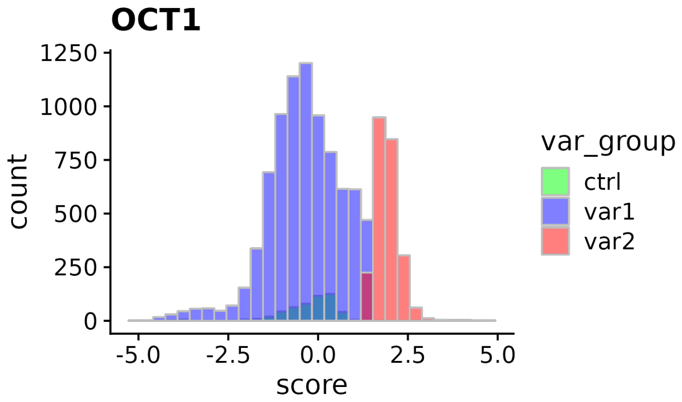

(a) Simulated score distribution of synonymous (ctrl), neutral (var1), and LOF (var2) variants.

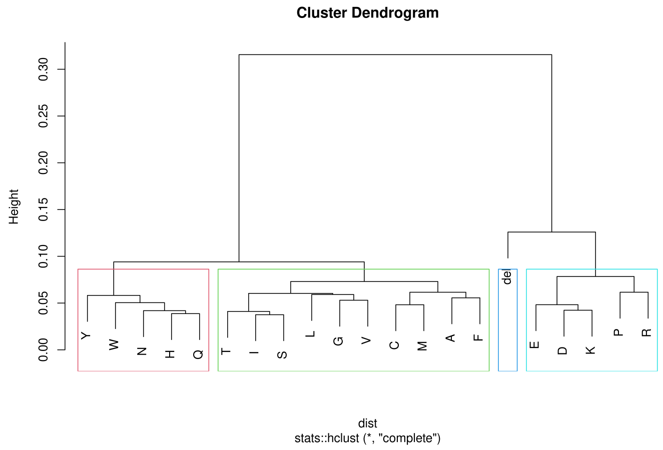

(b) Cluster dendrogram of mutations. Mutations in one group have the same variant group distribution when simulated.

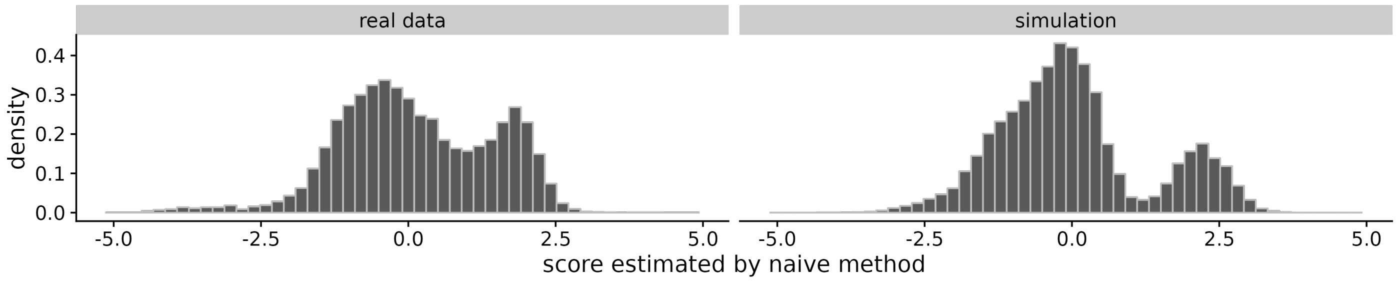

(c) Comparison of naive functional score distribution between real data and simulated data.

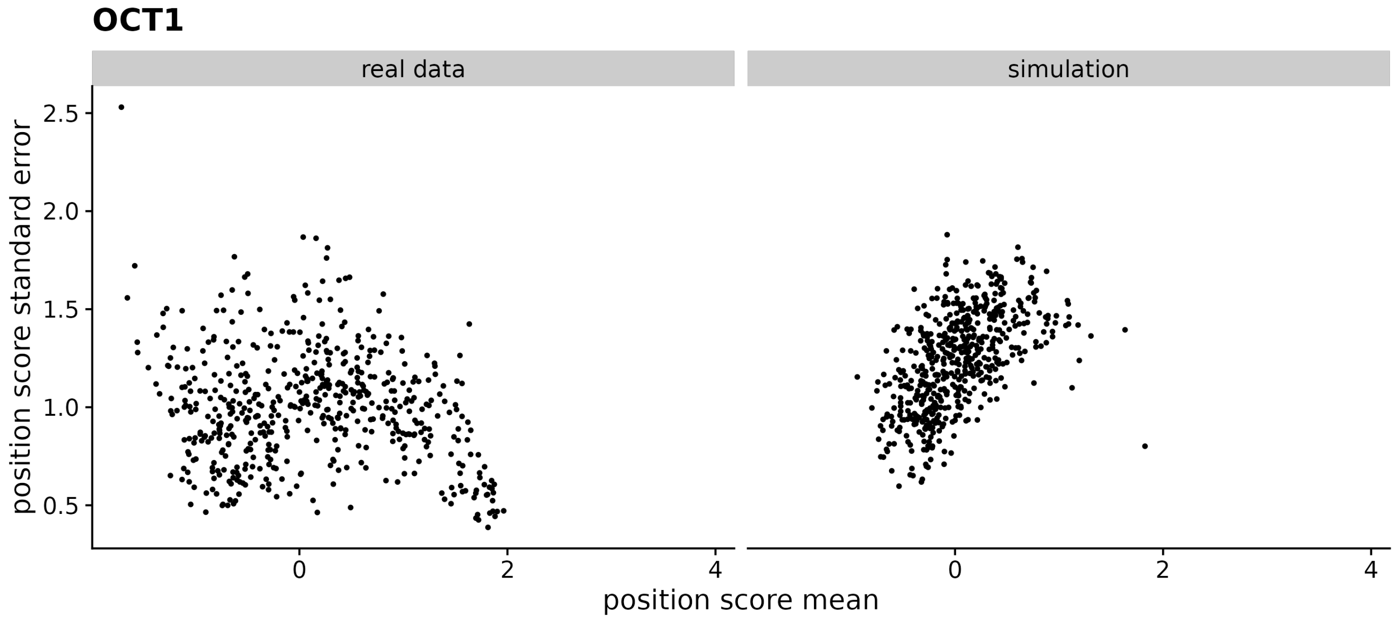

(d) Distribution of the moments of estimated naive position scores. Comparison between real data and simulated data.

Fig. S18: `Rosette` **simulation plot - OCT1.**

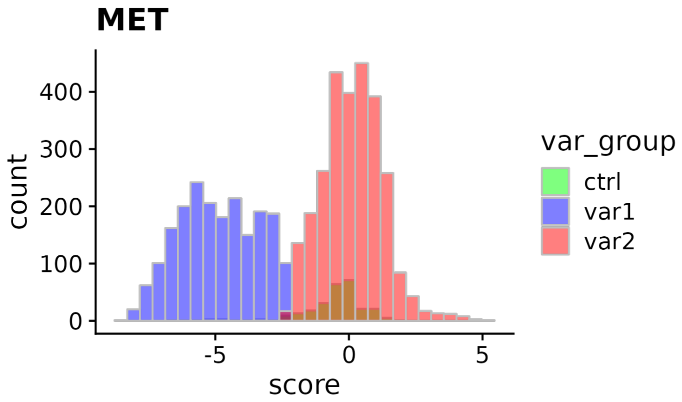

(a) Simulated score distribution of synonymous (ctrl), neutral (var2), and LOF (var1) variants.

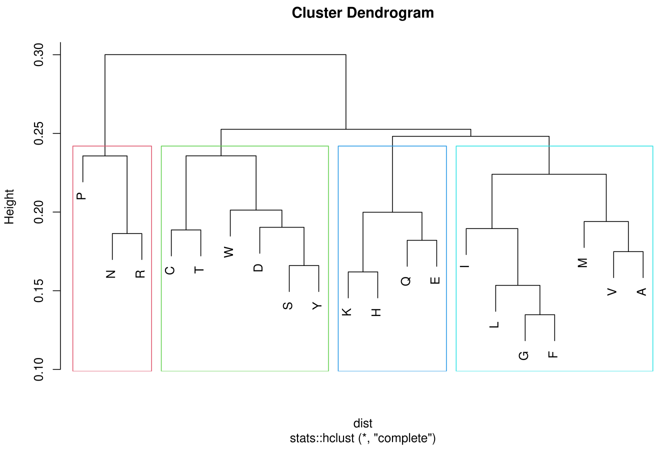

(b) Cluster dendrogram of mutations. Mutations in one group have the same variant group distribution when simulated.

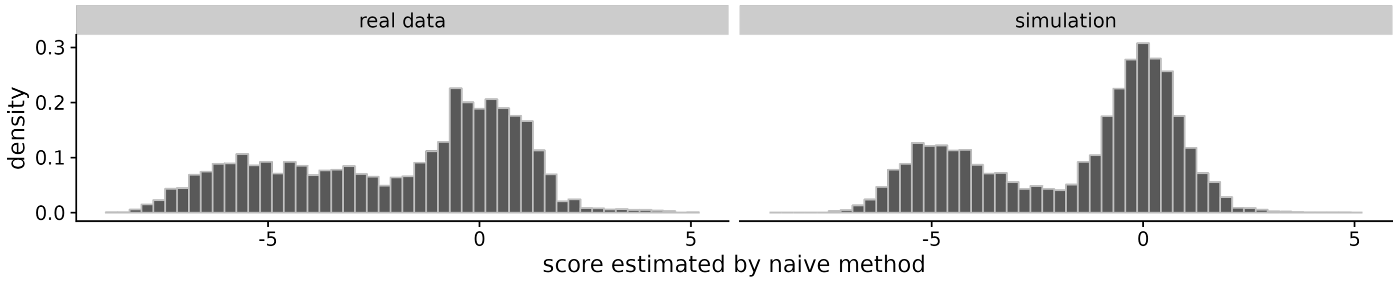

(c) Comparison of naive functional score distribution between real data and simulated data.

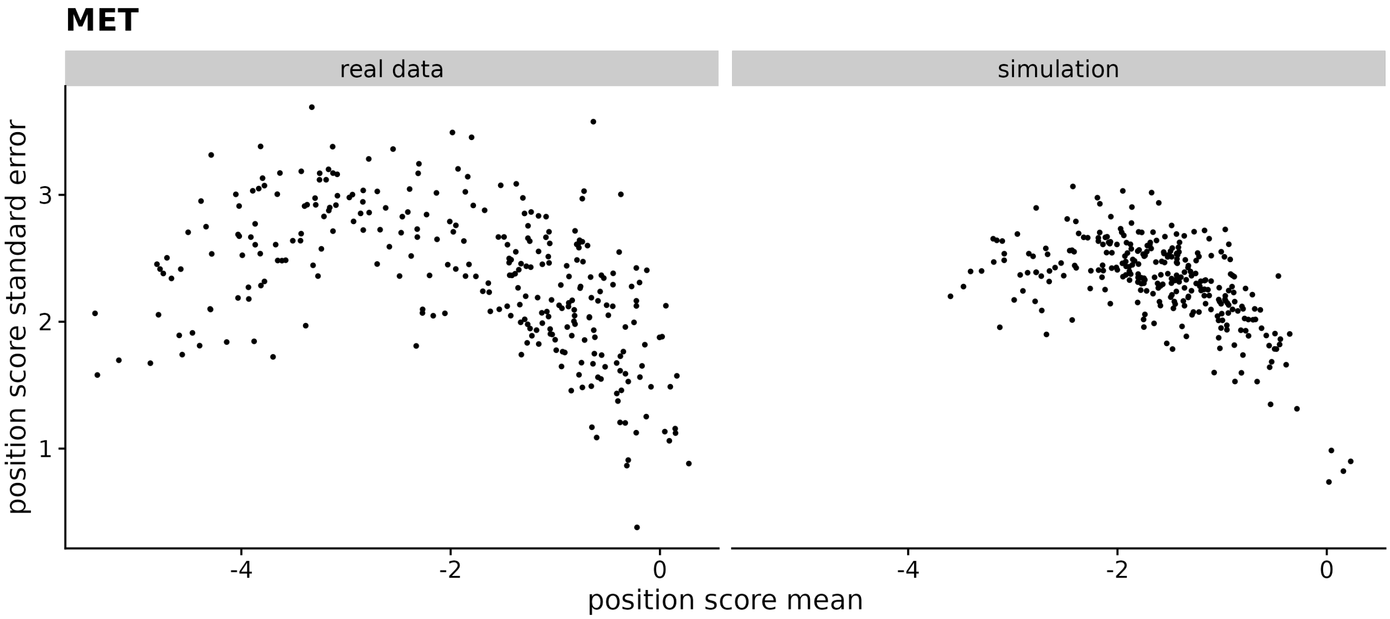

(d) Distribution of the moments of estimated naive position scores. Comparison between real data and simulated data.

Fig. S19: `Rosette` **simulation plot - MET.**

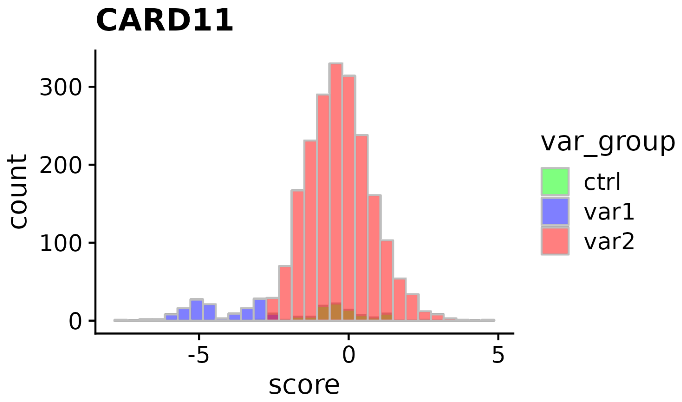

(a) Simulated score distribution of synonymous (ctrl), neutral (var2), and LOF (var1) variants.

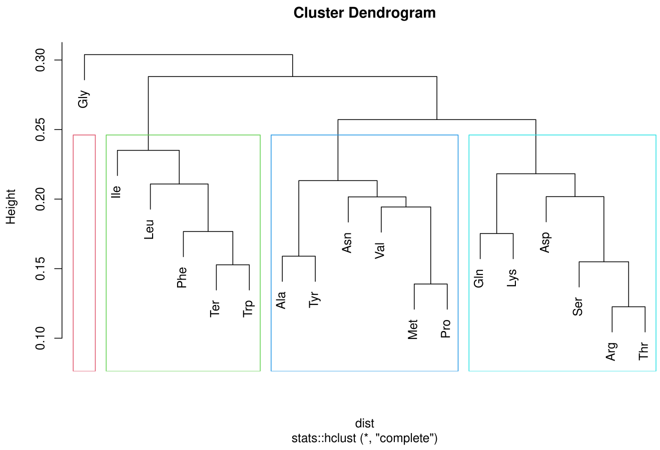

(b) Cluster dendrogram of mutations. Mutations in one group have the same variant group distribution when simulated.

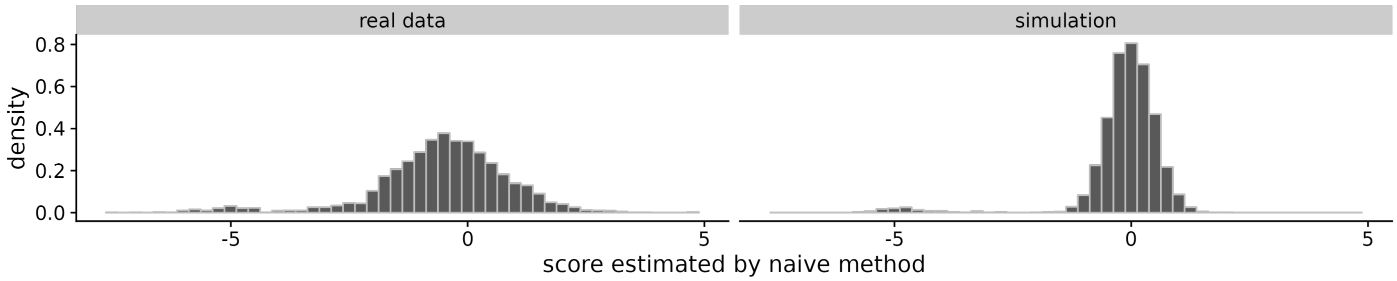

(c) Comparison of naive functional score distribution between real data and simulated data.

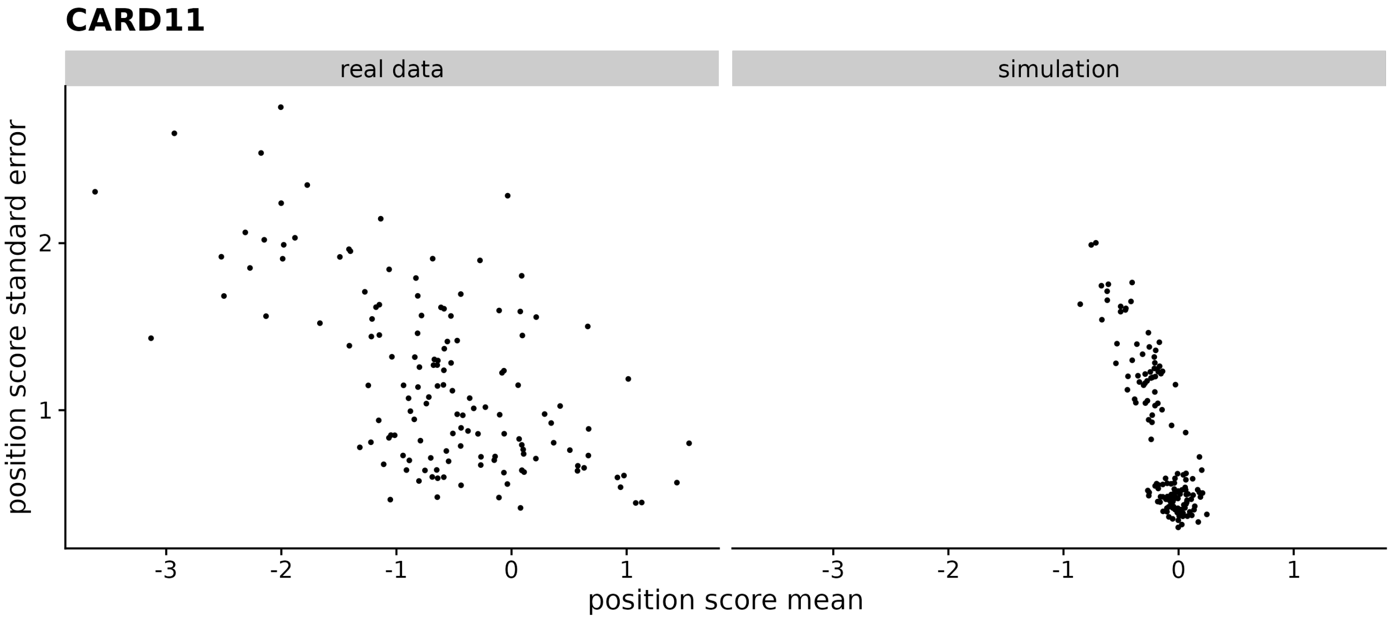

(d) Distribution of the moments of estimated naive position scores. Comparison between real data and simulated data.

Fig. S20: `Rosette` **simulation plot - CARD11.**

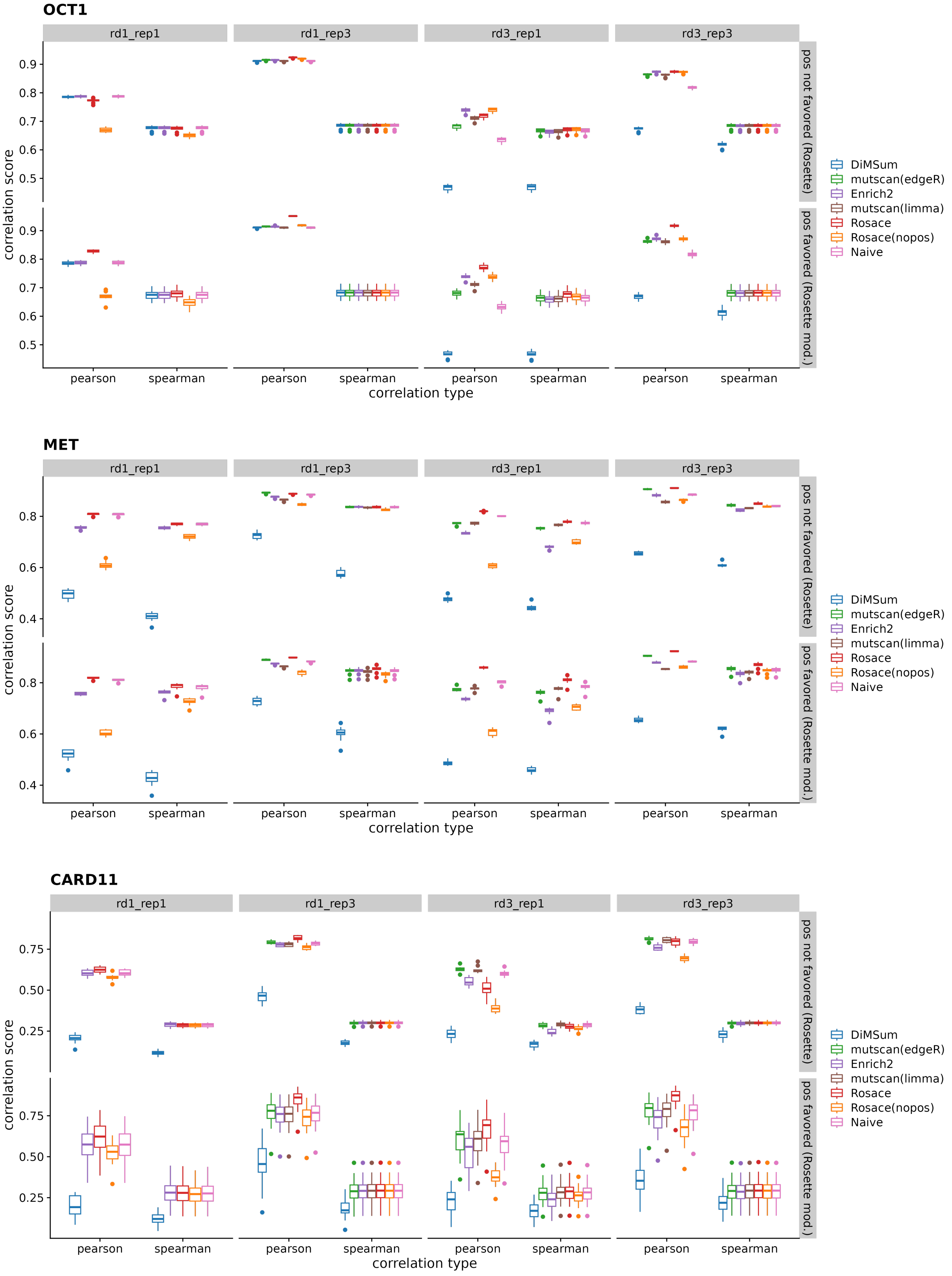

Fig. S21: **Correlation: `Rosette` simulated true effects and estimated effects.** `Rosette` simulates the true effects and counts of each variant, and `Rosace` is applied to said simulated counts to produce estimated effects. This procedure is performed 10 times. "rd$T$_rep$R$" stands for T selection rounds ($T+1$ time points) and R replicates.
(DiMSum can only process two time points, and thus is disadvantaged in experiments with more than two time points, i.e., OCT1, MET, BRCA1, and BRCA1-RING. mutscan does not support data with only 1 replicate and 1 round.)

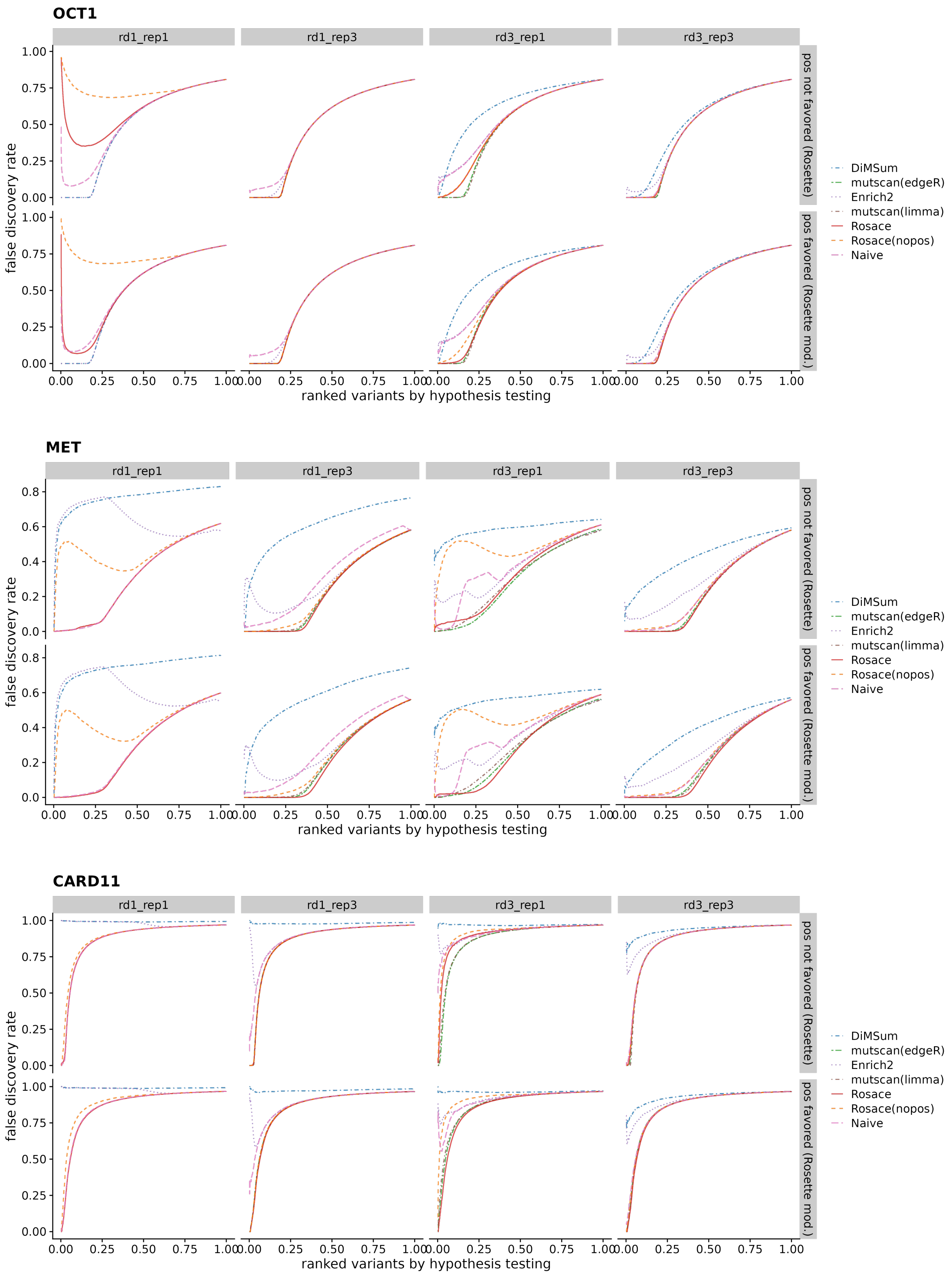

Fig. S22: `Rosette` **simulation: variant rank cutoff plotted against false discovery rate.** `Rosette` simulates the true effects and counts of each variant, and `Rosace` is applied to said simulated counts to produce estimated effects. This procedure is performed 10 times. The false discovery rate is obtained by taking the mean at each rank. "rd$T$_rep$R$" stands for T selection rounds ($T + 1$ time points) and R replicates.
(DiMSum can only process two time points, and thus is disadvantaged in experiments with more than two time points, i.e., OCT1, MET, BRCA1, and BRCA1-RING. mutscan does not support data with only 1 replicate and 1 round.)

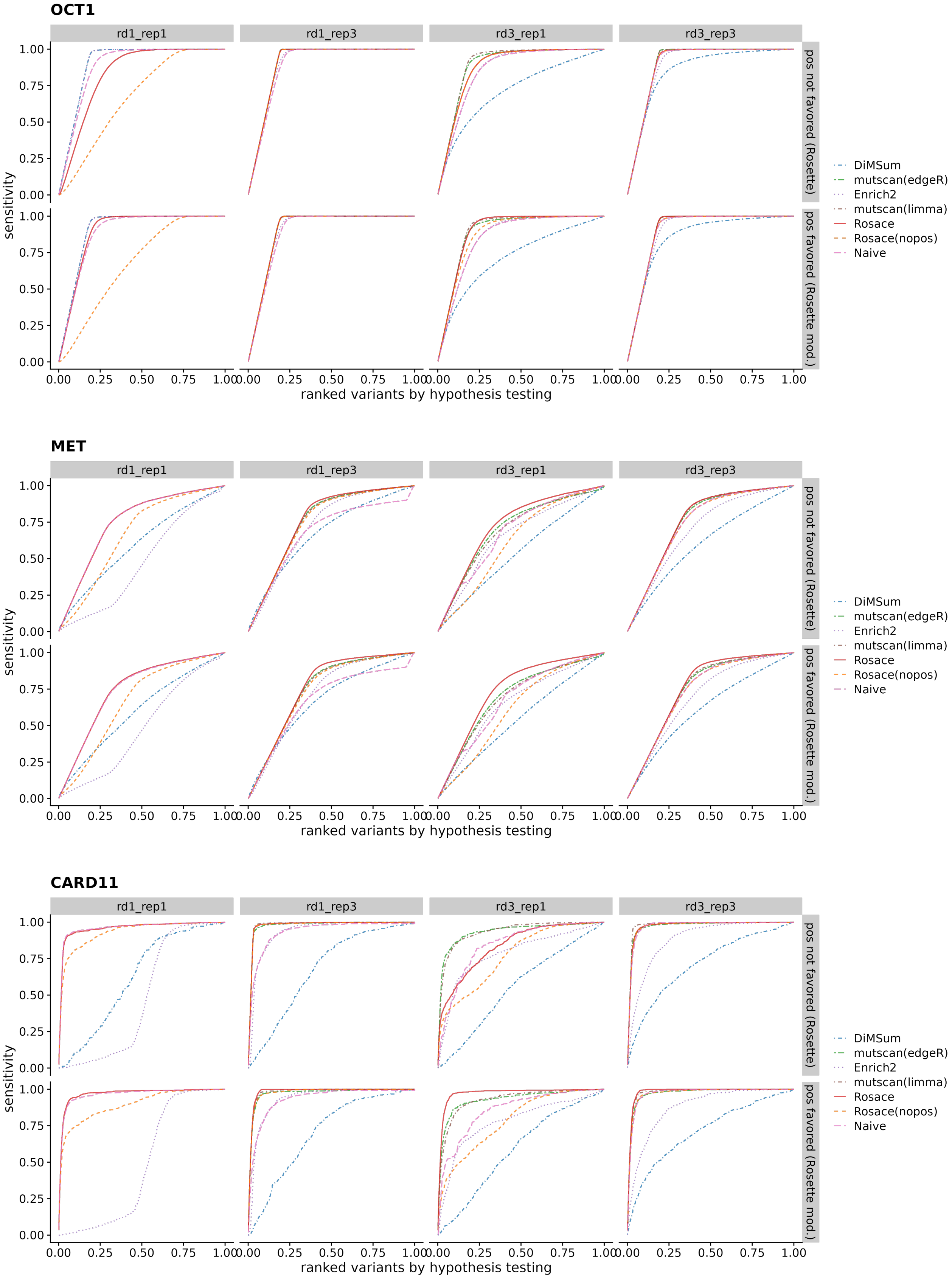

Fig. S23: `Rosette` **simulation: variant rank cutoff plotted against sensitivity.** `Rosette` simulates the true effects and counts of each variant, and `Rosace` is applied to said simulated counts to produce estimated effects. This procedure is performed 10 times. The sensitivity is obtained by taking the mean at each rank. "rd$T$_rep$R$" stands for T selection rounds ($T+1$ time points) and R replicates.

(DiMSum can only process two time points, and thus is disadvantaged in experiments with more than two time points, i.e., OCT1, MET, BRCA1, and BRCA1-RING. mutscan does not support data with only 1 replicate and 1 round.)

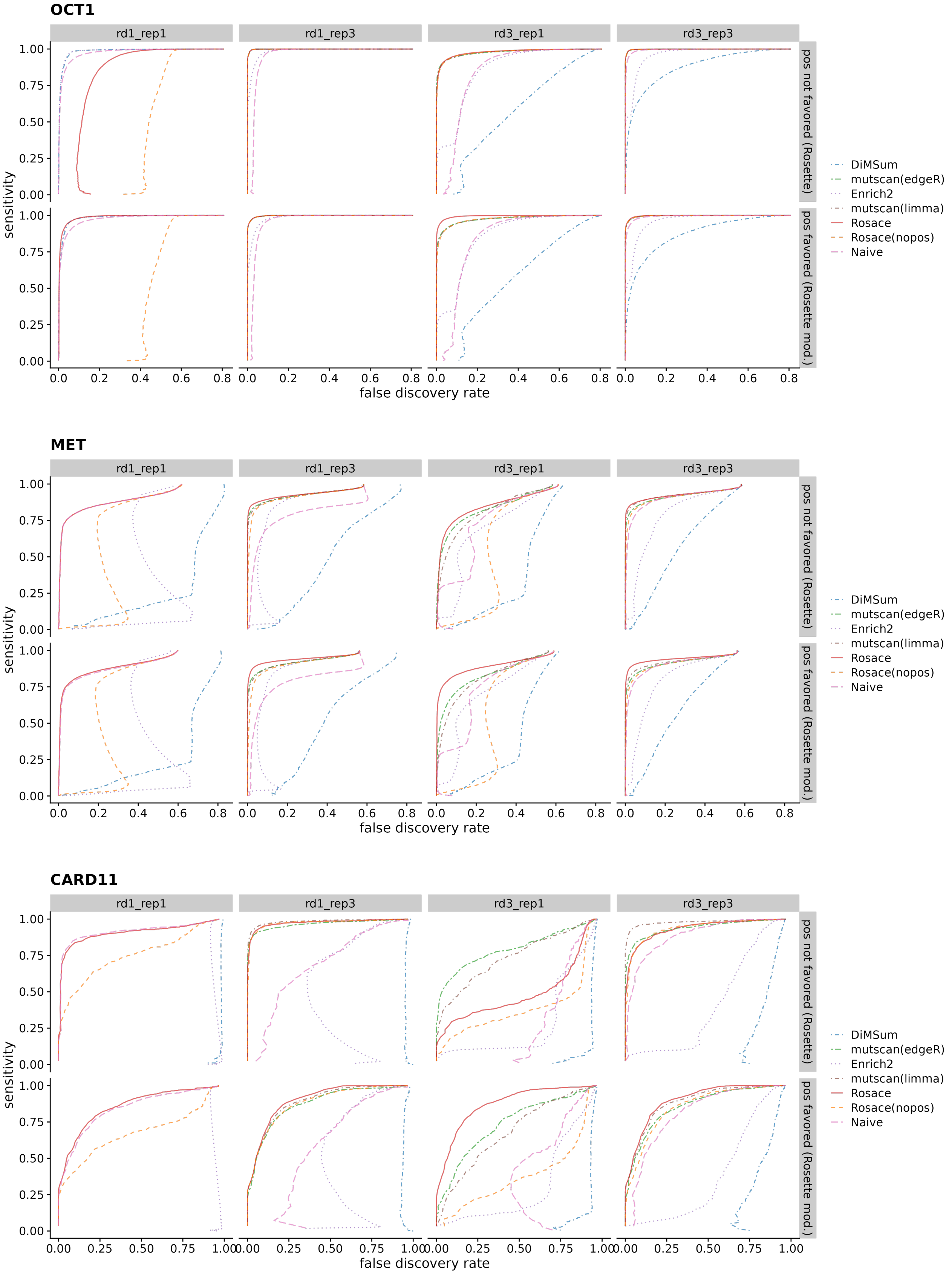

Fig. S24: Rosette **simulation: false positive rate plotted against sensitivity.** Rosette simulates the true effects and counts of each variant, and Rosace is applied to said simulated counts to produce estimated effects. This procedure is performed 10 times. The false discovery rate and the sensitivity are obtained by taking the mean at each rank. "rd$T$_rep$R$" stands for T selection rounds ($T+1$ time points) and R replicates.
(DiMSum can only process two time points, and thus is disadvantaged in experiments with more than two time points, i.e., OCT1, MET, BRCA1, and BRCA1-RING. mutscan does not support data with only 1 replicate and 1 round.)

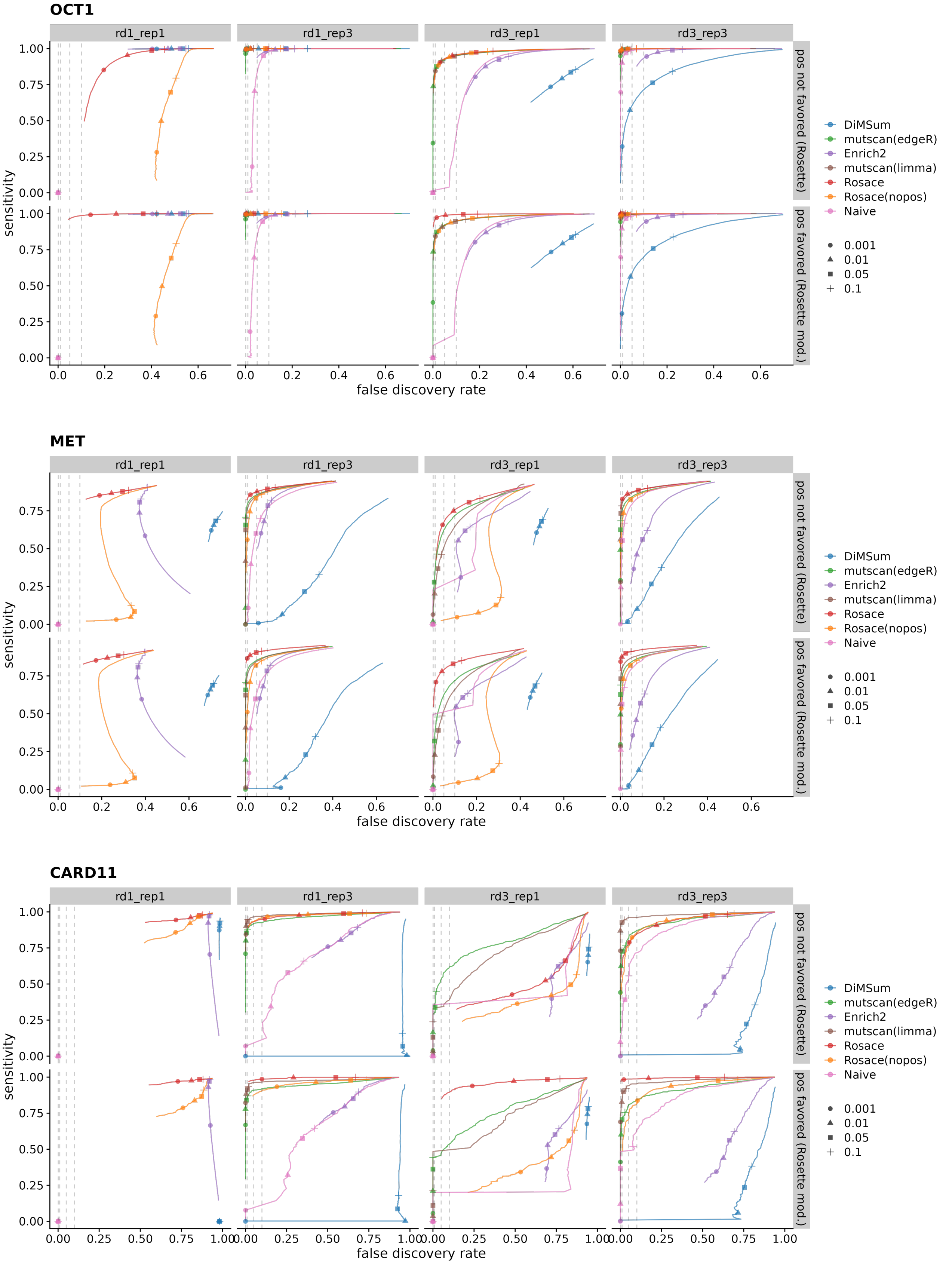

Fig. S25: `Rosette` **simulation: false positive rate plotted against sensitivity.** `Rosette` simulates the true effects and counts of each variant, and `Rosace` is applied to said simulated counts to produce estimated effects. This procedure is performed 10 times. The sensitivity is obtained by taking the mean at each test statistics cutoff (highlighted by the shape). "rd$T$_rep$R$" stands for T selection rounds ($T + 1$ time points) and R replicates.

(DiMSum can only process two time points, and thus is disadvantaged in experiments with more than two time points, i.e., OCT1, MET, BRCA1, and BRCA1-RING. mutscan does not support data with only 1 replicate and 1 round.)

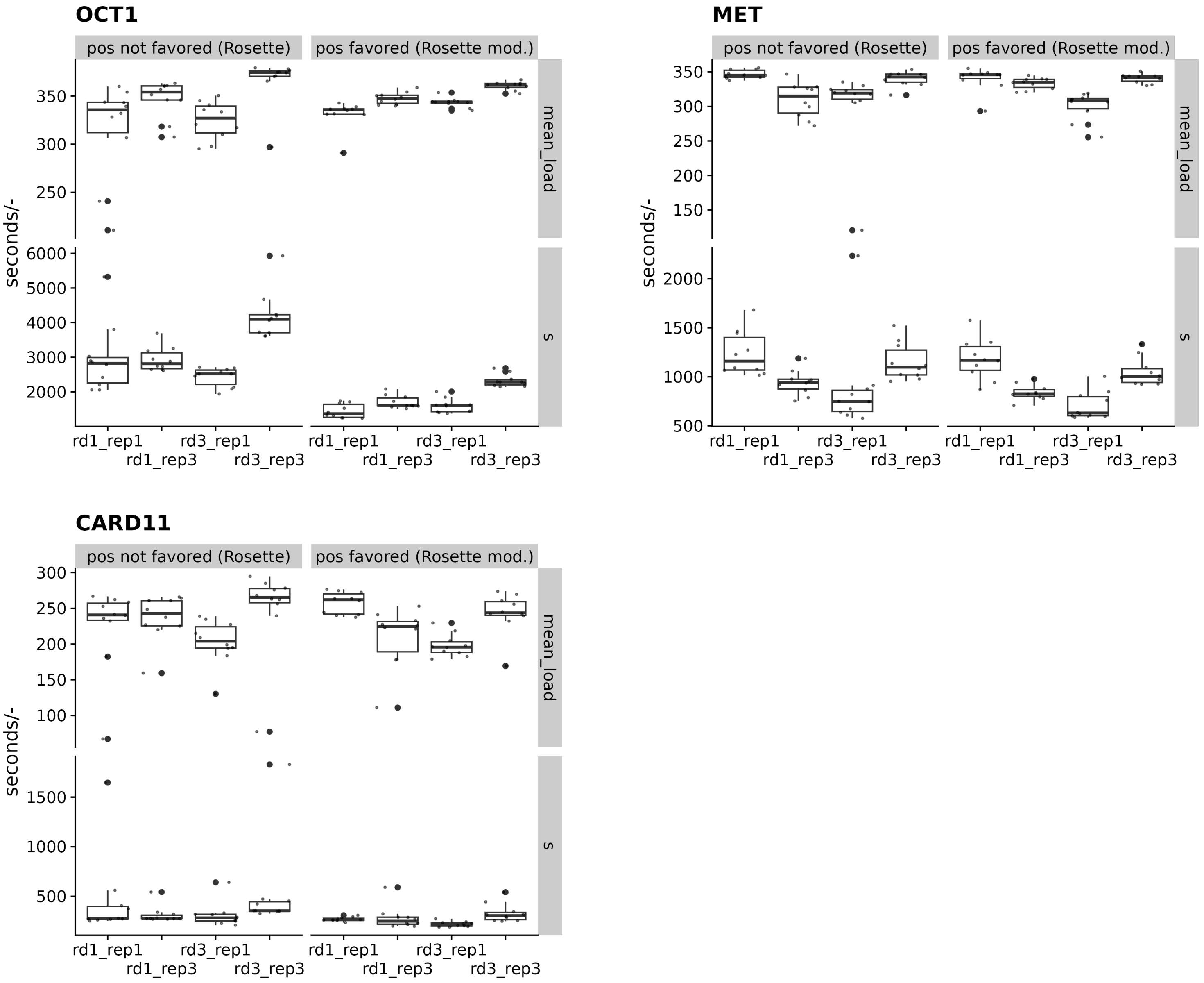

Fig. S26: `Rosette` **simulation:** `Rosace` **computation time.** `Rosette` simulates the true effects and counts of each variant, and `Rosace` is applied to said simulated counts to produce estimated effects. This procedure is performed 10 times. "rd$T$_rep$R$" stands for T selection rounds ($T + 1$ time points) and R replicates.

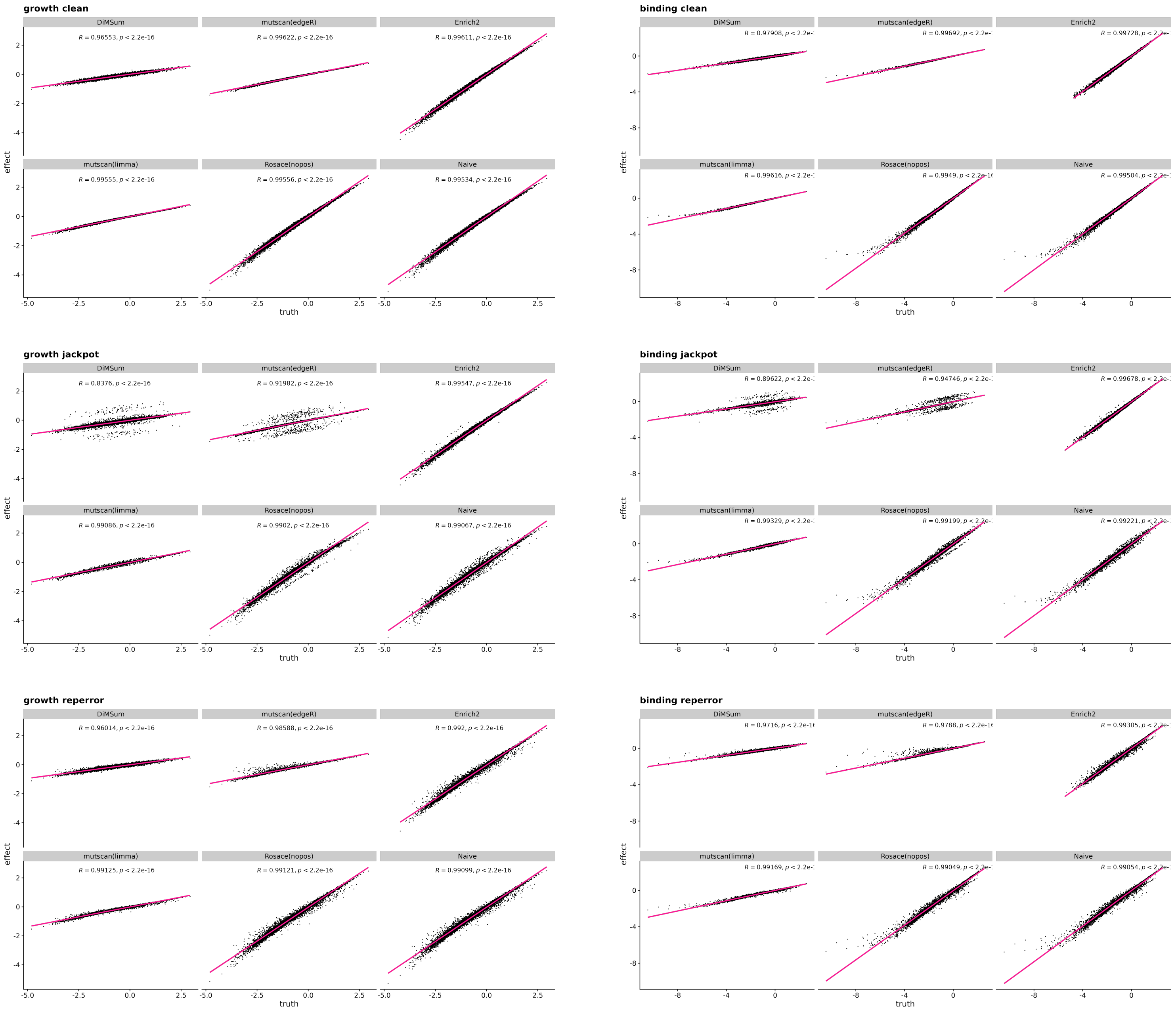

Fig. S27: **Data analysis tools' performance on *Enrich2*'s simulation.** "Growth" and "binding" stand for whether the screen is a binding screen or a growth screen. "Clean", "jackpot", and "reperror" correspond to clean condition, condition with sequencing error, and condition with replication error. (For details, see the original *Enrich2* paper Rubin et al. [2017]. Since *Enrich2* does not generate positional information, `Rosace` without the positional component ("nopos") is used instead of the original.
