# Supplementary material for: Rosace: a robust deep mutational scanning analysis framework employing position and mean-variance shrinkage
Source: Genome Biol. 2024 May 24;25:138. doi: 10.1186/s13059-024-03279-7 (PMC11127319; doi:10.1186/s13059-024-03279-7)
Supplement: Supplementary file 2 — Additional file 2: Review history. [file 13059_2024_3279_MOESM2_ESM.docx]

**1^st^ round**

**Reviewer 1**

In this study, the authors introduce Rosace, a tool for analysing growth-based deep mutational scanning data. This is a very important problem, with the rapid proliferation of DMS studies, and I am sure there is considerable scope for improving computational analysis and interpretation of this data. They also developed another tool, Rosette, for simulating DMS data, which they use for benchmarking. The underlying premise appears to be sound. The results look good, and the authors make their tools available, which will allow independent evaluation of their approach. I do think this is potentially a very valuable contribution to the community and am supportive of publication, but I do have a few questions that should be addressed.

The biggest innovation of Rosace appears to be the incorporation of positional information. On the face of it, this seems very sensible, as we know that different variants at the same residue often have highly correlated fitness scores. However, there are also some potential issues that arise from this approach.

How are variants other than single amino acid substitutions dealt with? For synonymous variants, it seems they are all grouped together, across the entire protein. If this is the case, then is Rosace essentially being told which variants are synonymous, and so any benchmarking based on the calling of synonymous variants (e.g. Fig 3) is then not really a fair comparison with other methods that aren't given this information? A better comparison might be to use gnomAD population missense variants, or say, the ~10% least damaging mutations as predicted with an evolution-based variant effect predictor.

Nonsense variants are mentioned, but I see no details on how they are dealt with. The positional information would be of little to no value here, as the vast majority across a protein will usually lead to a complete loss of function. Similarly, indels of varying length are mentioned, is positional information used for these?

What about combinations of variants in the same plasmid? Even if this was not the goal of mutagenesis, these are very often present in DMS libraries, and lots of studies are interested in their properties.

Does using positional information lead to information loss about the varying effects of different amino acid substitutions at the same position? These can often be very informative for understanding molecular mechanisms, e.g. specific patterns of tolerated substitutions at a position can identify membrane-binding regions or active sites. Is there a way to benchmark relative performance for different variants at the same position? Or at the least, discuss this potential issue.

Related to the above, ACMG/AMP guidelines allow the presence of a different pathogenic amino acid substitution at the same position to be used as 'moderate' evidence in genetic diagnosis (PM5). If positional information is used in processing of DMS data, it potentially confounds the use of DMS measurements (well-established functional assay, PS3) as independent 'strong' evidence for diagnosis.

Dimsum is excluded form the OCT1 test because it only uses data from two timepoints, but surely just two timepoints of OCT1 data could be used here to include it, as is done later for the Rosette simulations.

**Reviewer 2**

The authors present Rosace, a new method for analyzing deep mutational scanning data with improved statistical properties compared to other previously-published tools. I found the paper to be very clear and well motivated, and I appreciated how the authors described the problem being solved and the limitations of the method in terms of applicable experimental designs.

I wanted to try to install and run the examples presented in the vignettes. However, as an infrequent R user I was unable to get the program to install properly on a Linux system with full superuser privileges (Ubuntu 22.04.3 LTS) despite several hours of effort. This mostly appeared to be due to missing dependencies that were undocumented by the authors and not clearly reported by R itself.

I know that the authors don't deserve blame for the state of R packaging, but they are responsible for delivering a software implementation where an interested user can run the examples and attempt to apply it to their own work. I strongly recommend that the authors perform and document a "from scratch" installation of Rosace and attempt to document the process, as well as provide more links to community resources for dealing with problems installing problematic packages like devtools.

Ideally, the authors will also provide a Docker image or similar pre-built environment that prospective users can use to try out the software. If I wasn't trying to get things running for this review I would have given up on using Rosace much earlier.

My comments on the manuscript and the results described in the paper are below.

Major comments:

1. Rosace is presented as a tool that works on growth-based DMS datasets. Because of this, I was expecting something that would explicitly try to model or estimate a growth rate, but I didn't see any such terms? It seemed to me like Rosace is suitable for any enrichment-depletion based experimental design, including binding (like the very first DMS [PMID 20711194]). The authors should consider making this claim when the tool is introduced if they think it's appropriate. I completely agree with their assessment that fluorescence-based assays are very different and probably not suitable for analysis with Rosace, although groups have used fluoresecence to achieve enrichment-depletion before.

2. The authors only looked at human datasets, although there's a lot of yeast, bacterial, and viral datasets out there. Since the tool is claiming to be quite general, it would be a nice (but optional) addition to analyze some datasets from other organisms.

3. The OCT1 assay is "reversed" from how most DMS experiments are structured, in that loss of function leads to enrichment rather than depletion. I know this is likely the dataset that the authors were most familiar with and therefore makes sense to feature, but I'm not sure it's the best example. It would be good to have an example of a more "standard" assay where loss of function leads to depletion as well, or the authors could acknowledge that this is an unusual case in the text.

4. The authors assume that the proportion of synonymous variants in the pool is invariant throughout the experiment. This is certainly violated in every DMS experiment, as the selection pressure changes over time as low-performing variants drop out and high-performing variants increase in abundance. The authors should add a little something here about how much violating this assumption will affect the results. Obviously, all models require imperfect assumptions, but this one jumped out at me as something that needed to be acknowledged.

5. The should compare the Rosette simulation framework to the simdms simulation framework that was published in the Enrich2 paper. Right now it reads as if Rosette is the first software to try to solve this problem. It would be interesting to know how the authors' approach differed from this previous work (and any other work) on simulating DMS datasets.

6. In the discussion the authors describe some applications for Rosette. Is Rosette able to tell researchers how deeply they need to sequence their libraries to get good results? This would be a very practical application if so.

7. At the end of the discussion, the authors suggest that Rosace could be used to learn about multi-dimensional relationships. The tone of this part of the discussion is fairly aspirational and speculative. However, we have had datasets to test these outcomes for many years! For example, Stiffler, Hekstra, and Ranganathan produced an antibiotic resitance dataset with multiple antibiotics and concentrations in 2015 [PMID 25723163]. Steinberg and Ostermeier looked at antibiotic resistance in multiple alleles of the same gene in 2016 [PMID 27173379]. If this is easy to do with Rosace, the authors should apply their tool to these or other similar non-human datasets. If (as I suspect) this is still quite challenging technically, the discussion should be toned down so that it's clear that this is a potential future application that is still a ways off due to practical challenges with the analysis.

8. The authors based Rosette's assumptions on the OCT1 and MET datasets. It would be very interesting to know how different the saturation genome editing datasets are (BRCA1, CARD11) in their simulation parameters.

9. The availability of data and materials section should list the software license and the software version being presented. The authors should also archive all repositories using Zenodo or some other similar stable repository that mints a DOI on each release.

Minor comments:

1. The authors cite several recent topical papers in the introduction, but appear to be missing several important foundational studies. For example, the authors don't cite the VAMP-seq paper [PMID 29785012] for protein stability or fluorescence-based assays. The authors don't cite any work from Dan Bolon's lab, despite this work in yeast systems being fundamental for establishing growth-based DMS.

2. There are no citations where DMS is defined. The authors should cite some reviews, minimally Fowler & Fields 2014 [PMID 25075907]. There are many to choose from depending on whether the authors are more interested in clinical applications [PMID 36055970] or biophysical modelling [PMID 30712981]. These two are just quick suggestions; there are many such examples and the authors should choose a couple of references that they prefer or find especially useful. The authors do cite a couple of reviews in refs 15 and 16, but these seem out of place.

3. The authors cite dms_tools in kind of a weird place (ref 11) and don't actually list it with the other software packages. While it's fine that the authors didn't run it for thde paper, it should be listed along with the other software.

4. The authors cite two relatively recent papers for "multiple time points" and assert this is a recent innovation, but Starita et al. 2015 [PMID 25823446] did this relatively early in the history of the field, and this dataset was analyzed in the Enrich2 paper. The claim about this being a new innovation should be removed, and the authors should consider this and any other applicable citations.

5. The authors mention analyzing DMS in a position-specific manner at the top of page 3 and cite a couple of very broad papers (one protocol paper and one very early review). The authors should choose and cite some specific studies that do this instead.

6. At the end of section 2.1 the authors mention being able to process data from Enrich2 "for other protocols." The most obvious application here is barcoded libraries, and I suggest that the authors add a phrase like ", such as barcoded sequencing libraries" at the end of this sentence. Barcoded libraries are fairly common, and it would be nice to point out how userds can analyze them using Rosace.

7. In section 2.2, the authors don't talk about how indels are handled. It would be good to have a sentence there about how the model interprets in-frame insertions or deletions of a single amino acid (maybe it doesn't?), as this is a common feature of a lot of DMS libraries.

8. In the Figure 1 legend, the authors should add something about the two different types of replicate structures show in panel B, with replication pre- and post- transfection. It's great that these are depicted, but there should be something in the legend.

9. In the Figure 2 legend, the plate diagram (panel C) is not interpretable without a description of parameters, but the authors ask the reader to consult the methods section. I would like the authors to either put some brief definitions here or move the plate diagram out of this figure and into the methods section near the parameter definitions.

10. At the end of section 2.2 the authors mention the "MSH data". This should be "MSH2 data".

11. Sometimes the authors write tools as mutscan(edgeR) and mutscan(limma) or mutscan(limma-voom), and other times they just write edgeR or limma-voom. I suggest that these always be written as part of mutscan for clarity.

12. In the discussion, the authors refer to "FACS-seq" but this is predated by VAMP-seq and VAMP-seq should be cited and mentioned here.

13. The last paragraph/sentence of section 4.1 is duplicated above at the end of the first paragraph in the section.

**Authors’ response**

Reviewer #1

In this study, the authors introduce Rosace, a tool for analysing growth-based deep mutational scanning data. This is a very important problem, with the rapid proliferation of DMS studies, and I am sure there is considerable scope for improving computational analysis and interpretation of this data. They also developed another tool, Rosette, for simulating DMS data, which they use for benchmarking. The underlying premise appears to be sound. The results look good, and the authors make their tools available, which will allow independent evaluation of their approach. I do think this is potentially a very valuable contribution to the community and am supportive of publication, but I do have a few questions that should be addressed.

*Thank you very much for this assessment. We appreciate the positive feedback and are delighted to hear that our research resonated with you. We have followed your advice and revised the paper accordingly. Once again, thank you for your kind words and support.*

The biggest innovation of Rosace appears to be the incorporation of positional information. On the face of it, this seems very sensible, as we know that different variants at the same residue often have highly correlated fitness scores. However, there are also some potential issues that arise from this approach.

How are variants other than single amino acid substitutions dealt with? For synonymous variants, it seems they are all grouped together, across the entire protein. If this is the case, then is Rosace essentially being told which variants are synonymous, and so any benchmarking based on the calling of synonymous variants (e.g. Fig 3) is then not really a fair comparison with other methods that aren't given this information? A better comparison might be to use gnomAD population missense variants, or say, the ~10% least damaging mutations as predicted with an evolution-based variant effect predictor.

*Variants other than single amino acid substitutions include synonymous mutations (right below), nonsense mutations, indels of varying length (next response), and multiple mutations (further below).*

*For the Rosace method, instead of grouping all synonymous variants together, we decided to split synonymous variants into groups of about 20 synonymous variants per group (as in the number of non-synonymous mutations under a position label), in practice. This approach reduces the shrinkage on synonymous variants and makes it comparable to the variance estimation procedure on non-synonymous variants.*

*To explore the synonymous variant distribution and see if we are artificially forcing the variants to be smaller, we included a new set of correlation plots of ENRICH2 scores and Rosace scores highlighting synonymous variants in red (Fig. S2-3), showing that Rosace does not shrink synonymous variants more than ENRICH2. We also added the Spearman correlation between the two scores with and without synonymous mutations, finding that the correlation is not significantly impacted by the inclusion of*

*synonymous mutations - implying that the rank order is not significantly changed and thus Rosace does not artificially shrink the estimate of the synonymous variant posterior mean ef ect size.*

*We also added AlphaMissense prediction, EVE prediction, and ClinVar labels (downloaded from gnomAD) ROC curves as an alternative method to calculate the TF and TP rate (Fig. S6-9). However, the number of clinically verified variants is limited, and those identified usually have extreme ef ects. Furthermore, the EVE prediction only covers some regions of proteins, and since it is a prediction model, we only use the most conservative ones (10%) that are expected to have extreme ef ects. As a result, we don’t see a large dif erence between any of the method’s performance.*

Nonsense variants are mentioned, but I see no details on how they are dealt with. The positional information would be of little to no value here, as the vast majority across a protein will usually lead to a complete loss of function. Similarly, indels of varying length are mentioned, is positional information used for these?

*Nonsense variants and indels are treated the same as missense mutations and we have made it clearer in the text (Sec. 2.2 Para. 2 and Sec. 4.3 Para. 2). Furthermore, we give a new example of MET (Fig. S3). While position information is also involved in analyzing nonsense variants, its estimated ef ect still escapes the positional mean.*

*From the new graph (Fig. S3), we can see that the nonsense variant at position 1059 (red triangle) deviates much from the majority of other variants at this position. Similarly, the 1083 nonsense (green triangle) deviates much from its peers. In comparison, position 1107 variants cover a large area of the ef ect spectrum, supporting our claim that the positional shrinkage is not overly strong.*

*Indels of various lengths are labeled as a mutation of the leftmost amino acid residue (e.g. an insertion between positions 99 and 100 is labeled as position 99 and a deletion of positions 100 through 110 is labeled as position 100). The information is also added to Section 4.3.*

What about combinations of variants in the same plasmid? Even if this was not the goal of mutagenesis, these are very often present in DMS libraries, and lots of studies are interested in their properties.

*Solving this problem from first principles and estimating higher-order ef ects requires much further investigation, which we believe is out of the scope of this manuscript. However, we of er some solutions to estimate single ef ects from such data.*

*To run full-mode Rosace, users need to give a pseudo-position label to each combination of variants. On the other hand, users could run Rosace with no position mode (marked as Rosace(nopos) in the Supp figures, explained in Section 4.8). It shows very reasonable performance and is preferred in this case.*

*We also made it clear at the end of Section 2.1 that even without a position label, Rosace can be run properly. Besides, we pose this problem explicitly in the third paragraph of the Discussion section.*

Does using positional information lead to information loss about the varying effects of different amino acid substitutions at the same position? These can often be very informative for understanding molecular mechanisms, e.g. specific patterns of tolerated substitutions at a position can identify membrane-binding regions or active sites. Is there a way to benchmark relative performance for different variants at the same position? Or at the least, discuss this potential issue.

*This is a very good point that we are concerned with, however, finding a suf icient number of such sites in real experimental data for informative benchmarking is very challenging. Instead, we added a new section of deliberation on the trade-of between prior information and true data in the Discussion (paragraph 4).*

*Additionally, we can perform simulations using Rosette where we assume a position-specific model, while the true data is simulated under NO positional information. A truly robust method should be agnostic to the prior misspecification. The generative procedures of Rosette explicitly violate the prior assumptions made by Rosace (that positional information matters), but even with Rosette’s position-agnostic data, Rosace can learn important information (Fig. 6).*

Related to the above, ACMG/AMP guidelines allow the presence of a different pathogenic amino acid substitution at the same position to be used as 'moderate' evidence in genetic diagnosis (PM5). If positional information is used in processing of DMS data, it potentially

confounds the use of DMS measurements (well-established functional assay, PS3) as independent 'strong' evidence for diagnosis.

*We thank the reviewer for this important comment. We understand this concern and have addressed it by exploring aspects of the simulation and real data in additional plots (Figs. S2-4) to show that the position prior does not necessarily bias the functional score estimate so much that it distorts the conclusions. We believe that the position prior benefits the model mainly through a more stable standard error estimate enabling improved prioritization as a function of local false sign rate or other posterior ranking criteria that are a function of the variance.*

*This concern is understandable and users should be made aware of the potential confounding. However, any model invariably incorporates prior beliefs in some form. Our decision to incorporate positional information stems from the empirical observation that also inspires Criterion PM5. The decision to assign synonymous variants to artificial “control” positions partially coincides with Criterion ACMG/AMP guideline BP7 as well.*

Dimsum is excluded form the OCT1 test because it only uses data from two timepoints, but surely just two timepoints of OCT1 data could be used here to include it, as is done later for the Rosette simulations.

*We have added Dimsum to our analyses and made it clear that it is disadvantaged in scenarios where there are more than two time points (see Section 4.8).*

Reviewer #2

The authors present Rosace, a new method for analyzing deep mutational scanning data with improved statistical properties compared to other previously-published tools. I found the paper to be very clear and well motivated, and I appreciated how the authors described the problem being solved and the limitations of the method in terms of applicable experimental designs.

*We thank the Reviewer for this positive assessment of our manuscript. We appreciate all the paper suggestions by Reviewer 2 which helped us dig deeper into the history and development of DMS.*

I wanted to try to install and run the examples presented in the vignettes. However, as an infrequent R user I was unable to get the program to install properly on a Linux system with full superuser privileges (Ubuntu 22.04.3 LTS) despite several hours of effort. This mostly appeared to be due to missing dependencies that were undocumented by the authors and not clearly reported by R itself.

I know that the authors don't deserve blame for the state of R packaging, but they are responsible for delivering a software implementation where an interested user can run the examples and attempt to apply it to their own work. I strongly recommend that the authors perform and document a "from scratch" installation of Rosace and attempt to document the process, as well as provide more links to community resources for dealing with problems installing problematic packages like devtools.

Ideally, the authors will also provide a Docker image or similar pre-built environment that prospective users can use to try out the software. If I wasn't trying to get things running for this review I would have given up on using Rosace much earlier.

*We understand that the disparate environment users are in when running our tool could cause problems and that we shall take the responsibility to address this problem. We have published a Docker image (cbmacdo/rosace-docker), and addressed the problem on the installation page. We have also assisted other collaborators in using Rosace both locally and on the server. If problems remain, please contact the authors.*

My comments on the manuscript and the results described in the paper are below. Major comments:

1. Rosace is presented as a tool that works on growth-based DMS datasets. Because of this, I was expecting something that would explicitly try to model or estimate a growth rate, but I didn't see any such terms? It seemed to me like Rosace is suitable for any enrichment-depletion based experimental design, including binding (like the very first DMS [PMID 20711194]). The authors should consider making this claim when the tool is introduced if they think it's appropriate. I completely agree with their assessment that fluorescence-based assays are very

different and probably not suitable for analysis with Rosace, although groups have used fluoresecence to achieve enrichment-depletion before.

*The data generation model of Rosace is explicitly trying to estimate a growth rate. Presented in Section 4.6, the resulting score of this linear regression after normalization happens to be a transformation of the true growth rate. We also agree that Rosace can be applied to “binding experiments” presented in the Enrich2 paper, where a portion of cells is selected at each time point, using techniques such as fluorescence. The expectation of the slope of linear regression will be a log transformation of the selection proportion. However, we did not benchmark Rosace using binding-experiment simulations as we believe that this is a large extension, and we did not benchmark on real data so we do not want to make an overstatement of Rosace's performance on binding experiments. This is an area of active work we are interested in, but we believe is outside of the scope of this manuscript.*

*Fluorescence-based assays we mention here are mainly 4-bin or 8-bin FACS screens where we have the potential to learn the underlying fluorescence distribution of each mutation beyond mean shifting. The mutated protein could have the ef ects of half completely loss-of-function and half neutral ([100, 0, 0, 100]), or all mildly loss-of-function ([0, 100, 100, 0]), which can both show a score of 0 in terms of the slope by either Enrich2 or Rosace but the bin count interpretations are conceptually very dif erent.*

*We have also cited the very first DMS paper in the introduction, added Rosace’s potential utility for the binding experiment in the discussion, and made clear implications for the FACS screen we refer to in the discussion.*

2. The authors only looked at human datasets, although there's a lot of yeast, bacterial, and viral datasets out there. Since the tool is claiming to be quite general, it would be a nice (but optional) addition to analyze some datasets from other organisms.

*We added a bacterial dataset to our analyses (Cohesin) and human protein datasets done on murine cells and phage. See Table S1.*

3. The OCT1 assay is "reversed" from how most DMS experiments are structured, in that loss of function leads to enrichment rather than depletion. I know this is likely the dataset that the authors were most familiar with and therefore makes sense to feature, but I'm not sure it's the best example. It would be good to have an example of a more "standard" assay where loss of function leads to depletion as well, or the authors could acknowledge that this is an unusual case in the text.

*That is a valid concern. We have added the MET assay to our analyses (Figs. S10-12) as explained in section 2.3.*

4. The authors assume that the proportion of synonymous variants in the pool is invariant throughout the experiment. This is certainly violated in every DMS experiment, as the selection pressure changes over time as low-performing variants drop out and high-performing variants increase in abundance. The authors should add a little something here about how much

violating this assumption will affect the results. Obviously, all models require imperfect assumptions, but this one jumped out at me as something that needed to be acknowledged.

*We have revised the text in Section 2.4 to show more clearly that we do not actually make such an assumption in the analysis tool Rosace or the simulation framework Rosette. We compensate for the phenomenon that the proportion of synonymous variants can indeed change due to selection pressure by (1) grouping normalized count errors by the magnitude of the count in Rosace and (2) incorporating an overdispersion factor in Rosette.*

5. The should compare the Rosette simulation framework to the simdms simulation framework that was published in the Enrich2 paper. Right now it reads as if Rosette is the first software to try to solve this problem. It would be interesting to know how the authors' approach differed from this previous work (and any other work) on simulating DMS datasets.

*We have added the comparison and mentioned the Enrich2 simulation at the end of the introduction.*

*In short, Rosette directly infers the dispersion of count and score distributions from real data and generates counts that reflect the true level of noise in the real experiment. We show that in Supplementary Figure 1 other existing methods that sample from a multinomial distribution do not capture enough noise in the real data.*

*We also added the result of the Enrich2 simulation in Section 2.4 where all methods show near-perfect performance in Supplementary Figure 24 (correlation score > 0.99).*

6. In the discussion the authors describe some applications for Rosette. Is Rosette able to tell researchers how deeply they need to sequence their libraries to get good results? This would be a very practical application if so.

*We believe Rosette is good enough for method benchmarking but have not yet evaluated whether it is adequate for experimental design. We remain conservative on the utility of Rosette since it is not validated by the experiments, even though we believe it is a huge advance from the state of the art. We revised the tone in the Discussion section to reflect that evaluation is still needed, and it merits a separate paper to fully evaluate this idea.*

7. At the end of the discussion, the authors suggest that Rosace could be used to learn about multi-dimensional relationships. The tone of this part of the discussion is fairly aspirational and speculative. However, we have had datasets to test these outcomes for many years! For example, Stiffler, Hekstra, and Ranganathan produced an antibiotic resitance dataset with multiple antibiotics and concentrations in 2015 [PMID 25723163]. Steinberg and Ostermeier looked at antibiotic resistance in multiple alleles of the same gene in 2016 [PMID 27173379]. If this is easy to do with Rosace, the authors should apply their tool to these or other similar non-human datasets. If (as I suspect) this is still quite challenging technically, the discussion should be toned down so that it's clear that this is a potential future application that is still a ways off due to practical challenges with the analysis.

*Thank you for suggesting these datasets. While Rosace cannot be immediately deployed to analyze multi-phenotype datasets, the adaptation to such is ongoing and merits a paper of its own. We toned down the discussion and pointed out some practical challenges.*

8. The authors based Rosette's assumptions on the OCT1 and MET datasets. It would be very interesting to know how different the saturation genome editing datasets are (BRCA1, CARD11) in their simulation parameters.

*We added the CARD11 dataset to the simulation, included in the supplementary figures 17, 18C, 19C, 20C, 21C, 22C and 23C.*

9. The availability of data and materials section should list the software license and the software version being presented. The authors should also archive all repositories using Zenodo or some other similar stable repository that mints a DOI on each release.

*We uploaded the Rosace repositories to Zenodo and created a DOI (10.5281/zenodo.10814911).* Minor comments:

1. The authors cite several recent topical papers in the introduction, but appear to be missing several important foundational studies. For example, the authors don't cite the VAMP-seq paper [PMID 29785012] for protein stability or fluorescence-based assays. The authors don't cite any work from Dan Bolon's lab, despite this work in yeast systems being fundamental for establishing growth-based DMS.

*Thank you very much for the suggestions. We cited the VAMP-seq paper and a paper on Hsp90 by Flynn (2020) from Dan Bolon’s lab [PMID 32129763] in both the introduction and discussion.*

2. There are no citations where DMS is defined. The authors should cite some reviews, minimally Fowler & Fields 2014 [PMID 25075907]. There are many to choose from depending on whether the authors are more interested in clinical applications [PMID 36055970] or biophysical modelling [PMID 30712981]. These two are just quick suggestions; there are many such examples and the authors should choose a couple of references that they prefer or find especially useful. The authors do cite a couple of reviews in refs 15 and 16, but these seem out of place.

*We cited the review paper and application papers suggested by the reviewer in the introduction and relocated the out-of-place citation.*

3. The authors cite dms_tools in kind of a weird place (ref 11) and don't actually list it with the other software packages. While it's fine that the authors didn't run it for thde paper, it should be listed along with the other software.

*We didn’t include dms_tools initially because the output of dms_tools is inherently very dif erent from the rest of the method. All the other methods treat a variant (including multiple mutations) as one identity and compute a score for each variant. In contrast, dms_tools assumes the linear addition of per amino acid ef ect, ignores epistasis coupling, and computes a score for each amino acid at every position; the final variant score would be the sum of such amino-acid score across positions. It is very similar to the one-hot encoding of amino-acid sequences in the neural network setting (such as Mochi by Lehner’s group).*

*The usage of dms_tools makes sense when the main theme of Bloom’s data is virus DMS by random mutation but has limited usage, for example, in the case of clinical variant identification while we have the human reference genome in mind, or the case of biophysical structure modeling of protein.*

*We described dms_tools in the introduction along with other methods and made this distinction.*

4. The authors cite two relatively recent papers for "multiple time points" and assert this is a recent innovation, but Starita et al. 2015 [PMID 25823446] did this relatively early in the history of the field, and this dataset was analyzed in the Enrich2 paper. The claim about this being a new innovation should be removed, and the authors should consider this and any other applicable citations.

*Thank you and we agree. We changed the wording and cited the paper. We also included the BRCA1-RING paper mentioned in our real data analysis.*

5. The authors mention analyzing DMS in a position-specific manner at the top of page 3 and cite a couple of very broad papers (one protocol paper and one very early review). The authors should choose and cite some specific studies that do this instead.

*We adjusted the citation and changed the wording.*

6. At the end of section 2.1 the authors mention being able to process data from Enrich2 "for other protocols." The most obvious application here is barcoded libraries, and I suggest that the authors add a phrase like ", such as barcoded sequencing libraries" at the end of this sentence. Barcoded libraries are fairly common, and it would be nice to point out how userds can analyze them using Rosace.

*We added the phrase. However, the issue with barcoded libraries is upstream of Rosace, with doing the barcode to variant mapping. After the variant mapping users can use the standard Rosace pipeline.*

7. In section 2.2, the authors don't talk about how indels are handled. It would be good to have a sentence there about how the model interprets in-frame insertions or deletions of a single amino acid (maybe it doesn't?), as this is a common feature of a lot of DMS libraries.

*We made it clear that Rosace could handle indel variants in Section 2.2 and the heuristics of mapping indel variants to their position labels are described in the second paragraph of Section 4.3*

8. In the Figure 1 legend, the authors should add something about the two different types of replicate structures show in panel B, with replication pre- and post- transfection. It's great that these are depicted, but there should be something in the legend.

*We added the description to the Figure 1B legend and caption.*

9. In the Figure 2 legend, the plate diagram (panel C) is not interpretable without a description of parameters, but the authors ask the reader to consult the methods section. I would like the authors to either put some brief definitions here or move the plate diagram out of this figure and into the methods section near the parameter definitions.

*We’ve added a description of the parameters in Figure 2C.*

10. At the end of section 2.2 the authors mention the "MSH data". This should be "MSH2 data". *Fixed.*

11. Sometimes the authors write tools as mutscan(edgeR) and mutscan(limma) or mutscan(limma-voom), and other times they just write edgeR or limma-voom. I suggest that these always be written as part of mutscan for clarity.

*Fixed.*

12. In the discussion, the authors refer to "FACS-seq" but this is predated by VAMP-seq and VAMP-seq should be cited and mentioned here.

*We cited VAMP-seq both in Introduction and Discussion.*

13. The last paragraph/sentence of section 4.1 is duplicated above at the end of the first paragraph in the section.

*Fixed, thanks!*

**2^nd^ round**

**Reviewer 1**

I appreciate the authors' attempt to address my comments and those of the other reviewer. The manuscript is considerably improved with the new DMS datasets, analyses and deeper discussion of potential caveats and issues. I have no further comments, and I hope that their methods will provide value for the community.

**Reviewer 2**

I thank the authors for their thoughtful responses and thorough incorporation of the reviewer feedback. All my concerns have been addressed, and I was able to successfully run the example vignette using the Docker container.

One extremely minor comment is that the reference 43 to the Zenodo archive of the code should probably mention Zenodo and include the DOI. The citation tools on the associated page (https://zenodo.org/records/10814911) can help with this.
